# Supplementary material for: The homologous recombination factors BRCA2 and PALB2 interplay with mismatch repair pathways to maintain centromere stability and cell viability
Source: Cell Rep. 2025 Jan 31;44(2):115259. doi: 10.1016/j.celrep.2025.115259 (PMC11860765; doi:10.1016/j.celrep.2025.115259)
Supplement: Document S1. Figures S1–S7 [file mmc1.pdf]

Cell Reports, Volume *44*

Supplemental information

The homologous recombination factors BRCA2 and PALB2 interplay with mismatch repair pathways to maintain centromere stability and cell viability

Emily Graham, Lucia Rampazzo, Chin Wei Brian Leung, Jacob Wall, Emőke Zsanett Gerőcz, Mikhail Liskovykh, Nikolay Goncharov, Xanita Saayman, Ramazan Gundogdu, Masato T. Kanemaki, Hiroshi Masumoto, Vladimir Larionov, Natalay Kouprina, and Fumiko Esashi

Supplemental Figures

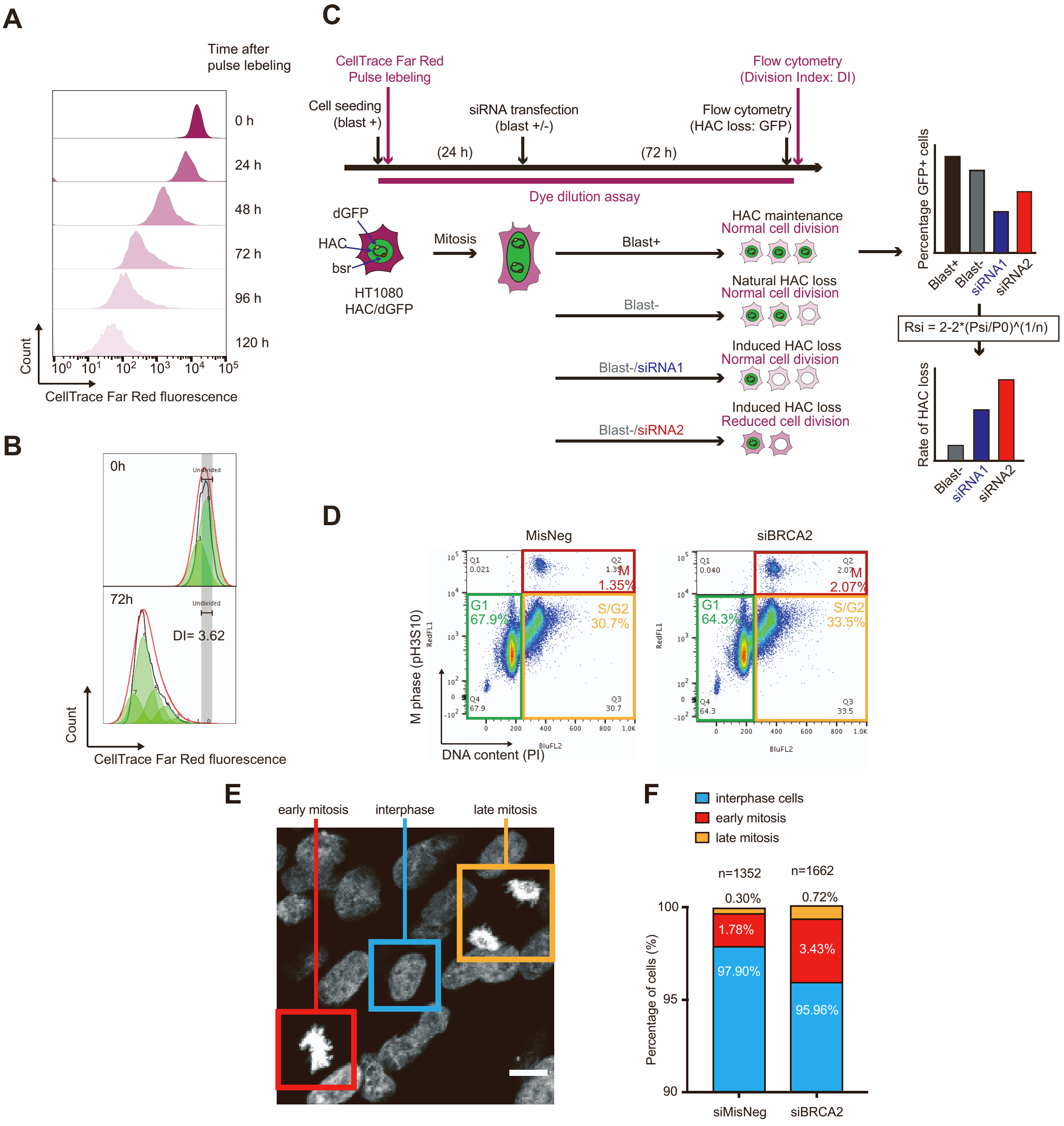

Figure S1. Cell cycle effect upon BRCA2 depletion in HT1080 cells, Related to Figure 1

A. Flow cytometry dye dilution assay proof of principle. Fluorescent intensity of the cell dye is diluted with every cell division. **B.** Representative image of the DI determined from the dye dilution assay. **C.** Schematic of HAC loss assay combined with the dye dilution assay. Blasticidin-containing medium selects for HAC-containing cells. 72 hours post-siRNA treatment, GFP signal was measured by flow cytometry. **D.** Cell cycle profile upon 72 hours siMisNeg and siBRCA2 treatment after staining with Propidium iodide (PI) and pH3 S10. **E.** Representative image of DAPI staining in HT1080 alphoid^{tetO} dGFP-HAC cells, 72 hours post BRCA2 depletion. Interphase (blue), early mitosis (red), late mitosis (yellow). Scale bar equals 10 µm. **F.** Graph displaying the percentage of cells in different stages of cell cycle in control and BRCA2-depleted cells. n is the number of cells analysed for each condition in a single experiment.

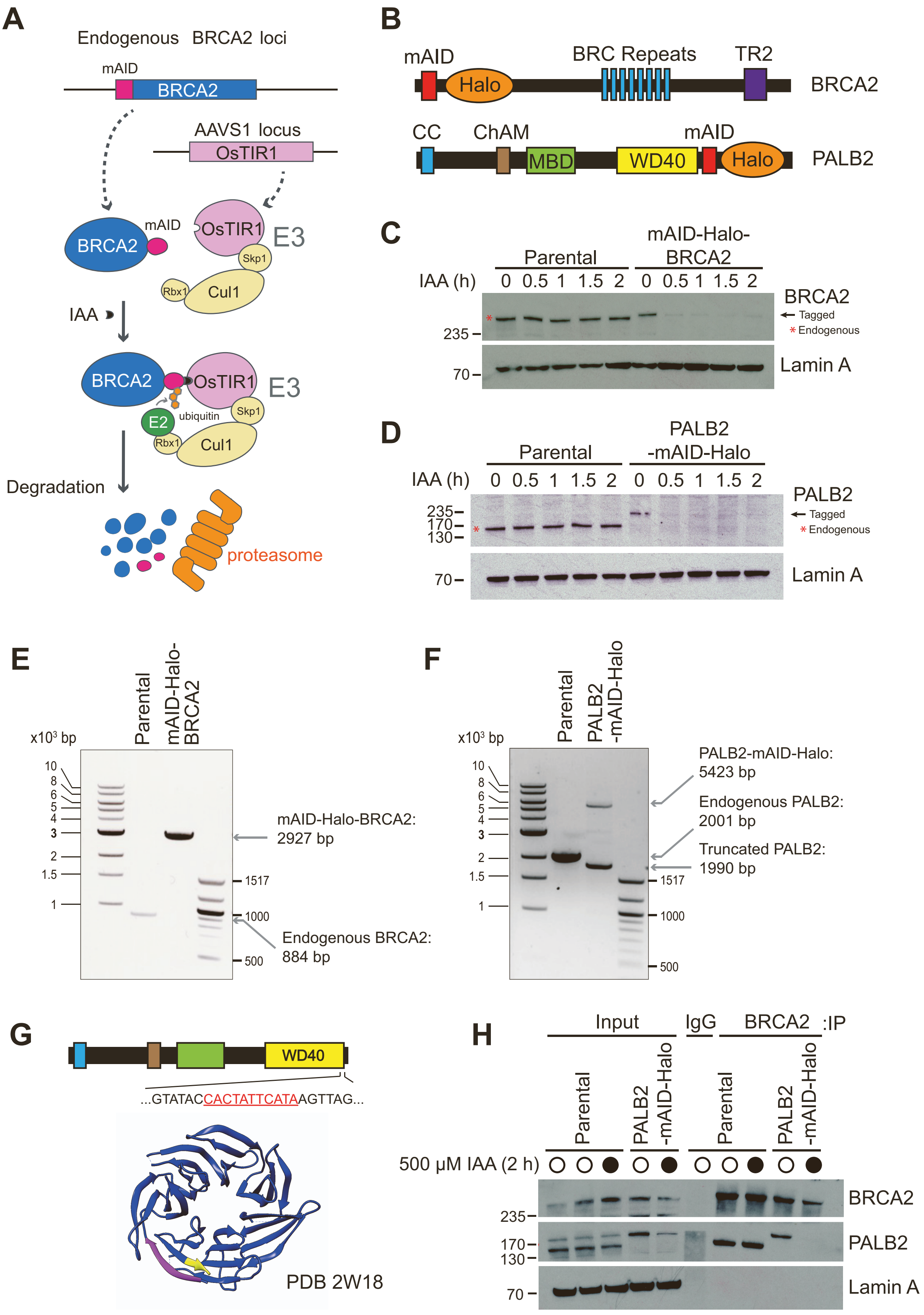

Figure S2. Generation and validation of auxin-inducible degron (AID) system for depleting BRCA2 or PALB2 in HCT116 cells, Related to Figures 2 and 3

A. Schematic of auxin-inducible degron (AID) system to degrade proteins of interest. **B.** Schematic of BRCA2 and PALB2 mAID-Halo tagging. mAID, mini Auxin-inducible degron. CC, Coiled-coil. ChAM, Chromatin-association motif. MBD, MRG15-binding domain. **C.** Western blot showing degradation of BRCA2 within 30 minutes of 500 μM IAA treatment. Lamin A used as a loading control. **D.** Western blot showing degradation of PALB2 within 30 minutes of 500 μM IAA treatment. Red asterisk refers to endogenous PALB2 protein. Lamin A used as a loading control. **E-F.** Agarose gel image of PCR validating N-terminal tagging of BRCA2 (E) or C-terminal tagging of PALB2 (F) with mAID and Halo. The expected size of each amplified DNA fragments are displayed. Asterisk marks band sent for sequencing. **G.** Schematic of the deleted allele of PALB2 gene, where the deleted nucleotides are indicated in red. Crystal structure of PALB2 WD40 domain shows that C-terminal three amino acids (yellow highlight) interact with the N-terminal beta-sheet (pink highlight) for stability, hence the truncation destabilises the WD40 domain and full-length PALB2. **H.** Immunoprecipitation showing loss of PALB2-BRCA2 interaction upon 2 hours 500 μM IAA treatment. Lamin A used as a loading control for the inputs.

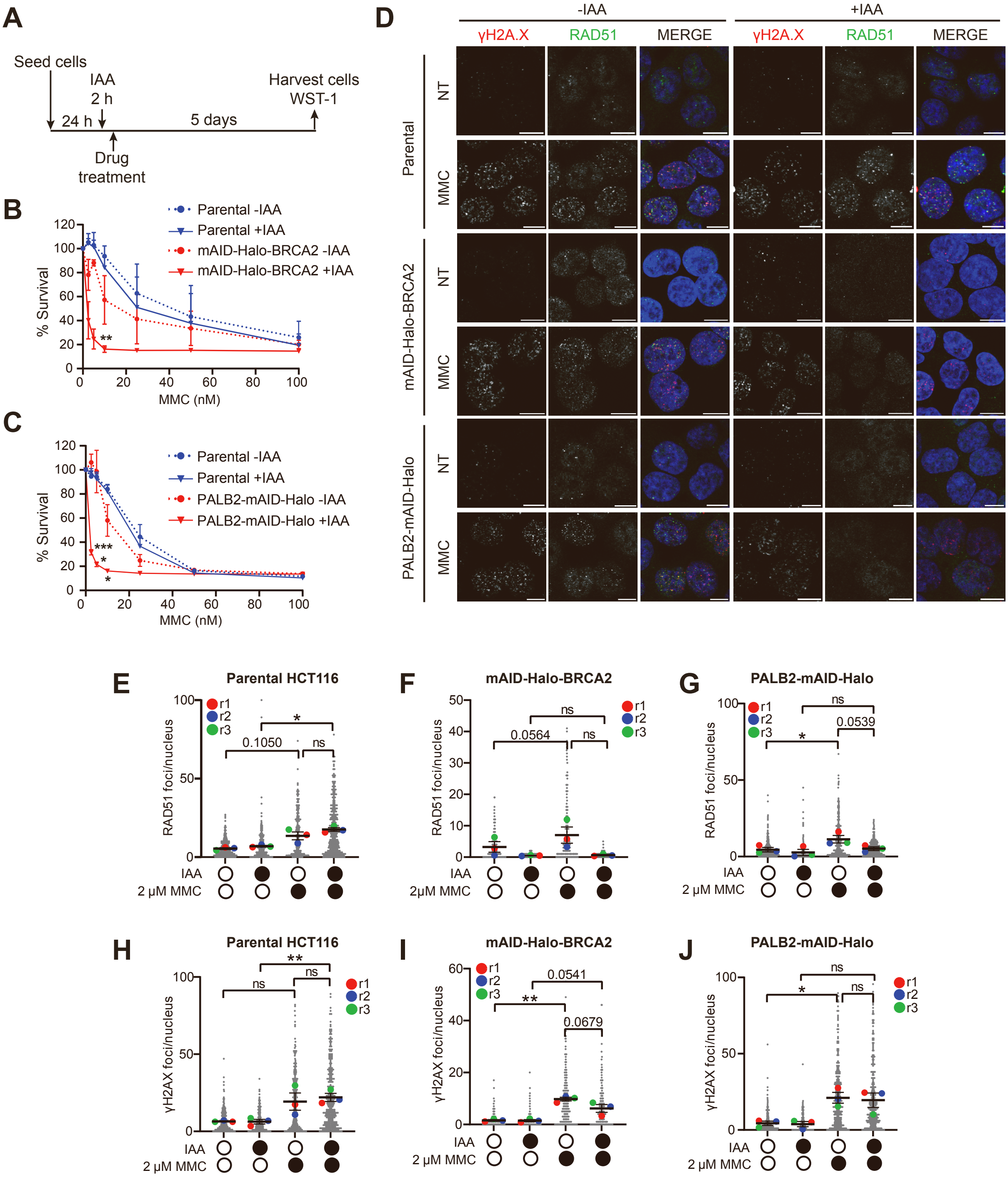

Figure S3. AID-mediated BRCA2 and PALB2 depletion sensitises HCT116 cells to DNA-damaging agents, Related to Figures 2 and 3

A. Schematic of WST-1 protocol. **B.** Graph showing percentage cell survival after MMC and 500 μM IAA treatment in HCT116 *Os*TIR1 parental and mAID-BRCA2 cell lines. Points represent average of three independent experiments. Error bars represent S.E.M. Two-tailed T-test comparing -IAA vs +IAA for each cell line. **C.** Graph showing percentage cell survival after MMC and 500 μM IAA treatment in HCT116 *Os*TIR1 parental and PALB2-mAID cell lines. Points represent average of three independent experiments. Error bars represent S.E.M. Two-tailed t-test comparing -IAA vs +IAA for each cell line. **D.** Representative images RAD51 and γ-H2A.X signalling after BRCA2 and PALB2 depletion in HCT116 *Os*TIR1 degron cells. Scale bar equals 10 μm. **E-G.** Quantification of RAD51 foci count per nucleus after MMC treatment in HCT116 *Os*TIR1 parental (panel E), mAID-BRCA2 (panel F), and PALB2-mAID cells (panel G). Cells were first treated with IAA for 2 hours, followed by MMC treatment

for 2 hours. At least 100 cells were quantified per condition, per repeat. Error bars represent S.E.M. Paired t-test. **H-J.** Quantification of γ-H2A.X foci count per nucleus after MMC treatment in HCT116 *Os*TIR1 parental (panel H), mAID-BRCA2 (panel I), and PALB2-mAID cells (panel J). Cells were first treated with IAA for 2 hours, followed by MMC treatment for 2 hours. At least 100 cells were quantified per condition, per repeat. Error bars represent S.E.M. Paired t-test. ns represents no statistical significance; $p<0.05$ (*), $p<0.01$ (**), $p<0.001$ (***).

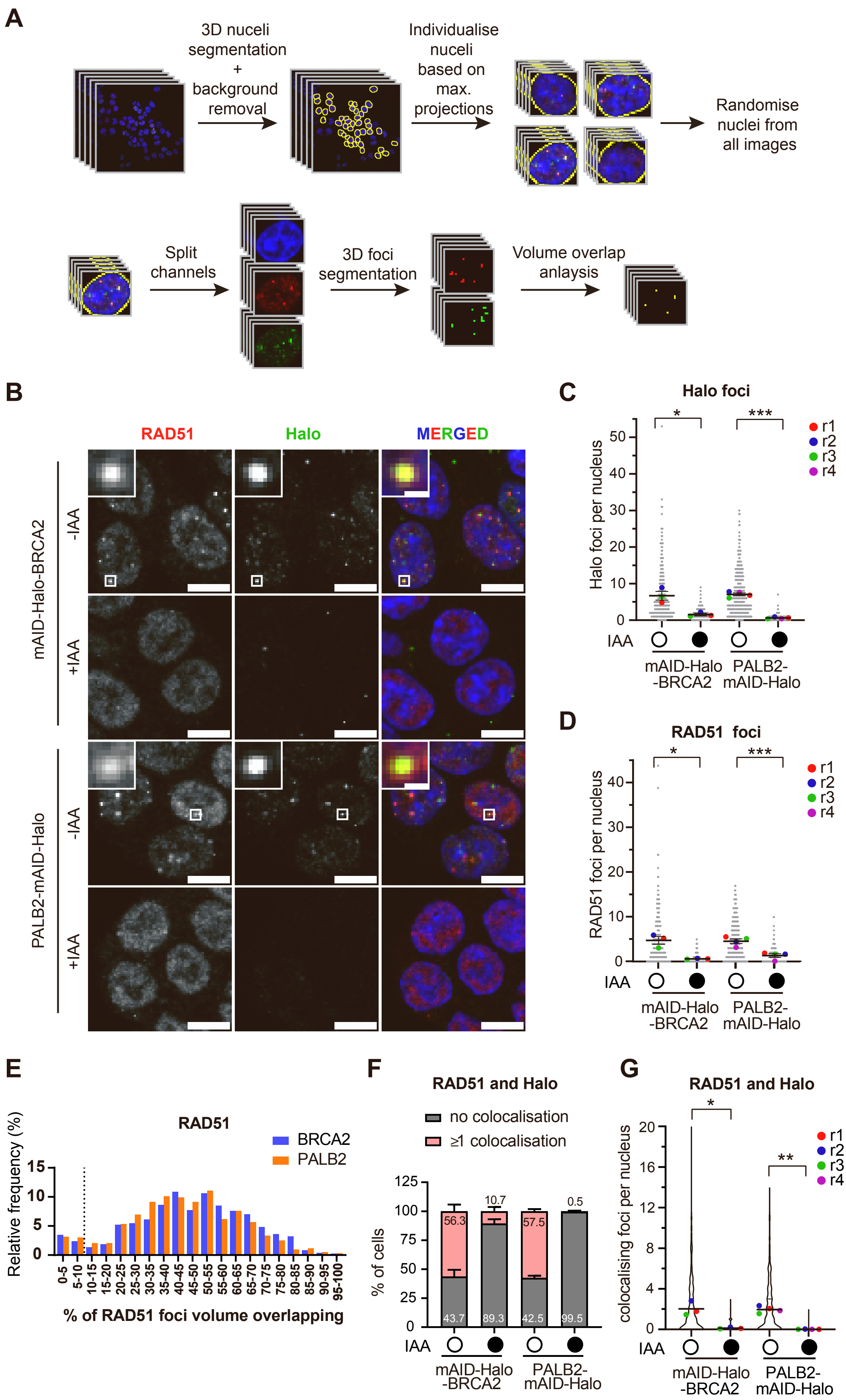

A
3D nuceli segmentation + background removal
Individualise nuceli based on max. projections
Randomise nuclei from all images
Split channels
3D foci segmentation
Volume overlap anlaysis
B
RAD51
Halo
MERGED
mAID-Halo-BRCA2
PALB2-mAID-Halo
-IAA
+IAA
C
Halo foci
Halo foci per nucleus
IAA
mAID-Halo -BRCA2
PALB2- mAID-Halo
r1
r2
r3
r4
D
RAD51 foci
RAD51 foci per nucleus
E
RAD51
BRCA2
PALB2
Relative frequency (%)
% of RAD51 foci volume overlapping
F
RAD51 and Halo
no colocalisation
≥1 colocalisation
% of cells
56.3
10.7
57.5
0.5
43.7
89.3
42.5
99.5
G
RAD51 and Halo
colocalising foci per nucleus

Figure S4. Visualisation of endogenous BRCA2 and PALB2 using the HaloTag, Related to Figures 2 and 3

A. Schematic of the volume overlap analysis pipeline. **B.** Representative images of Halo ligand JF549 (green) and RAD51 (red) in HCT116 mAID-Halo-BRCA2 and PALB2-mAID-Halo cell lines. DNA staining by Hoechst shown in blue. Scale bar equals to 5 µm, inset scale bar equals to 1 µm). **C.** Number of Halo foci after IAA-mediated depletion using 3D segmentation pipeline. Each datapoint represents one nucleus, coloured dots are means of the replicates. Error bars represent S.E.M. Paired t-test between the means of 3 replicates. **D.** Number of RAD51 foci detected using 3D segmentation pipeline. Each datapoint represents one nucleus, coloured dots are means of the replicates. Error bars represent S.E.M. Paired t-test between the means of 3 replicates. **E.** Percentage of RAD51 focus volume overlapping with Halo-BRCA2 (blue) and PALB2-Halo (orange) foci. Values represent the relative frequency of the amount of overlap from all colocalisation events from the non-treated condition. Data also displayed in Figure 2E. **F.** Percentage of nuclei with none or at least one colocalisation event between CENP-B and Halo. At least 3 biological replicates with 100 cells analysed in each condition per replicate. Error bars represent S.E.M. **G.** Number of colocalisation events per nuclei. Coloured dots represent the means of the replicates. Error bars represent S.E.M. Paired t-test. ns represents no statistical significance; $p<0.05$ (*), $p<0.01$ (**), $p<0.001$ (***).

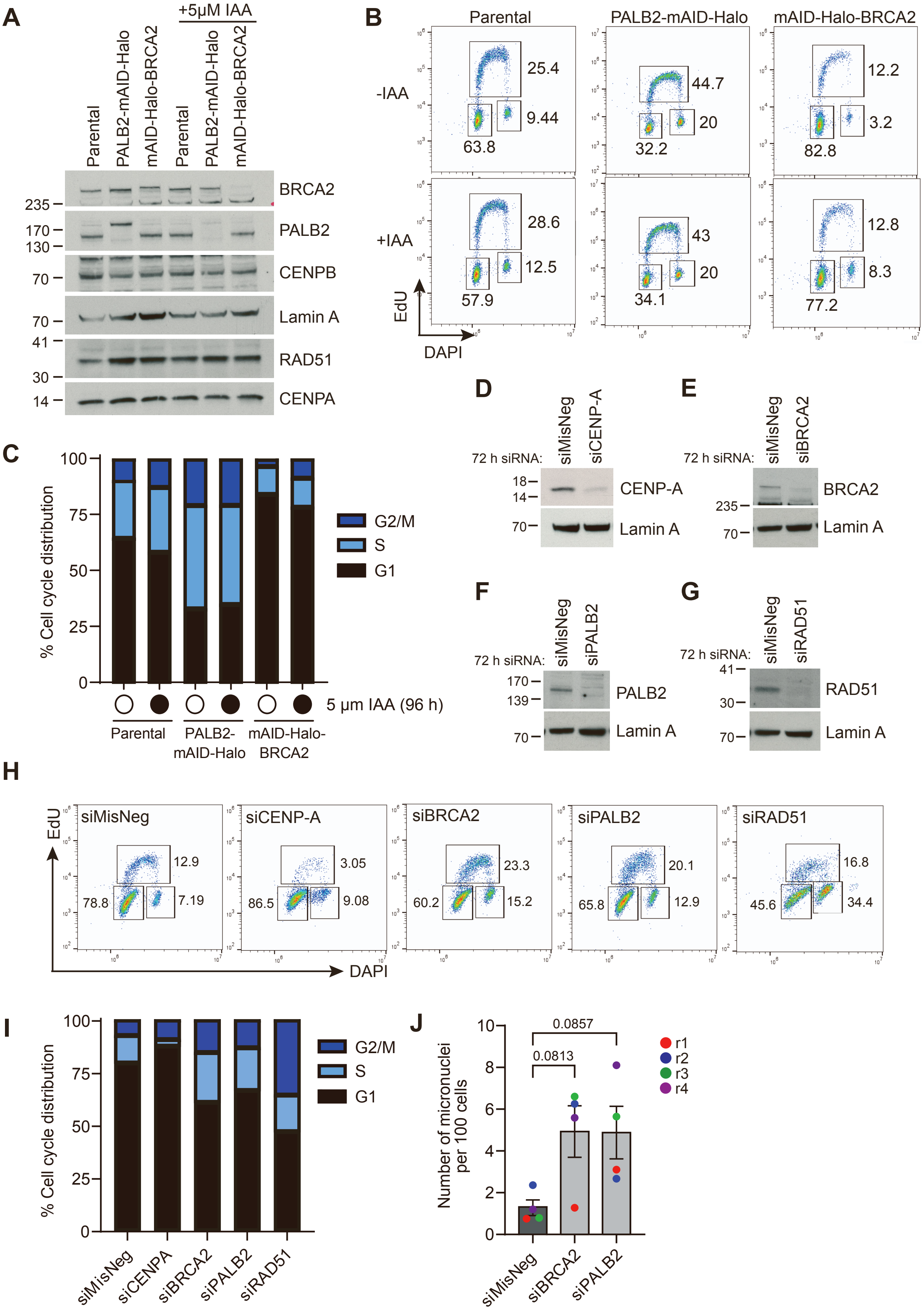

Figure S5. The depletion of BRCA2 or PALB2 confer little impact on cell cycle progression, Related to Figures 4 and 5

A. Western blot showing PALB2 and BRCA2 degradation after 5 μM IAA treatment for 96 hours in HCT116 *Os*TIR1 cells. Lamin A used as a loading control. **B.** Cell cycle analysis by flow cytometry after 96 hours 5 μM IAA treatment and EdU and DAPI staining in HCT116 *Os*TIR1 cells. Cells in G1, S, and G2/M phase are gated and percentage of cells in each gate displayed. **C.** Graph displaying quantification of the percentage of cells in each cell cycle stage after IAA depletion, as shown in panel B. **D-G.** Depletion of CENP-A (panel D), BRCA2 (panel E), PALB2 (panel F), and RAD51 (panel G) in RPE1 cells using siRNA for 72 hours. siMisNeg was used as a negative control. Lamin A was used as a loading control. **H.** Cell cycle analysis by flow cytometry of RPE1 cells after 96 hours siRNA depletion and EdU and DAPI staining. Cells in G1, S, and G2/M phase are gated and percentage of cells in each gate displayed. **I.** Graph displaying quantification of the percentage of cells in each cell cycle stage after siRNA depletion, as shown in panel H **J.** Number of micronuclei per 100 cells of RPE1 cells after siRNA-mediated depletion of BRCA2 and PALB2. At least 100 cells analysed per condition, per repeat. Error bars represent S.E.M. RM one-way ANOVA with Tukey's multiple comparisons test.

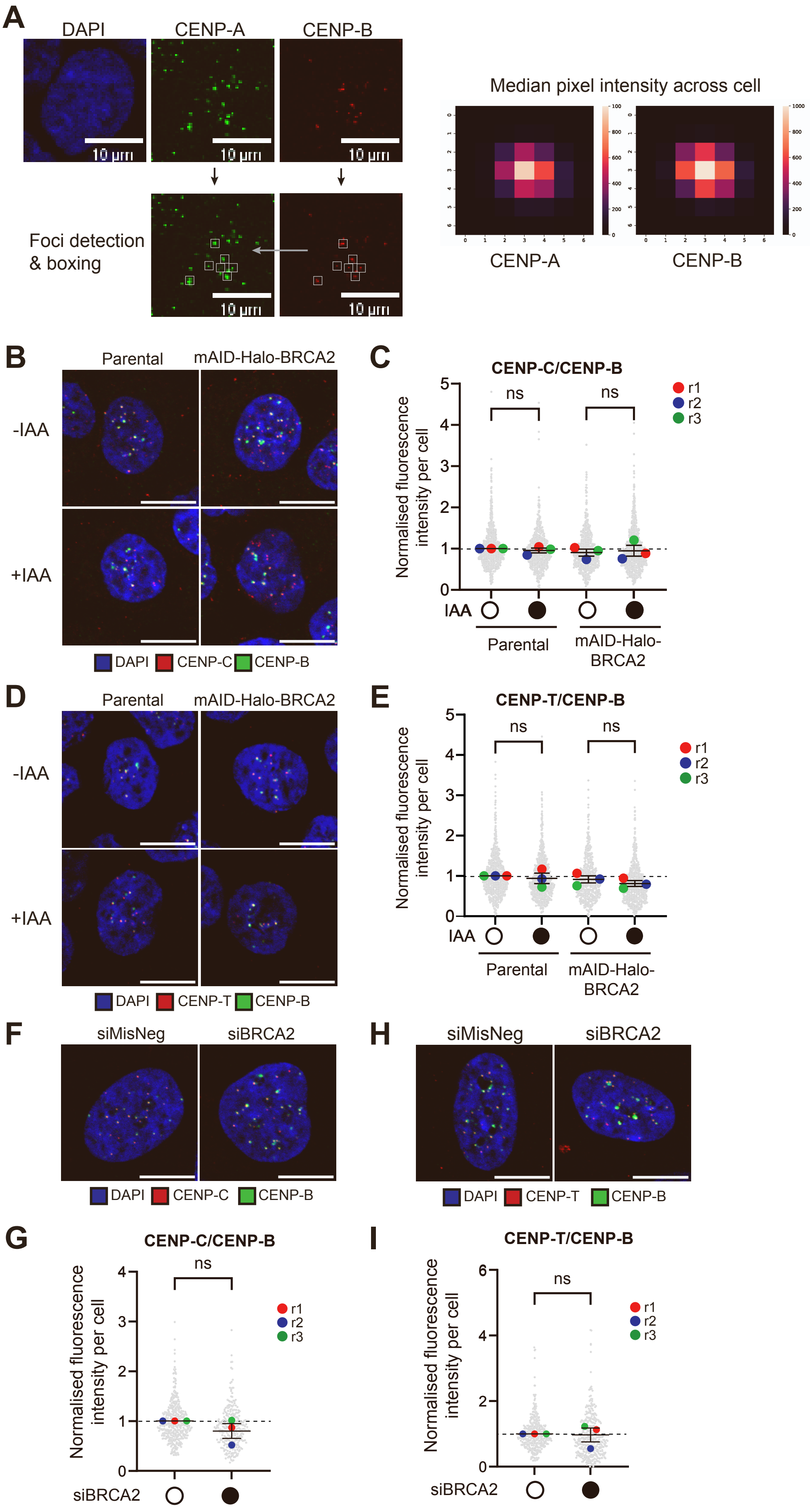

A
DAPI
CENP-A
CENP-B
10 µm
Foci detection & boxing
Median pixel intensity across cell
CENP-A
CENP-B
B
Parental
mAID-Halo-BRCA2
-IAA
+IAA
DAPI CENP-C CENP-B
C
CENP-C/CENP-B
ns
ns
Normalised fluorescence intensity per cell
IAA
Parental
mAID-Halo-BRCA2
r1
r2
r3
D
Parental
mAID-Halo-BRCA2
-IAA
+IAA
DAPI CENP-T CENP-B
E
CENP-T/CENP-B
ns
ns
Normalised fluorescence intensity per cell
IAA
Parental
mAID-Halo-BRCA2
r1
r2
r3
F
siMisNeg
siBRCA2
DAPI CENP-C CENP-B
H
siMisNeg
siBRCA2
DAPI CENP-T CENP-B
G
CENP-C/CENP-B
ns
Normalised fluorescence intensity per cell
siBRCA2
r1
r2
r3
I
CENP-T/CENP-B
ns
Normalised fluorescence intensity per cell
siBRCA2
r1
r2
r3

Figure S6. BRCA2 depletion confers no detectable impact on the levels of CENP-C and CENP-T at centromeres, Related to Figure 4

A. Schematic of centromere component quantification. **B.** Representative images of CENP-C and CENP-B immunofluorescence staining in HCT116 *Os*TIR1 parental or mAID-Halo-BRCA2 cells, with or without treatment of IAA. Scale bar equals 10 μm. **C.** Normalised CENP-C to CENP-B signal in HCT116 *Os*TIR1 parental or mAID-Halo-BRCA2 cells. RM one-way ANOVA with Tukey's multiple comparisons test. **D.** Representative images of CENP-T and CENP-B immunofluorescence staining in HCT116 *Os*TIR1 parental or mAID-Halo-BRCA2 cells, with or without treatment of IAA. Scale bar equals 10 μm. **E.** Normalised CENP-T to CENP-B signal in parental or mAID-Halo-BRCA2 HCT116 cells. RM one-way ANOVA with Tukey's multiple comparisons test. **F.** Representative images of CENP-C and CENP-B immunofluorescence staining in RPE1 cells with 96 hours siRNA treatment. Scale bar equals 10 μm. **G.** Normalised CENP-C signal to CENP-B in RPE1 cells after siRNA treatment, 96 hours. Paired t-test. **H.** Representative images of CENP-T and CENP-B immunofluorescence staining in RPE1 cells with 96 hours siRNA treatment. Scale bar equals 10 μm. **I.** Normalised CENP-T signal to CENP-B in RPE1 cells after 96 hours siRNA treatment. For each measurement, signal was normalised to the siMisNeg control. n=3. At least 100 cells analysed per condition, per repeat. Large coloured dots represent the mean of each repeat. Error bars represent S.E.M. Paired t-test. ns represents no statistical significance.

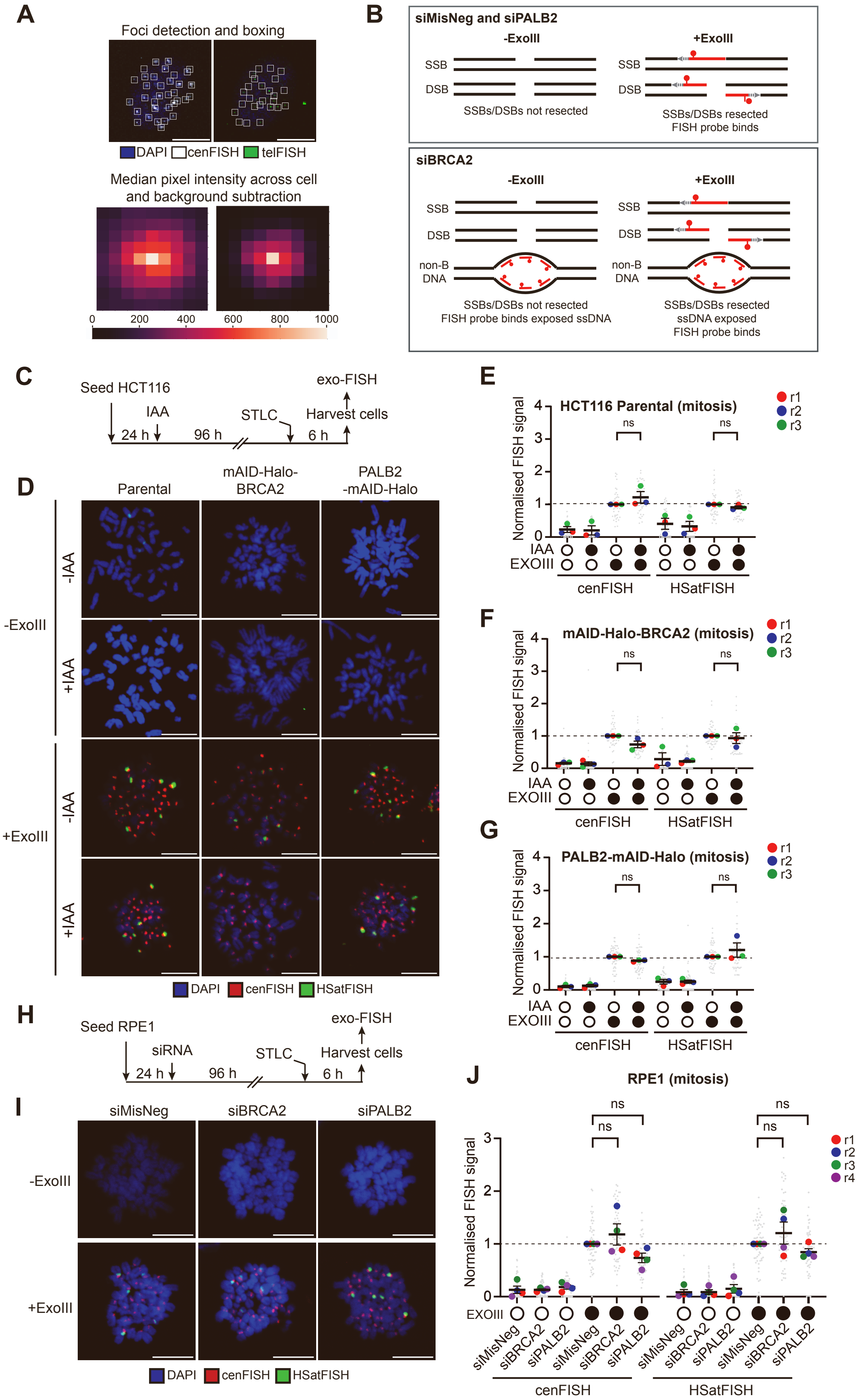
A
Foci detection and boxing
DAPI
cenFISH
telFISH
Median pixel intensity across cell and background subtraction
0
200
400
600
800
1000
B
siMisNeg and siPALB2
-ExoIII
+ExoIII
SSB
DSB
SSBs/DSBs not resected
SSBs/DSBs resected FISH probe binds
siBRCA2
-ExoIII
+ExoIII
SSB
DSB
non-B DNA
SSBs/DSBs not resected FISH probe binds exposed ssDNA
SSBs/DSBs resected ssDNA exposed FISH probe binds
C
Seed HCT116
IAA
24 h
96 h
STLC
6 h
Harvest cells
exo-FISH
D
Parental
mAID-Halo-BRCA2
PALB2 -mAID-Halo
-ExoIII
+ExoIII
-IAA
+IAA
DAPI
cenFISH
HSatFISH
E
HCT116 Parental (mitosis)
Normalised FISH signal
ns
r1
r2
r3
IAA
EXOIII
cenFISH
HSatFISH
F
mAID-Halo-BRCA2 (mitosis)
Normalised FISH signal
ns
r1
r2
r3
IAA
EXOIII
cenFISH
HSatFISH
G
PALB2-mAID-Halo (mitosis)
Normalised FISH signal
ns
r1
r2
r3
IAA
EXOIII
cenFISH
HSatFISH
H
Seed RPE1
siRNA
24 h
96 h
STLC
6 h
Harvest cells
exo-FISH
I
siMisNeg
siBRCA2
siPALB2
-ExoIII
+ExoIII
DAPI
cenFISH
HSatFISH
J
RPE1 (mitosis)
Normalised FISH signal
ns
r1
r2
r3
r4
EXOIII
siMisNeg
siBRCA2
siPALB2
cenFISH
HSatFISH

Figure S7. The depletion of BRCA2 or PALB2 confer no detectable impact on centromere DNA breaks in mitosis, Related to Figure 5

A. Workflow of exo-FISH analysis. Scale bar equals 10 μm. **B.** Model showing source of FISH signal under different conditions in siRNA-treated RPE1 cells. In siMisNeg or siPALB2 treated samples, FISH signal is low in -ExoIII conditions, reflecting no resection of ssDNA/dsDNA. Upon ExoIII treatment, breaks are resected and resultant ssDNA is bound by cenFISH probes. In siBRCA2 treated samples, FISH signal is high even in the absence of ExoIII treatment, potentially reflecting the accumulation of secondary DNA structures, such as DNA bubbles and non-B form DNA, exposing ssDNA for FISH probe binding. Upon ExoIII treatment, DNA breaks are resected, and FISH probes bind. Secondary DNA structures are break-independent and unaffected by ExoIII treatment. **C.** Schematic of experimental protocol for exo-FISH in mitotic HCT116 *Os*TIR1 degron cells. **D.** Representative images of exo-FISH. Scale bar equals 10 μm. **E.** exo-FISH in HCT116 *Os*TIR1 parental cells with or without 5 μM IAA for 96 hours. RM one-way ANOVA with Tukey's multiple comparisons test. **F.** exo-FISH in HCT116 mAID-BRCA2 cells with or without 5 μM IAA for 96 hours. RM one-way ANOVA with Tukey's multiple comparisons test. **G.** exo-FISH in HCT116 mAID-PALB2 cells with or without 5 μM IAA for 96 hours. cenFISH and HSatFISH signal normalised to +IAA, +ExoIII treated samples. Large coloured dots represent average of each independent experiment. n=3. At least 15 cells analysed per condition, per repeat. Error bars represent S.E.M. RM one-way ANOVA with Tukey's multiple comparisons test. **H.** Schematic of siRNA treatment protocol for exo-FISH in mitotic RPE1 cells. **I.** Representative images of exo-FISH after 96 hours siRNA treatment in RPE1 cells. Scale bar equals 10 μm. **J.** exo-FISH of mitotic RPE1 cells with or without BRCA2 or PALB2 siRNA mediated depletion. Samples were normalised to siMisNeg cenFISH or HSatFISH signal without ExoIII treatment. Large coloured datapoints represent the averages of each independent repeat. n=3. At least 15 cells analysed per condition, per repeat. Error bars represent S.E.M. RM one-way ANOVA with Tukey's multiple comparisons test. ns represents no statistical significance.
