# Supplementary material for: The homologous recombination factors BRCA2 and PALB2 interplay with mismatch repair pathways to maintain centromere stability and cell viability
Source: Cell Rep. 2025 Jan 31;44(2):115259. doi: 10.1016/j.celrep.2025.115259 (PMC11860765; doi:10.1016/j.celrep.2025.115259)
Supplement: Document S2. Article plus supplemental information [file mmc2.pdf]

Cell Reports

Article

The homologous recombination factors BRCA2 and PALB2 interplay with mismatch repair pathways to maintain centromere stability and cell viability

Graphical abstract

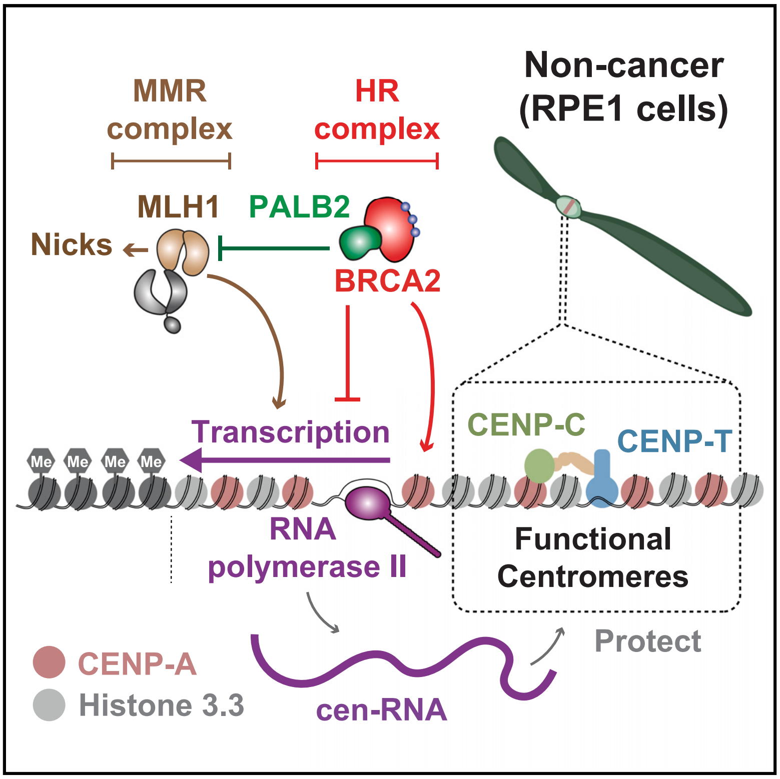

Authors

Emily Graham, Lucia Rampazzo, Chin Wei Brian Leung, ..., Vladimir Larionov, Natalay Kouprina, Fumiko Esashi

Correspondence

fumiko.esashi@path.ox.ac.uk

In brief

Graham et al. demonstrate that BRCA2 and PALB2 protect centromere integrity in non-cancerous cells through distinct mechanisms, partly counteracting the mismatch repair factor MLH1. In cancer cells, BRCA2 remains essential for centromere protection, highlighting its critical role in preventing chromosome instability and the interplay between homologous recombination and mismatch repair pathways.

Highlights

- BRCA2 ensures centromere stability by maintaining CENP-A and preventing fragility
- PALB2 prevents centromeric DNA breaks under non-cancerous conditions
- MLH1 loss conceals centromere fragility arising from BRCA2 or PALB2 deficiency
- HR and MMR factors jointly support centromere resilience and cell survival

Graham et al., 2025, Cell Reports *44*, 115259
February 25, 2025 © 2025 The Authors. Published by Elsevier Inc.
https://doi.org/10.1016/j.celrep.2025.115259

CellPress

Cell Reports

CellPress
OPEN ACCESS

Article

The homologous recombination factors BRCA2 and PALB2 interplay with mismatch repair pathways to maintain centromere stability and cell viability

Emily Graham,[1,8] Lucia Rampazzo,[1,8] Chin Wei Brian Leung,[1,9] Jacob Wall,[1,9] Emőke Zsanett Gerőcz,[1] Mikhail Liskovykh,[2] Nikolay Goncharov,[2] Xanita Saayman,[1] Ramazan Gundogdu,[1,3] Masato T. Kanemaki,[4,5,6] Hiroshi Masumoto,[7] Vladimir Larionov,[2] Natalay Kouprina,[2] and Fumiko Esashi[1,10,*]

[1]Sir William Dunn School of Pathology, University of Oxford, Oxford, UK
[2]Developmental Therapeutics Branch, National Cancer Institute, National Institutes of Health, Bethesda, MD 20892, USA
[3]Department of Pharmacy Services, Vocational School of Health Services, Bingol University, Bingol, Türkiye
[4]Department of Chromosome Science, National Institute of Genetics, Research Organization of Information and Systems (ROIS), Shizuoka, Japan
[5]Department of Advanced Studies, SOKENDAI, Shizuoka, Japan
[6]Department of Biological Sciences, Graduate School of Science, The University of Tokyo, Tokyo, Japan
[7]Laboratory of Chromosome Engineering, Department of Frontier Research and Development, Kazusa DNA Research Institute, Kisarazu, Chiba 292-0818d, Japan
[8]These authors contributed equally
[9]These authors contributed equally
[10]Lead contact
*Correspondence: fumiko.esashi@path.ox.ac.uk
https://doi.org/10.1016/j.celrep.2025.115259

SUMMARY

Centromeres are crucial for chromosome segregation but are vulnerable to breakage and recombination due to their repetitive DNA. The mechanisms protecting centromeres from these instabilities remain incompletely understood. This study investigates the role of the homologous recombination (HR) mediators BRCA2 and PALB2 in centromere stability. We find that BRCA2, but not PALB2, is essential for maintaining a human artificial chromosome. In native chromosomes, BRCA2 ensures CENP-A occupancy and prevents DNA fragility at centromeres. Conversely, PALB2 does not affect CENP-A, whereas its depletion increases centromeric DNA breaks in non-cancerous cells only. Interestingly, depleting the mismatch repair (MMR) factor MLH1 masks centromere fragility caused by BRCA2 or PALB2 loss, suggesting that MLH1 contributes to DNA instability when BRCA2 or PALB2 is absent. However, cells deficient in both BRCA2/PALB2 and MLH1 have reduced survival. These results highlight a critical balance between HR and MMR factors in preserving centromere integrity and cell viability.

INTRODUCTION

Centromeres are structural regions of the genome comprised of tandem α-satellite repeats spanning several megabases and play an essential role in transmitting chromosomes to daughter cells during mitosis. This functionality is determined by inheritable chromatin features, independent of DNA sequence, through the epigenetic incorporation of the histone H3 variant centromere protein A (CENP-A) into a small subset of α-satellite repeats. CENP-A at centromeres acts as a platform for assembly of the constitutive centromere-associated network (CCAN) and the outer kinetochore machinery that separates sister chromatids during mitosis.[1] While the repetitiveness of centromeres is considered to provide the robustness of their functionality, there is increasing recognition that centromeres are highly susceptible to DNA breaks and aberrant recombination due to their repetitive non-B-form structures, including stem loops, hairpins, and R-loops.[2–6] These fragilities are proposed to be associated with DNA replication, mitotic chromosome segregation, or transcription,[5,7,8] while the mechanism protecting centromeres from these threats is not fully understood.

The tumor suppressor breast cancer 2 (*BRCA2*) underlies hereditary breast and ovarian cancer (HBOC) syndrome, conferring a high risk of developing several cancers, including breast, ovarian, and pancreatic cancer.[9–13] Biallelic inheritance of pathogenic *BRCA2* mutations confers severe consequences, such as embryonic lethality or development of the rare genetic disorder Fanconi anemia (FA), characterized by bone marrow failure, physical abnormalities, organ defects, and childhood cancers.[14] Cells from affected individuals display aneuploidy, with aberrant numbers and structures of chromosomes, collectively known as chromosome instability (CIN).[15–17] CIN phenotypes in BRCA2-defective cells are commonly explained by BRCA2's canonical role in the repair of DNA double-strand breaks (DSBs) by

© 2025 The Authors. Published by Elsevier Inc.
This is an open access article under the CC BY license (http://creativecommons.org/licenses/by/4.0/).

homologous recombination (HR). This role of BRCA2 is mediated by its interaction with the essential RAD51 recombinase and the product of the partner and localizer of BRCA2 (*PALB2*) gene, mutations of which are also associated with HBOC and FA.[18]

In this study, we conducted a systematic analysis to evaluate the roles of BRCA2 and PALB2 at centromeres in normally growing, near-diploid cancerous and non-cancerous human cells. First, experiments using a human artificial chromosome (HAC) model in HT1080 fibrosarcoma cells demonstrated that BRCA2, but not PALB2, plays a crucial role in maintaining HAC. Second, conditional depletion of BRCA2 and PALB2 in colorectal carcinoma HCT116 cells results in distinct chromosomal aberrations. By assessing centromere properties in these corresponding HCT116 cells and human telomerase reverse transcriptase -immortalized non-cancerous retinal pigment epithelial 1 (RPE1) cells, we found that BRCA2, but not PALB2, is consistently crucial for maintaining CENP-A occupancy at centromeres. The accumulation of aberrant centromeric DNA structures was also detected after depletion of BRCA2 in both HCT116 and RPE1 cells, whereas PALB2 depletion conferred an increase in centromeric breaks only in RPE1 cells but not in HCT116 cells. Significantly, aberrant centromere structures found in BRCA2-or PALB2-depleted RPE1 cells are concealed when MLH1, a mismatch repair factor that is defective in HCT116 cells, is removed. Furthermore, depletion of BRCA2, but not PALB2, increased the level of centromeric transcription in RPE1 cells but not in HCT116 cells. Based on these observations, we propose that BRCA2 and PALB2 act distinctly at centromeres to limit the accumulation of aberrant DNA structures, at least in part by counteracting the action of mismatch repair (MMR), which corrects mismatched nucleotides at the cost of potentially introducing undesired DNA breaks or overdriving transcription.

RESULTS

BRCA2 loss, but not PALB2 loss, compromises HAC maintenance in HT1080 cells

To gain first insight into the role of HR factors in CIN, we used HT1080 fibrosarcoma cells, which have a relatively stable genome among cancer cell lines,[19] carrying alphoid^{tetO} HAC/destabilized green fluorescent protein (dGFP).[20] This HAC consists of synthetic centromere consensus DNA sequences from human chromosome 17 (chr17), including the 17-bp CENP-B box to which CENP-B binds,[21] and artificially introduced tetracycline operator (tetO) sequences, arranged in tandem arrays.[22–24] The alphoid^{tetO} HAC mimics endogenous chromosomes but lacks essential survival genes, allowing unbiased examination of factors required for its maintenance. Its stability is ~10-fold lower than that of a natural chromosome, providing a sensitive system to study CIN.[25] The HAC additionally contains cell cycle reporter GFP fusions, GFP/chromatin licensing and DNA replication factor 1 (CDT1) and GFP-Geminin, which are expressed in G1 and S/G2/early mitosis, respectively. This enables the assessment of HAC-containing cells by quantifying GFP-positive cells by flow cytometry.[20] These GFP fusions exhibit high turnover rates outside of the corresponding cell cycle stages, and their rapidly diminishing fluorescence upon HAC loss allows for the timely detection of protein depletion effects on HAC maintenance.[20] Using this highly sensitive system, we examined how BRCA2, PALB2, or RAD51 depletion affects the rate of HAC loss, defined as the probability of losing the HAC after *n* cell divisions.[20,25]

As a starting point, we determined the optimal time to treat cells with small interfering RNA (siRNA) targeting BRCA2, PALB2, or RAD51 (hereafter referred to as siBRCA2, siPALB2, or siRAD51, respectively) for HAC loss measurement. Almost complete depletion of BRCA2, PALB2, or RAD51 could be seen by western blot within 48 h in cells treated with the respective siRNA (Figures 1A–1C). We reasoned that the impact of protein depletion is translated into cellular phenotypes in the following 24 h and, hence, assessed HAC loss 72 h after siRNA treatment, measuring populations of cells with GFP fluorescence. In parallel, the division index (DI), an indicator of the number of cell divisions, was measured using a dye dilution assay (Figure S1A–S1C). Exposure to siBRCA2 and siPALB2 induced a small reduction in cell proliferation (DI = 3.16 and 3.25, respectively) compared to the negative control, siMisNeg (DI = 3.33), whereas siRAD51 treatment induced a higher reduction (DI = 2.80) (Figure 1D), showing that depletion of these factors delays cell cycle progression.

As expected, the rate of HAC loss in cells treated with siMisNeg was comparable to the natural HAC loss observed in cells cultured under blasticidin-free conditions (−Blast). Conversely, siRNA-mediated depletion of STK38, a serine/threonine kinase that is important for mitotic chromosome segregation, increased HAC loss, as shown previously.[20] Significantly, siBRCA2 treatment also resulted in an increase in HAC loss, albeit to a lesser degree compared to STK38 depletion (Figure 1E). In contrast, no detectable increase in HAC loss was found in cells treated with siPALB2 or siRAD51 (Figure 1E). We noted, however, that cells treated with siBRCA2 showed a modest but reproducible enrichment of mitotic cells after 72 h (Figure S1D). Given that neither GFP-Geminin nor GFP-CDT is expressed in late mitosis, even in the presence of the HAC construct, we considered that an apparent increase in GFP-negative cells could be caused by an enrichment in late mitosis rather than HAC loss. To test this possible artifact, we assessed the mitotic stage in siBRCA2-treated cells based on the chromatin morphology by DAPI staining (Figure S1E). Mitotic cells are detected by intense DAPI staining of chromatin, distinct from interphase cells, which display a rounded nuclear shape with less intense DAPI staining. Condensed chromosomes are visible from early mitosis and align to form metaphase plates before segregation into daughter cells in late mitosis. In BRCA2-depleted cells, the percentage of cells in mitosis almost doubled to 4.04% compared to the negative control (2.1%). However, the majority of these mitotic cells were found to be in early mitosis (3.43%) (Figure S1F). Thus, we concluded that the increased rate of HAC loss in siBRCA2-treated cells was unlikely to be related to prolonged mitosis.

It is possible that HAC integrates in endogenous chromosomes, affecting the readout of HAC loss based on GFP fluorescence. Hence, HAC loss was further evaluated by microscopy. We used fluorescence *in situ* hybridization (FISH) probes detecting tetO and CENP-B box sequences and determined the presence of HAC through the colocalization of both FISH signals in

CellPress
OPEN ACCESS

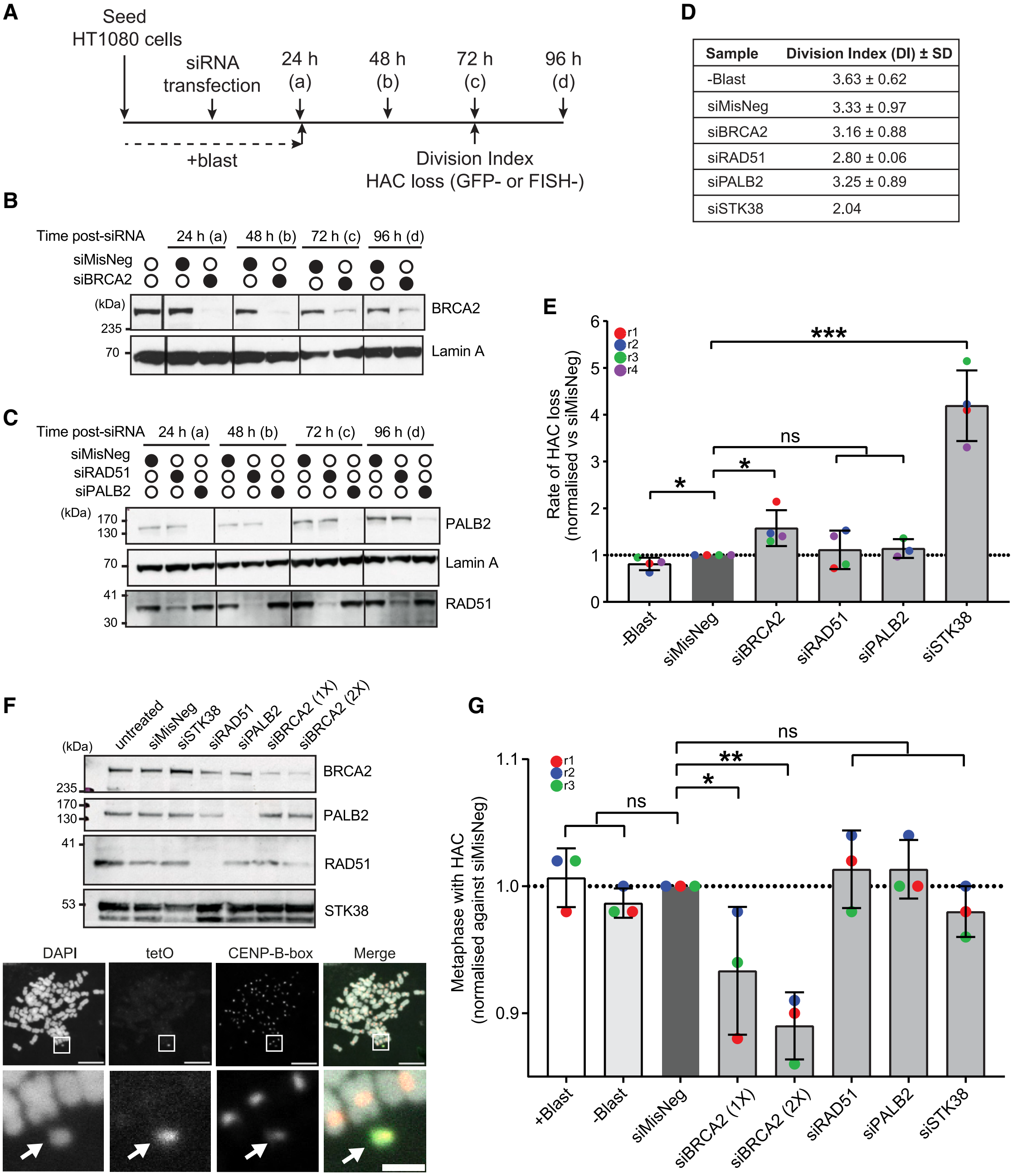

Sample	Division Index (DI) ± SD
-Blast	3.63 ± 0.62
siMisNeg	3.33 ± 0.97
siBRCA2	3.16 ± 0.88
siRAD51	2.80 ± 0.06
siPALB2	3.25 ± 0.89
siSTK38	2.04

Figure 1. BRCA2, but not PALB2 or RAD51, suppresses HAC loss in dividing cells

(A) Schematic of the HAC loss assay. (a)–(d) represent time points in (B) and (C).

(B and C) Western blot showing BRCA2, RAD51, and PALB2 siRNA depletion in HT1080 alphoid^{tetO} dGFP-HAC cells. Lamin A was used as a loading control.

(D) Division index (DI) after siRNA treatment. The DI was determined from a single biological experiment (three technical replicates for −Blast, siMisNeg, siBRCA2, siRAD51, and siPALB2 and one technical replicate for siSTK38). SD from three technical replicates.

(E) Rate of HAC loss after siRNA treatment. siSTK38 was included as a positive control. Error bars represent SD of at least three independent experiments. Unpaired t test. Data from each experiment were normalized to siMisNeg.

(F) Top: western blot showing siRNA-mediated protein depletion. siBRCA2 (1×) and siBRCA2 (2×) indicate 18 and 36 nM siRNA, respectively. Bottom: representative images of metaphase spread and FISH staining. Endogenous centromeres were detected with a CENP-B box probe (red in the merged image). HAC was detected with a tetO probe (green in the merged image, white arrow). The scale bar equals 10 μm. The inset scale bar equals 2 μm.

(G) Quantification of the three independent FISH experiments shown in (F). Unpaired t test. Data from each experiment were normalized to siMisNeg. 50 metaphase spreads were analysed per conditions for 3 independent experiments. Error bars represent SD. ns represents no statistical significance; $p < 0.05$ (*), $p < 0.01$ (**), $p < 0.001$ (***).

See also Figure S1.

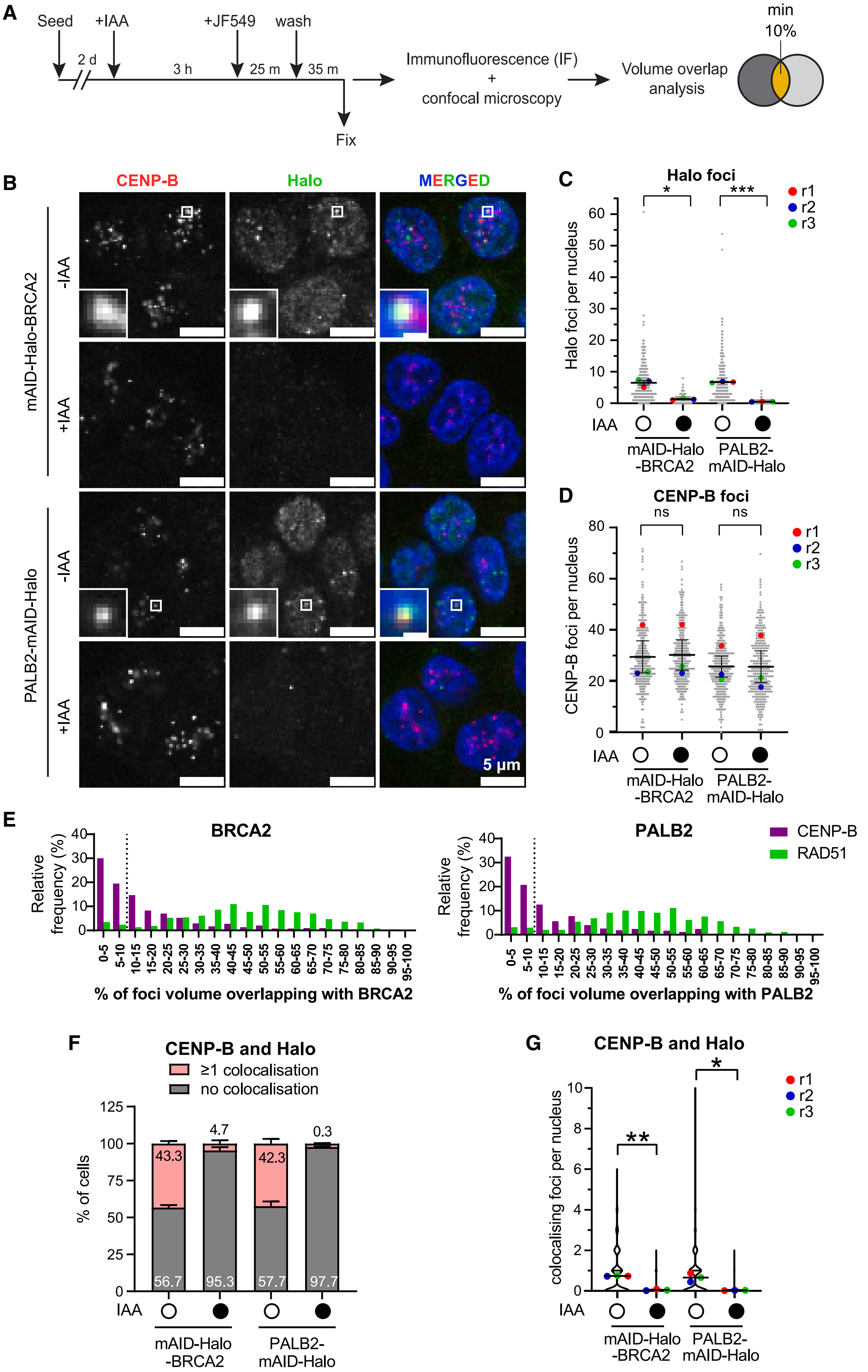

(legend on next page)

mitotically arrested individual cells (Figure 1F). Corroborating our flow cytometry-based HAC loss results, we observed a consistent decrease in HAC-containing cells upon siBRCA2 treatment (Figure 1G). Conversely, no difference was found in cells treated with siPALB2 or siRAD51. Of note, HAC loss was also not detected in mitotically arrested cells depleted of STK38, likely due to its multifaceted roles in early mitosis, which may have reduced the pool of metaphase cells available for FISH analysis. Overall, our results point to BRCA2, but not PALB2, as being important for the maintenance of HAC in HT1080 cancer cells.

BRCA2 and PALB2 depletion through the auxin-inducible degron system in HCT116 cells

Next, we sought to determine how BRCA2 and PALB2 impact endogenous chromosomes. To this end, we used chromosomally stable, near-diploid HCT116 colorectal carcinoma cell lines in which these factors could be conditionally depleted using the auxin-inducible degron (AID) system (Figure S2A).[26] We generated cell lines in which BRCA2 or PALB2 was endogenously tagged with the mini-AID epitope and the HaloTag in tandem (mAID-Halo) at the N terminus or C terminus, respectively (Figure S2B). By western blot analysis, we confirmed that these fusions, detected with an expected size shift of approximately 40 kDa, were depleted within 30 min of exposure to auxin (indole-3-acetic acid, IAA) (Figures S2C and S2D). Successful tagging of the endogenous gene was also validated by PCR (Figures S2E and S2F). Of note, while PCR of genomic DNA from cells harboring the PALB2-mAID-Halo fusion produced a DNA band at the expected size, a band smaller than the size of endogenous PALB2 was also detected (Figure S2E). Sequencing of this DNA band revealed the presence of an 11-nt deletion, leading to a 3-amino-acid truncation within the C terminus of PALB2 (Figure S2F). These C-terminal amino acids are predicted to interact with the N-terminal portion of the WD40 beta-propeller barrel structure, contributing to the overall structural stability of PALB2[27] (Figure S2G). Given that complete PALB2 loss is visible after IAA treatment (Figure S2D), we reasoned that this untagged and truncated PALB2 is unstable and non-functional. This was also supported with BRCA2 pull-down, where 2 h of IAA treatment in HCT116 PALB2-mAID-Halo cells completely abrogated its interaction with BRCA2 (Figure S2H). The expected impact of IAA-induced BRCA2 and PALB2 depletion was further confirmed by a significant decrease in cell survival after mitomycin C (MMC) treatment (Figures S3A–S3C) and by immunofluorescence (IF) staining, detecting S139 phosphorylated histone H2A.X (ɣ-H2A.X) and RAD51 foci as the surrogate of DNA damage signaling and HR repair, respectively (Figures S3D–S3J). Without IAA exposure and, hence, in the presence of the BRCA2 or PALB2 fusion, an MMC-dependent increase of RAD51 and ɣ-H2A.X foci was detected. Conversely, in cells exposed to IAA, we detected no increase in RAD51 foci in either cell line despite ɣ-H2A.X induction being unaffected. These observations demonstrate that the mAID-Halo-tagged BRCA2 and PALB2 are functional and that AID-induced depletion confers a significant deficiency of HR-mediated repair. Overall, these observations establish that the generated HCT116 mAID-Halo-BRCA2 and PALB2-Halo-mAID cell lines are valuable tools to investigate the function of BRCA2 and PALB2.

Imaging of BRCA2 and PALB2 using the HaloTag

We next set out to investigate the localization of BRCA2 and PALB2 in proximity to centromeres using the endogenously introduced HaloTag.[28–30] We visualized BRCA2 and PALB2 HaloTag fusion proteins using the Halo ligand Janelia Fluor 549 (JF549) (Figure 2A).

First, we validated our image analysis pipeline by assessing BRCA2 and PALB2 colocation with RAD51. Using 3D segmentation (Figure S4A), we found a similar number of BRCA2 (on average 6.7 foci) and PALB2 (on average 7.1 foci) Halo foci per nucleus, which significantly decreased upon 4 h of IAA exposure, as expected (Figures 2A, S4B, and S4C). RAD51 focus segmentation showed consistent numbers with our preceding measurements (Figures S3F, S3G, and S4D). We then quantified volume overlap-based colocalization to determine the percentage volume of a given RAD51 focus interacting with a BRCA2 or a PALB2 focus (Figure S4A). Under untreated conditions, we observed that the average overlap between RAD51 foci and BRCA2 or PALB2 foci was 47.2% and 44%, respectively (Figure S4E), indicating that colocalization corresponded to partial overlap in most cases. Based on the distribution of focus volume overlap, we chose to exclude interactions with less than 10% overlap for further analyses to avoid false positive events. Using this criterion, we found that, in the asynchronous interphase

Figure 2. BRCA2 and PALB2 localization at the centromeres

(A) Schematic of the experimental protocol for IAA depletion and staining using a Halo ligand (Janelia Fluor 549 [JF549]). Shown is the volume overlap analysis criterion.

(B) Representative images of mAID-Halo-BRCA2 and PALB2-mAID-Halo, visualized using JF549 Halo ligand, CENP-B by IF, and DNA stained by Hoechst. The scale bar equals 5 μm. The inset scale bar equals 1 μm.

(C) Number of Halo foci representing the fusion proteins detected using the 3D segmentation pipeline. Each datapoint represents one nucleus, and colored dots are means of the replicates. Paired t test. $n = 3$. Error bars represent SEM.

(D) Number of CENP-B foci detected using the 3D segmentation pipeline. Each datapoint represents one nucleus, and colored dots are means of the replicates. Error bars represent SEM. Paired t test. $n = 3$.

(E) Percentage of CENP-B or RAD51 focus volume overlapping with Halo-BRCA2 and PALB2-Halo foci. Values are the relative frequency of overlap from all colocalization events from the non-treated condition. RAD51 data are from Figure S4E.

(F) Percentage of nuclei with none or at least one colocalization event between CENP-B and Halo. 3 biological replicates with 100 cells analyzed in each condition per replicate. Error bars represent SEM.

(G) Number of colocalization events per nuclei. Colored dots represent the means of the replicates. Error bars represent SEM. Paired t test. ns represents no statistical significance; $p < 0.05$ (*), $p < 0.01$ (**), $p < 0.001$ (***).

See also Figures S2, S3, and S4.

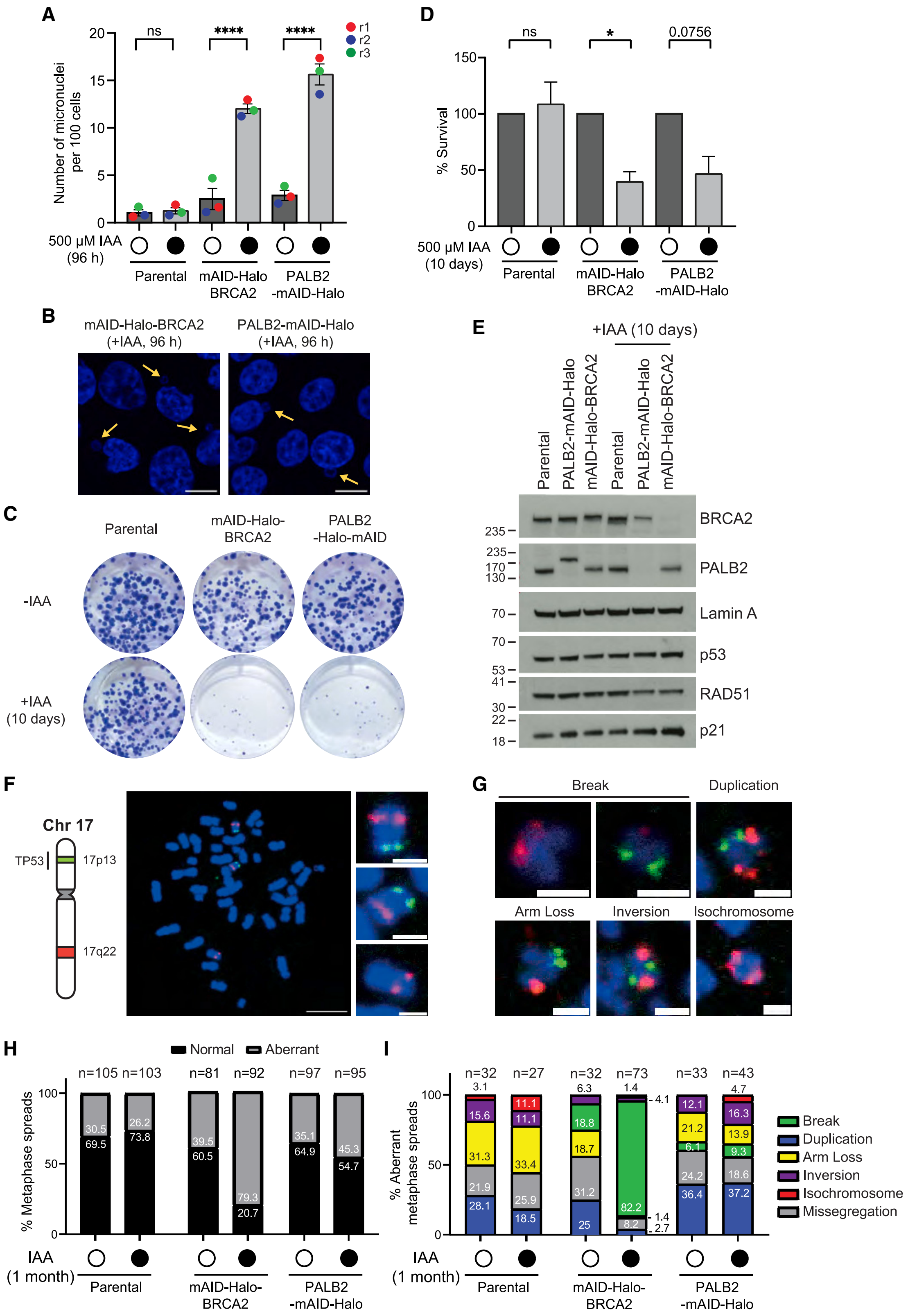

(legend on next page)

CellPress
OPEN ACCESS

population, 56.3% and 57.5% cells had at least one RAD51 focus colocalizing with a BRCA2 or PALB2 focus, respectively, with an average of two colocalizing foci per nucleus for both BRCA2 and PALB2 (Figure S4F). This is reduced to 0.1 and 0 events upon IAA treatment, consistent with the reduced numbers of RAD51 and Halo foci (Figure S4G). These results demonstrate that endogenous BRCA2 and PALB2 can be visualized confidently using the introduced HaloTag in our HCT116 *Os*TIR1 degron cells and through our colocalization pipeline.

To assess BRCA2 and PALB2 localization with centromeres, we combined Halo labeling and CENP-B IF staining (Figure 2B). Confirming our segmentation, BRCA2 and PALB2 Halo focus averages per cell were comparable to our analysis above, and CENP-B focus numbers were consistent across all conditions (Figures 2B–2D and S4C). We found that under IAA-untreated conditions, the average percentage of CENP-B focus volume that overlapped with BRCA2 foci (16%) and with PALB2 foci (16.5%) was lower than that observed for RAD51. This suggests that both BRCA2 and PALB2 can be located in close proximity to centromeres but not frequently in the core centromeric region (Figure 2E). Furthermore, we found that 43.3% and 42.3% of cells showed at least one colocalization event between CENP-B foci and BRCA2 or PALB2 foci, respectively (Figure 2F), with 0.7 colocalization events on average per nucleus (Figure 2G). IAA treatment significantly reduced colocalization events, consistent with the loss of the Halo foci under these conditions, while CENP-B remained unaffected. These results support the theory that both BRCA2 and PALB2 can associate with a subset of centromeres in interphase cells but that these events are rare and often found adjacent to but outside of the core centromere.

Long-term depletion of BRCA2 and PALB2 compromises chromosome stability through distinct mechanisms in HCT116 cells

While stable association of BRCA2 or PALB2 at core centromeres was infrequent, it was possible that their dynamic action at centromeres influenced CIN. Hence, we sought to further investigate the impact of BRCA2 and PALB2 depletion on chromosome stability in our HCT116 *Os*TIR1 degron cells. Our initial analysis of cells depleted of BRCA2 or PALB2 with IAA for 96 h demonstrated a substantial increase of micronucleus formation (Figures 3A and 3B). The depletion of BRCA2 or PALB2 for 10 days revealed an approximately 60% drop in cell survival compared to untreated samples by clonogenic survival assay (Figures 3C and 3D). Nonetheless, a proportion of cells was found to survive after 10 days of IAA exposure in the absence of detectable BRCA2 and PALB2 (Figure 3E), highlighting that depletion of BRCA2 or PALB2 triggers substantial cell stress that is successfully overcome in the surviving cells.

We hypothesized that this surviving population of cells may accumulate chromosomal aberrations a way that facilitates their continued survival, as seen in cancer development. Indeed, germline *BRCA2* and *PALB2* mutations and subsequent loss of function have been associated with several cancers, including medulloblastomas, where structural aberrations of chr17, which harbors TP53 in its p arms, have been reported.[31] Similar chr17 aberrations have also been described in colorectal cancers and gastro-esophageal adenocarcinomas.[32,33] Building on these notions and the principle that the HAC was designed based on centromeric α-satellite repeat sequences from chr17,[23] we examined whether and how BRCA2 and PALB2 depletion may impact chr17 structures. The three HCT116 *Os*TIR1 degron cell lines were cultured in the presence or absence of IAA for 1 month, and surviving cells were subjected to chr17 p- and q-arm FISH staining on metaphase spreads. As shown in Figure 3F, a normal HCT116 metaphase spread displayed the two copies of p and q arms with green and red staining, respectively. Corroborating the documented chr17 translocation (t(4;17)) in HCT116 cells,[34] we also observed an additional chromosome displaying a red q-arm signal only. Various chr17 aberrations were visible across all conditions with varying frequencies, including chromosome breaks, duplications, chromosome arm loss, inversions, and isochromosomes (Figure 3G).

Growth in IAA had little impact on HCT116 *Os*TIR1 parental cells in the percentage of metaphase spreads with aberrant chr17 FISH signal (Figure 3H). Markedly, BRCA2 depletion resulted in a 38.9% increase in chr17 aberrations, while PALB2 depletion increased the percentage of aberrant chr17 by 10.2%. As shown in Figure 3I, further classification of the types

Figure 3. Long-term depletion of BRCA2, but not PALB2, induces frequent chromosome breaks in HCT116 cells

(A) Number of micronuclei per 100 cells in HCT116 *Os*TIR1 degron cells after 500 μM IAA treatment for 96 h. At least 200 cells were analyzed per sample per replicate. $n = 3$. Error bars represent SEM from three biological replicates. Repeated measures (RM) one-way analysis of variance (ANOVA) with Tukey's multiple comparisons test.

(B) Representative images of micronuclei (yellow arrow). The scale bar equals 10 μm

(C) Representative images of colony growth in HCT116 *Os*TIR1 parental or mAID-degron cell lines with or without 500 μM IAA treatment for 10 days.

(D) Clonogenic cell survival of HCT116 *Os*TIR1 degron cells upon 500 μM IAA treatment. $n = 3$. Error bars represent SEM from three biological repeats. Paired t test.

(E) Western blot showing BRCA2 and PALB2 depletion after 10 days and 500 μM IAA treatment. Lamin A was used as a loading control.

(F) Representative image of HCT116 cell metaphase spread with chr17 p and q arms labeled in green and red, respectively. The scale bars equal 5 μm (left) and 2 μm (right).

(G) Representative images of different observed chr17 aberrations. The scale bar equals 2 μm. Isochromosome 17q (Iso(17q)) formation, a mirror-image chromosome consisting of the chr17 q arm, is specifically associated with a subset of medulloblastoma patients displaying BRCA2 and PALB2 mutations.[31]

(H) Quantification of metaphase spreads showing a normal vs. aberrant chr17 FISH probe signal after 1 month of 500 μM IAA treatment in HCT116 *Os*TIR1 degron cells.

(I) Quantification of types of chr17 aberrations appearing after 1 month of 500 μM IAA treatment in HCT116 *Os*TIR1 degron cells. Numbers represent percentage occurrence of the aberrant metaphase spreads shown in (H). At least 90 metaphase spreads were analyzed per condition from 3 independent repeats. Precise numbers are displayed above each sample. ns represents no statistical significance; $p < 0.05$ (*), $p < 0.01$ (**), $p < 0.001$ (***), $p < 0.0001$ (****).

See also Figures S2, S3, and S4.

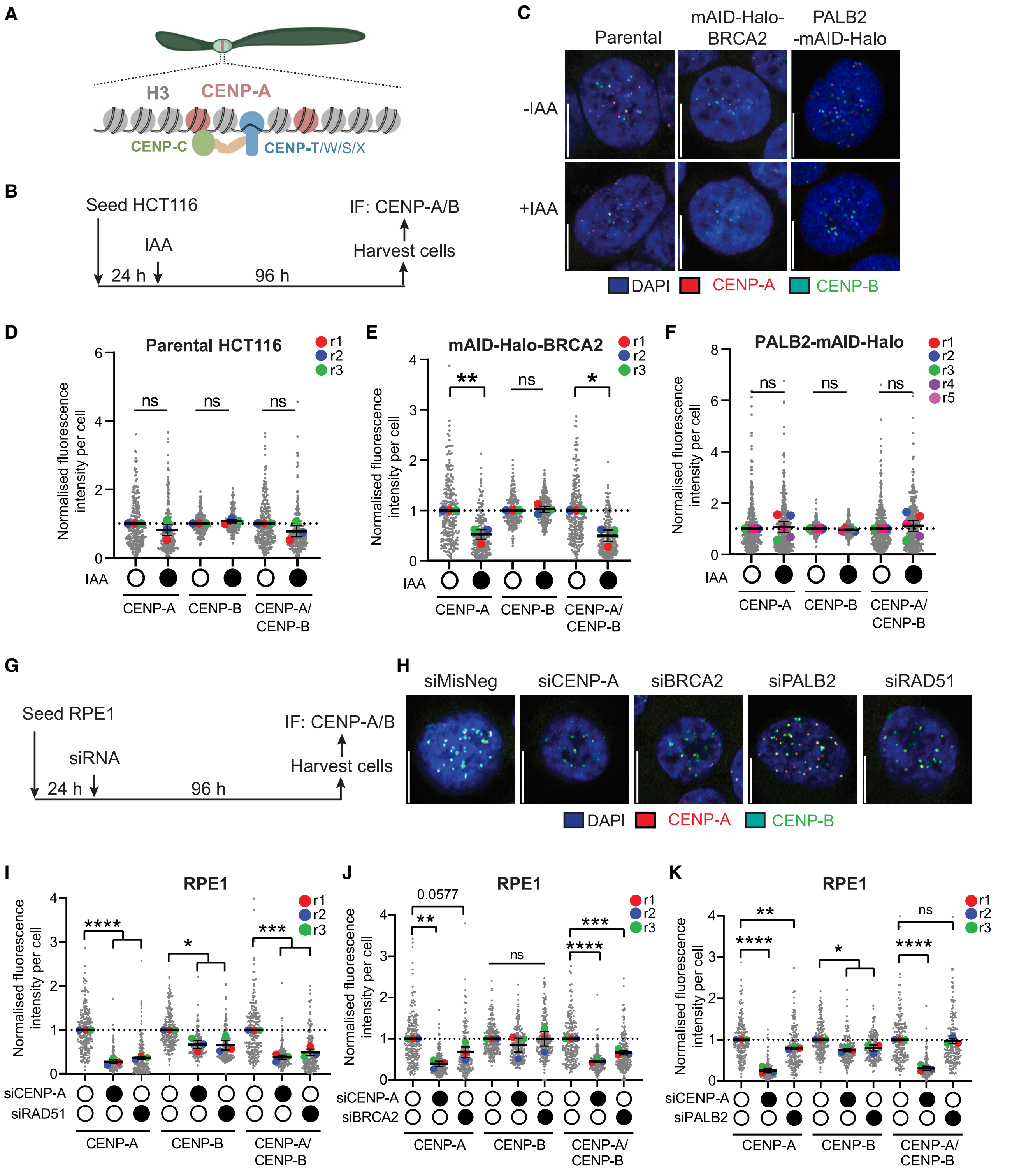

Figure 4. Depletion of BRCA2, but not PALB2, perturbs CENP-A occupancy at centromeres

(A) Schematic of centromere components.

(B) Schematic of the experimental protocol using 5 μM IAA.

(C) Representative images of CENP-A and CENP-B IF staining. The scale bar equals 10 μm

(D) CENP-A, CENP-B, or normalized CENP-A to CENP-B signal in HCT116 *Os*TIR1 parental cells.

(legend continued on next page)

of aberrations revealed minor increases in chr17 missegregation, arm loss, and isochromosome formation in IAA-treated HCT116 *Os*TIR1 parental cells, agreeing with the notion that HCT116 is largely chromosomally stable. In stark contrast, 82.2% of aberrations after BRCA2 depletion were chromosome breaks. Such an increase was not detectable upon PALB2 depletion, with small increases in chromosome breaks, inversions, and isochromosomes, whose composition was largely unchanged. Overall, examination of the long-term consequences of BRCA2 and PALB2 depletion revealed that PALB2 depletion has a modest effect on chr17 stability, while BRCA2 depletion correlates with a large increase in chr17 breaks and chromosome arm separation in HCT116 cells.

Loss of BRCA2, but not PALB2, impacts the occupancy of CENP-A at centromeres without affecting CENP-C or CENP-T

Cells with chromosomal breaks may gain a survival advantage in the absence of endogenous BRCA2, suggesting that the defects observed in IAA-treated mAID-Halo-BRCA2 cells could be attributed to a "survivorship bias." This led us to ask whether CENP-A, which binds to the centromere core and mediates its functionality (Figure 4A), was involved in the increased chromosome breakage seen in BRCA2-depleted cells. CENP-A is known to prevent centromere fragility[5,7] while also being recruited to DNA break sites.[35,36] Given our previous finding that RAD51 affects CENP-A occupancy at centromeres in both HCT116 and RPE1 cells,[37] we explored whether BRCA2 and PALB2 affect centromeric CENP-A.

We first examined asynchronous HCT116 *Os*TIR1 degron cells by IF, quantifying the intensity of CENP-A at CENP-B-defined centromeric regions (Figures 4B and 4C). In parental cells, no significant change of CENP-A or CENP-B signals was detected after 96 h of IAA exposure (Figure 4D). In contrast, BRCA2 depletion produced a significant reduction in CENP-A signal (Figure 4E). However, PALB2 depletion did not impact CENP-A levels at CENP-B-defined centromeres (Figure 4F). These differences are unlikely to be due to cell cycle effects, as IAA-induced BRCA2 and PALB2 depletion did not significantly impact cell cycle profiles compared to IAA-untreated controls (Figures S5A–S5C). Collectively, these data indicate that BRCA2, but not PALB2, impacts centromere epigenetics in HCT116 cells.

While the results for the degron cells were encouraging, we were concerned with the potential impact of IAA exposure on cellular growth and gene expression[38] as well as the widely recognized deficiency of MMR in HCT116 cells.[39] Indeed, factors involved in MMR have been shown to be enriched at centromeres and to be involved in centromere maintenance.[3,40] Hence, noncancerous RPE1 cells were used to validate our observations. Depletion of BRCA2 and PALB2 from RPE1 cells was confirmed after 72 h of siRNA treatment (Figures S5D–S5F). We also included siRAD51 treatment (Figure S5G), which led to a significant enrichment of G2/M phase cells at 34.4% compared to 7.19% in the control sample (Figures S5H and S5I). This agrees with previous findings that RAD51 loss impacts cell cycle progression, especially mitotic entry.[41] BRCA2 and PALB2 depletion both reduced the percentage of cells in G1 while marginally enriching S and G2/M populations (Figures S5H and S5I). Similar to HCT116 *Os*TIR1 degron cells, depletion of BRCA2 and PALB2 in RPE1 cells resulted in an increase in micronucleus formation; however, this was insignificant (Figure S5J).

siRNA-treated RPE1 cells were then assessed for CENP-A occupancy (Figures 4G and 4H). siRNAs targeting RAD51 and CENP-A, included as controls (Figures S5D and S5G), displayed a significant decrease in CENP-A signal per cell without affecting the CENP-B signal (Figure 4I), corroborating our previous observations.[37] Notably, there was a mild decrease in CENP-A levels in cells treated with siBRCA2 compared to siMisNeg controls (Figure 4J). This difference was more evident when the CENP-A signal was normalized against CENP-B. Intriguingly, PALB2 depletion led to a very modest but significant decrease in both CENP-A and CENP-B levels, but when the CENP-A signal was normalized to the CENP-B signal, the difference was lost (Figure 4K). These observations indicate that BRCA2 and, to a lesser extent PALB2, affect the key epigenetics of the centromere in RPE1 cells.

Notably, BRCA2 depletion had no detectable effect on the occupancy of the CCAN components CENP-C and CENP-T in HCT116 or RPE1 cells (Figure S6), suggesting that centromere function is largely maintained. Taken together, these observations suggest that BRCA2 affects the CENP-A-positive core region of centromeres but does not overlap with the CCAN-positive core regions.

BRCA2 and PALB2 impact centromeric DNA in a context-dependent manner

Given the intimate link between CENP-A and centromere fragility,[5,7] we next sought to determine whether the observed CENP-A occupancy could be explained by DNA breaks within centromeres. To this end, we used exo-FISH, a microscopy-based technique that has been developed to detect DNA strand breaks within repetitive regions of the genome, combining exonuclease III (ExoIII) treatment and FISH probes of interest (Figures 5A and S7A).[37,42] To assess DNA breaks at core

(E) CENP-A, CENP-B, or normalized CENP-A to CENP-B signal in HCT116 mAID-BRCA2 cells.

(F) CENP-A, CENP-B, or normalized CENP-A to CENP-B signal in HCT116 PALB2-mAID cells. All samples were normalized to the −IAA sample. Large colored dots represent experiment averages. At least 100 cells were analyzed per condition per repeat. Paired t test.

(G) Schematic of the treatment protocol.

(H) Representative images after 96 h of siRNA treatment in asynchronous RPE1 cells. The scale bar equals 10 μm

(I–K) Quantification of the CENP-A focus signal, CENP-B focus signal, and normalized CENP-A/CENP-B signal for CENP-A-depleted, BRCA2-depleted (I), and PALB2-depleted (J) RPE1 cells. For each measurement, the signal was normalized to the siMisNeg control. $n = 3$. At least 100 cells were analyzed per condition per repeat. Large colored dots represent the mean of each repeat. Error bars represent SEM. Paired t test. ns represents no statistical significance; $p < 0.05$ (*), $p < 0.01$ (**), $p < 0.001$ (***), $p < 0.0001$ (****).

See also Figures S5 and S6.

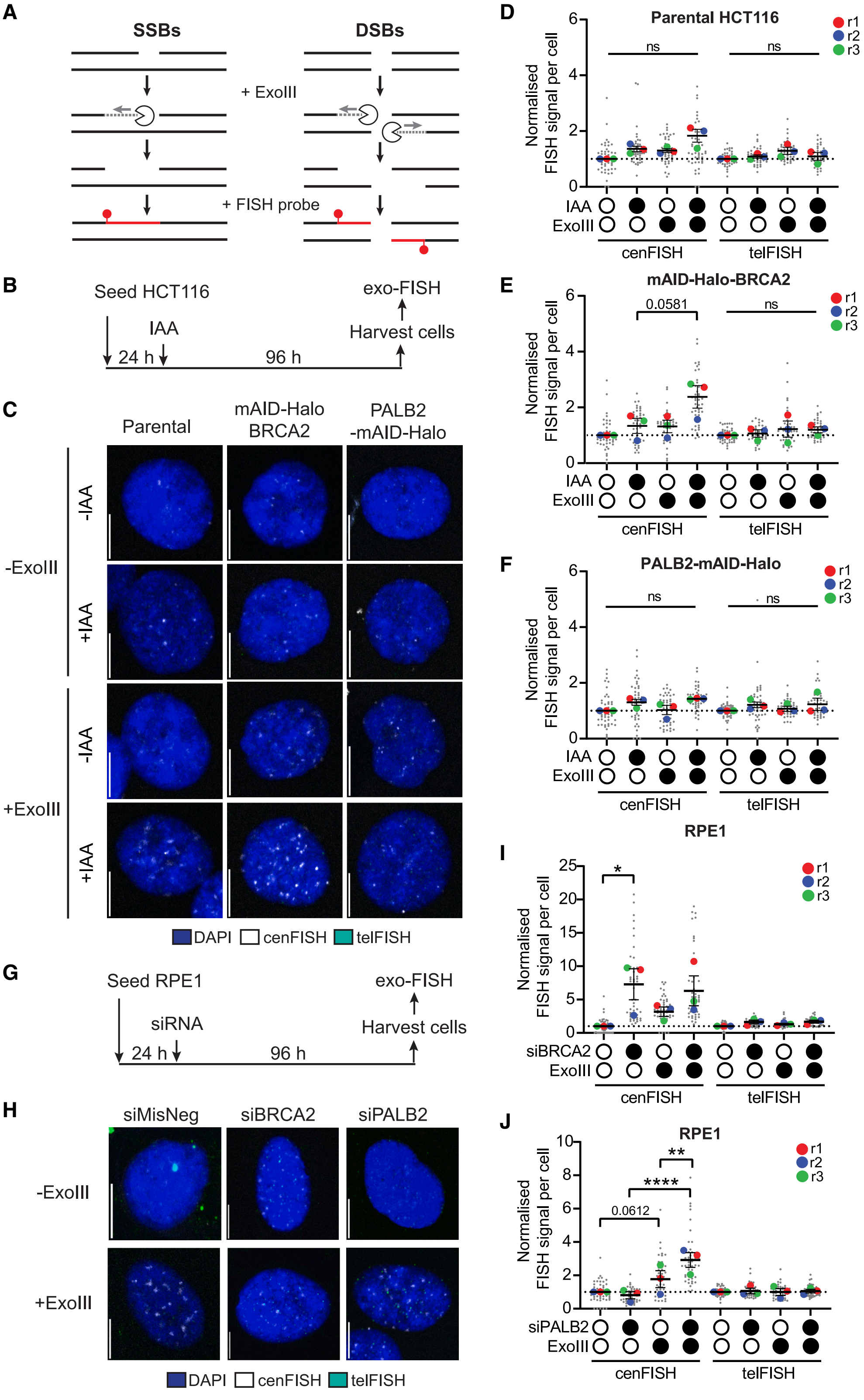

(legend on next page)

centromeres, we used FISH probes complementary to the CENP-B box, present throughout the centromeric α-satellite repeats (cenFISH).

In HCT116 *Os*TIR1 parental cells, 96 h of IAA exposure elicited an insignificant increase in cenFISH signal irrespective of ExoIII treatment (Figures 5B–5D). mAID-Halo-BRCA2 cells showed a strong increase in ExoIII-dependent cenFISH signal with a near-statistical significance under IAA-treated conditions (Figure 5E). Conversely, PALB2-Halo-mAID cells were largely unresponsive to ExoIII treatment, and PALB2 degradation did not significantly alter cenFISH signal intensity compared to the control (Figure 5F). Collectively, these observations suggest a role of BRCA2, but not PALB2, in limiting centromeric DNA breaks in HCT116 cells.

RPE1 cells treated with siRNA were also assessed by exo-FISH (Figures 5G and 5H). Corroborating previous observations,[37] ExoIII treatment of siMisNeg control samples increased the cenFISH signal, representing spontaneous DNA breaks that arise at the centromere.[37] BRCA2 depletion resulted in a significant increase in cenFISH signal without ExoIII treatment, with no further increase upon ExoIII treatment (Figure 5I), suggesting greater exposure of unbroken single-stranded DNA (ssDNA). Conversely, PALB2 depletion made no significant difference in cenFISH signal without ExoIII treatment, whereas ExoIII treatment conferred a significant increase in cenFISH signal, indicating an increase of centromeric DNA breaks (Figure 5J). Collectively, these findings indicate that, in RPE1 cells, BRCA2 suppresses the amount of exposed centromeric ssDNAs without DNA breaks, which may arise from secondary DNA structure formation (Figure S7B). PALB2, on the other hand, limits the accumulation of spontaneous centromeric DNA breaks.

MLH1 is responsible for centromere fragility in the absence of BRCA2 or PALB2

Although the HCT116 cell line is chromosomally stable, it exhibits a high mutation rate and the ability to rapidly evolve its genome to support its survival,[43] often explained by biallelic deletion in the Mut L homolog 1 (*MLH1*) gene, which encodes a key initiator of MMR.[39,44,45] Notably, centromeres have been described as some of the most rapidly evolving regions of the genome, with significant differences in their DNA sequence and size while maintaining their functionality.[46,47] This phenotype has been proposed to be driven by HR between centromere repeats.[37] Furthermore, intimate links between MMR and HR have been recognized. For example, MMR suppresses HR between sequences with mismatches in normally dividing somatic cells,[48] whereas the simultaneous downregulation of HR and MMR elicits cell death[49] or the adaptive mutability that drives malignancy.[50] Since MMR factors are also known to be enriched at centromeres,[3] we postulated that the observed difference in exo-FISH analyses between HCT116 and RPE1 cells could be attributed to their differing MMR status. To test this idea, we generated clonal MLH1 knockout (KO) RPE1 cell lines by CRISPR-Cas9 (Figure 6A) and investigated the impact of BRCA2 or PALB2 depletion. In agreement with a previous study,[49] depletion of BRCA2 or PALB2 in RPE1 MLH1 KO cells reduced overall cell survival (Figures 6B–6D), demonstrating that MLH1 and BRCA2/PALB2 synergistically support cell survival. To our surprise, we observed no detectable increase in cenFISH signals with or without ExoIII treatment upon depletion of BRCA2 or PALB2 in RPE1 MLH1 KO cells (Figures 6E–6G). These observations suggest that MLH1 promotes centromere fragility and that BRCA2 and PALB2 counterbalance this effects in RPE1 cells.

BRCA2 suppresses unsolicited centromeric transcription in non-cancerous cells

Intriguingly, our exo-FISH analysis of cells arrested in early mitosis by an Eg5 inhibitor, S-trityl-L-cysteine (STLC),[51] showed no detectable impact of BRCA2 or PALB2 depletion on the level of centromere breaks in HCT116 (Figures S7C–S7G) or RPE1 cells (Figures S7H-S7J). This observation led us to consider the role of BRCA2 and PALB2 outside of S, G2, and early mitosis, as replicative stress could otherwise lead to premature mitotic entry with under-replication, followed by DNA breaks in early mitosis, triggering mitotic DNA synthesis, known as MiDAS.[52] This notion is also supported by the previous finding that BRCA2 deficiency affects early-replicating regions rather than late-replicating regions such as centromeres,[53] which subsequently undergo MiDAS.[54]

Importantly, during late M and early G1, centromeres are actively transcribed by RNA polymerase II (RNAPII), assisting the incorporation of CENP-A and centromeric chromatin

Figure 5. Depletion of BRCA2 and PALB2 induces aberrant DNA structures in a context-dependent manner

(A) Schematic of the exo-FISH principle. Single strand breaks (SSBs) and DSBs generated *in vivo* are digested by exonuclease III (ExoIII) *in vitro*, exposing ssDNA, which can be detected using centromere-specific (cenFISH) or telomere-specific (telFISH) FISH probes by microscopy.

(B) Schematic of the experimental protocol using 5 μM IAA.

(C) Representative images of exo-FISH. The scale bar equals 10 μm

(D) exo-FISH in HCT116 *Os*TIR1 parental cells with or without 5 μM IAA for 96 h

(E) exo-FISH in HCT116 mAID-BRCA2 cells with or without 5 μM IAA for 96 h

(F) exo-FISH in HCT116 mAID-PALB2 cells with or without 5 μM IAA for 96 h The cenFISH and telFISH signals were normalized to −IAA- and −ExoIII-treated samples. Large colored dots represent the average of each independent experiment. $n = 3$. At least 15 cells were analyzed per condition per repeat. Error bars represent SEM. RM one-way ANOVA with Tukey's multiple-comparisons test.

(G) Schematic of the siRNA treatment protocol for exo-FISH.

(H) Representative images of exo-FISH after 96 h of siRNA treatment. The scale bar equals 10 μm

(I and J) Graphs showing the impact of HR factor depletion BRCA2 (I) and PALB2 (J) on the exo-FISH signal. Samples were normalized to the MisNeg cenFISH or telFISH signal without ExoIII treatment. Large colored datapoints represent the averages of each independent repeat. $n = 3$. At least 15 cells were analyzed per condition per repeat. Error bars represent SEM. RM one-way ANOVA with uncorrected Fisher's least signifcant difference (LSD). ns represents no statistical significance; $p < 0.05$ (*), $p < 0.01$ (**), $p < 0.001$ (***).

See also Figures S5 and S7.

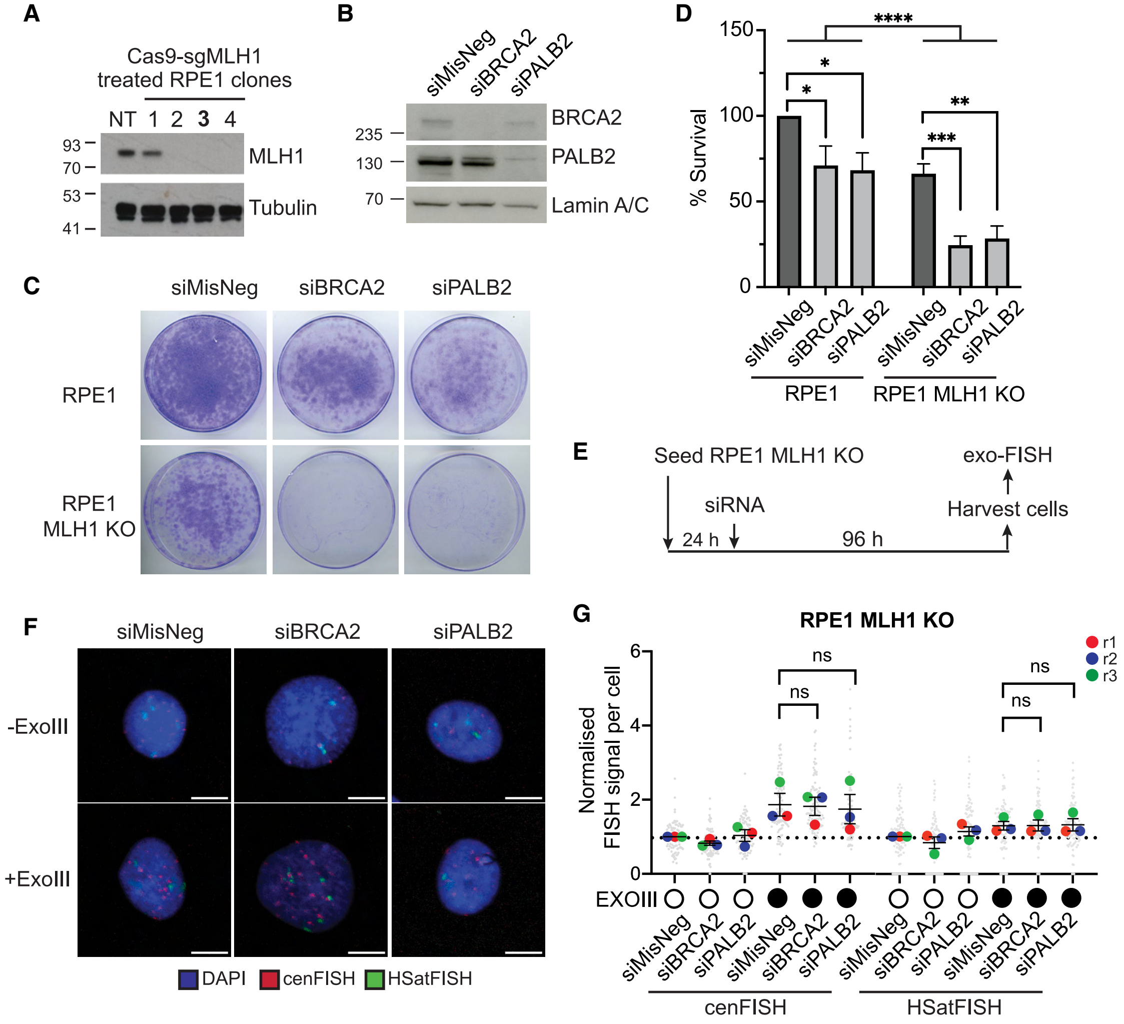

Figure 6. MLH1 depletion neutralizes aberrant centromere structures in BRCA2- and PALB2-depleted RPE1 cells

(A) Western blot showing MLH1 expression in RPE1 MLH1 KO clones generated using Cas9-sgMLH1. Tubulin was used as a loading control. Clone 3 (bold) was used for further study.

(B) Western blot showing 72 h of siRNA-mediated depletion of BRCA2 and PALB2 in RPE1 MLH1 KO cells. Lamin A was used as a loading control.

(C) Representative images of colony growth in siRNA-depleted RPE1 parental cells and MLH1 KO cells.

(D) The percentage of cell viability in RPE1 parental cells and MLH1 KO cells after 96 h of siRNA-induced BRCA2 or PALB2 depletion. Data represent an average of three independent experiments, and error bars represent SD. Two-tailed t test.

(E) Schematic of siRNA depletion of RPE1 MLH1 KO cells for exo-FISH.

(F) Representative images of exo-FISH of RPE1 MLH1 KO cells after 96 h of siRNA-mediated depletion of BRCA2 or PALB2.

(G) exo-FISH of RPE1 MLH1 KO cells with or without siRNA-mediated depletion of BRCA2 or PALB2. Samples were normalized to the siMisNeg cenFISH or human satellite II+III FISH (HSatFISH) signal without ExoIII treatment. Each datapoint represents one nucleus, and large colored datapoints represent the averages of each independent repeat. $n = 3$. At least 25 cells were analyzed per condition per repeat. Error bars represent SEM. RM one-way ANOVA with Tukey's multiple-comparisons test. ns represents no statistical significance; $p < 0.05$ (*), $p < 0.01$ (**), $p < 0.001$ (***).

organization[55–57] (Figure 7A). In light of our earlier finding that BRCA2, PALB2, and RAD51 form a steady-state complex with a chromodomain-containing nuclear protein, MRG15,[58] which recognizes the epigenetic marker trimethylated lysine 36 in histone H3 (H3K36me3), a histone modification associated with active transcription, we wondered whether BRCA2 or PALB2 affects centromeric transcription, potentially explaining the defective CENP-A incorporation and open structures at centromeres in BRCA2-deficient cells, as shown in Figures 4 and 5, respectively. Strikingly, our RT-qPCR analysis revealed a significant increase in centromeric transcripts in BRCA2-depleted RPE1 cells (Figures 7B and 7C). A modest but not statistically significant increase in centromere transcripts was also found in RAD51-and PALB2-depleted RPE1 cells. By contrast, such an effect was not detected in HCT116 cells depleted of BRCA2 or PALB2 (Figure 7D). Taken together, our results demonstrate that BRCA2 plays a key role in counteracting transcriptional overdrive at centromeres in RPE1 cells but not in HCT116 cells.

CellPress
OPEN ACCESS

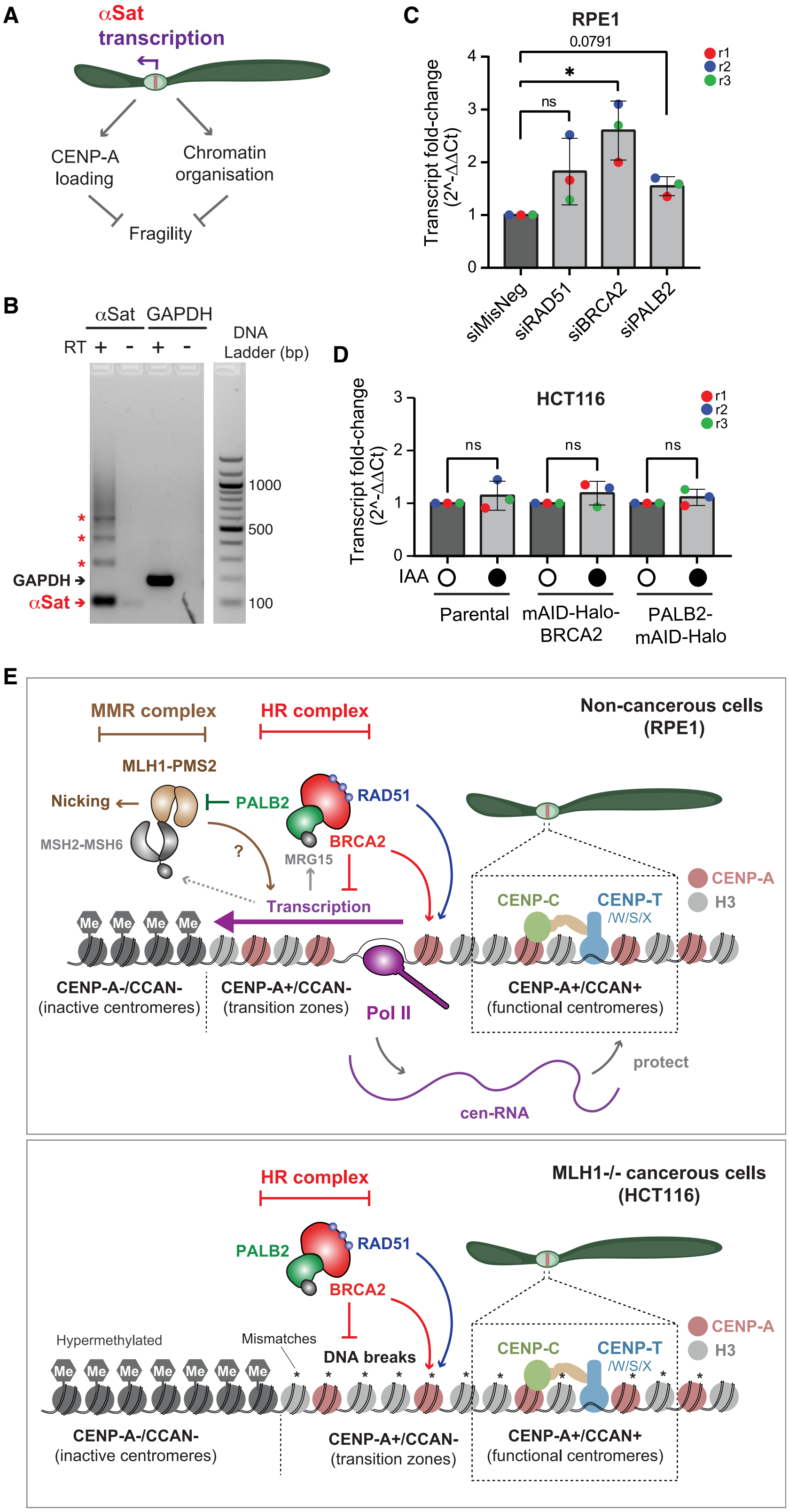

Figure 7. BRCA2 depletion specifically increases centromere transcription in RPE1 cells

(A) Schematic of centromere transcription and its biological impact.

(B) qPCR amplicons on 2% agarose gel at the expected sizes using α-satellite or GAPDH primer pairs in both the reverse-transcribed sample and the non-reverse-transcribed control.

(C) Mean RT-qPCR results from RPE1 cells. Three independent experiments. Shapiro-Wilks test for normality followed by ordinary one-way ANOVA of mean cycle threshold (Ct) values with Brown-Forsythe test of variances and Dunnett's multiple-comparisons test. Error bars represent SD.

(D) RT-qPCR results from HCT116 *Os*TIR1 cells with and without depletion of BRCA2 or PALB2 by 5 μM IAA exposure for 96 h. Data presented are means from three independent experiments. Shapiro-Wilks test for normality followed by ordinary one-way ANOVA of mean Ct values with Brown-Forsythe test of variances and Šídák's multiple-comparisons test. Error bars represent SD. ns represents no statistical significance; $p < 0.05$ (*).

(E) Top: the model for BRCA2 and PALB2 roles in centromere maintenance in non-cancerous RPE1 cells. BRCA2 prevents the transcriptional overdrive at centromeres and ensures CENP-A association. Centromeric transcription is also monitored by MSH2-MSH6 (MutS), triggering PMS2-MLH1 (MutL)-mediated DNA incision in the absence of PALB2. BRCA2 depletion leads to increased centromere transcripts, a process dependent on MLH1. In the absence of MLH1, neither transcriptional increase nor nicking occurs, though this compromises the overall fitness of centromeres and cell survival. Bottom: the model for BRCA2 and PALB2 functions at centromeres in cancerous MLH1-deficient HCT116 cells. In the absence of MLH1, uncorrected mismatches may disrupt centromeric transcription, while BRCA2 independently safeguards centromeric DNA from breakage through mechanisms that remain unidentified.

DISCUSSION

This study provides evidence that BRCA2 and, to a lesser extent, PALB2 act on centromeres to protect their integrity. The impact of BRCA2 is evident from our multifaceted analyses detecting HAC loss, CENP-A occupancy, centromere fragility, and centromeric transcripts. The impact of PALB2 depletion is limited in non-cancerous RPE1 cells with increased centromeric DNA breaks, which are concealed in the absence of MLH1, a key effector of MMR.

Our results are best explained by the model shown in Figure 7E. BRCA2 and PALB2 dynamically associate with centromeres undergoing active transcription. In non-cancerous cells, BRCA2 offsets transcriptional overdrive, which might otherwise destabilize CENP-A, leading to its eviction. In the absence of BRCA2, MLH1 exacerbates centromere fragility with bubble structures, which may be associated with enhanced centromeric transcription; for example, by correcting mismatches and preventing RNAPII stalling. The resulting centromeric transcripts could, however, protect the core centromeres by stabilizing CCAN,[55] thereby enhancing resilience against CIN and supporting overall cell survival. PALB2, on the other hand, may prevent unwanted nicking of centromeric DNA by counteracting MLH1 without significantly impacting CENP-A occupancy. Overall, our model proposes a balancing act of factors involved in HR (BRCA2 and PALB2) and MMR (MLH1) in fine-tuning centromeric transcription, which has a double-edged impact in regulating CENP-A occupancy and centromere integrity. MMR at centromeres may also prevent unwanted recombination between centromeric repeats.[48] In cancerous cells, however, there may be additional sources of centromeric fragility, independent of MMR factors, that are primarily safeguarded by BRCA2. This may also explain the increase in HAC loss in BRCA2-depleted HT1080 cells, which is cancer derived with no known defects in MMR.

This study advances the understanding of CIN in BRCA2-defective cancer cells, including those with abnormal chromosome numbers.[15,59] While the role of BRCA2 in mitosis has drawn interest in recent years, it remains contentious whether these mitotic phenotypes reflect its canonical roles with PALB2 and RAD51 in repairing or protecting centromeric DNA or whether it has centromere-specific or other mitotic functions. For example, a direct role of BRCA2 at centromeres to activate the spindle assembly checkpoint and facilitate metaphase chromosome alignment has been proposed.[59,60] Also, a role of BRCA2 in cytokinesis has been shown,[61,62] though this was then contested in a separate study.[63] Here, using the HAC system, we demonstrated that depletion of BRCA2, but not PALB2, causes CIN in HT1080 cells (Figure 1). Unexpectedly, RAD51 depletion elicited no detectable increase in HAC loss, contrary to its well-described role in genome stability as an effector of BRCA2. While this observation requires further validation (see Limitations of the study), RAD51 may indeed play an insignificant role in chromosome transmission in dividing cells, as indicated in assays that monitor HAC transmission at each cell division (Figure 1E), distinct from RAD51's role in repairing centromeric repeat arrays in non-dividing quiescent cells.[37]

Our analysis using AID-induced depletion of BRCA2 or PALB2 in HCT116 cells revealed striking differences in chromosomal phenotypes despite both proteins partially localizing at centromeres and affecting cell survival to a similar extent. Long-term BRCA2 depletion elicited breaks between p and q arms of chr17, unlike PALB2 (Figure 3). HCT116 cells and other colorectal cancer cell lines contain several common breakpoints within chr17, including the centromere and pericentromere.[34] Significantly, analysis of 345 cases of adenocarcinoma of the large intestine[64] revealed that 50% of the 50 most common recurrent breakpoints were located within centromeric regions, with chr17 being the most frequent.[34] Furthermore, analysis of genome-wide ligation of 3′-hydroxy ends followed by sequencing (GLOE-seq) and end sequencing (END-seq) datasets from HCT116 cells also uncovered an enrichment of spontaneous DNA breaks at the centromere.[37,65–67] However, neither of these observations (i.e., HAC loss or chr17 breakage) definitively reveals the direct impact of BRCA2 or PALB2 on centromeres.

This study, using exo-FISH, provides high-resolution insight into how BRCA2 might influence endogenous centromeres. BRCA2 depletion in HCT116 cells conferred an accumulation of DNA breaks (Figure 5E), whereas in RPE1 cells, we observed aberrant centromeric DNA with no clear evidence of DNA breaks (Figure 5I), most plausibly reflecting the accumulation of non-B DNA structures such as bubbles (Figure S7B). Conversely, PALB2 depletion conferred no increase in DNA breaks in HCT116 cells (Figure 5F), but a clear increase in centromeric DNA breaks was detected in RPE1 cells (Figure 5J). Our further analysis using MLH1 KO RPE1 cells indicated that this difference may be partly due to MLH1, which is defective in HCT116.[39] Canonically, MLH1 forms a complex with the endonuclease PMS2, recruited to mismatched DNA recognized by the MSH2-MSH3/6 (MutS homolog) complex, and initiates MMR through an incision. Notably, the MMR machinery has been also shown to bind centromeric DNA hairpin structures.[3,68] It is tempting to speculate that, in BRCA2-depleted RPE1 cells, increased transcriptional activity at centromeres may contribute to aberrant secondary DNA structures, which may be protected by MSH2-MSH3/6 without the induction of MLH1-PMS2-mediated DNA breaks. Accumulated centromeric transcripts may then safeguard the CCAN-bound core centromeres,[69] ensuring centromere functionality.

The study further revealed a previously unrecognized role of PALB2 at centromeres. Our data suggest that PALB2 protects centromeric DNA against MLH1/PMS2-mediated incision. Notably, MSH6 has been shown to facilitate MMR by interacting with H3K36me3 at transcriptionally active regions via its proline-tryptophan-tryptophan-proline (PWWP) domain.[70,71] PALB2 similarly associates with actively transcribed genes through its interaction with MRG15,[58] whose chromodomain binds H3K36me3 through a bipartite binding mechanism reminiscent of MSH6's PWWP domain.[72] Therefore, we envision that MRG15-PALB2 competes with the MMR machinery at transcribed chromatin, such as centromeres, and counteracts the unwanted activation of the MLH1/PMS2 endonuclease complex. Alternatively, it is plausible that PALB2 may promote DNA methylation by DNA methyltransferases, as has been reported

CellPress
OPEN ACCESS

for BRCA1,[73] another factor that is often described to act with PALB2. In this scenario, PALB2 depletion may lead to hypomethylation, rendering the centromere more sensitive to MLH1/PMS2-mediated incision in RPE1 cells. Finally, it is also conceivable that PALB2 acts at highly methylated centromeric regions that are not bound by CENP-A. Indeed, an analogous role of BRCA1 in maintaining heterochromatin domains flanking the kinetochore has been reported.[74] One possible scenario is that BRCA1 and PALB2 act at heterochromatic regions to maintain their structural integrity, independent from BRCA2 and RAD51. Consistent with this notion, a role of PALB2 in centromere structure is also suggested by our observation that both CENP-A and CENP-B occupancy is reduced in PALB2-depleted RPE1 cells (Figure 4K). Regardless, the relative occupancy of CENP-A compared to CENP-B was maintained under PALB2-depleted conditions, indicating that centromeric DNA breaks are not sufficient to destabilize CENP-A. The functional difference between these factors at centromeres warrants further investigation.

Limitations of the study

This study uncovers a previously unrecognized role of BRCA2 and PALB2 at centromeres but possesses some limitations. First, we acknowledge that the HAC loss assay has a previously recognized limitation in validating essential proteins with well-known centromeric function, such as CENP-A, due to a high level of cytotoxicity.[20] Indeed, increased cell toxicity is evident upon RAD51 depletion with reduced cell division, potentially masking the extent of HAC loss. While the number of cell divisions was considered when normalizing the rate of HAC loss, cells arrested in S/G2 phase may have negatively affected the readout. Second, the causal relationship between CENP-A and centromere fragility remains unresolved; it is unclear whether reduced CENP-A leads to centromere fragility or whether the fragility itself results in CENP-A loss. Third, while the exo-FISH method is highly sensitive, it provides an indirect measure of centromeric DNA breaks and structures, leaving the precise location, number, and nature of these fragilities uncertain. Finally, other factors contributing to centromere breaks in the absence of BRCA2 in MLH1-defective HCT116 cells have yet to be identified. Future research should focus on elucidating the molecular mechanisms through which BRCA2 functions at centromeres and determining how these mechanisms vary across different cell lines. This will provide exciting insight into potential cancer therapy strategies.

RESOURCE AVAILABILITY

Lead contact

Requests for further information, resources, and reagents should be directed to and will be fulfilled by the lead contact, Fumiko Esashi (fumiko.esashi@path.ox.ac.uk).

Materials availability

Plasmids and cell lines generated in this study are available upon request.

Data and code availability

- The source data generated and/or analyzed in this study are included in the manuscript or have been deposited at Mendeley Data and are publicly available as of the date of publication. Accession numbers are listed in the key resources table.
- This paper does not contain original code.
- Any additional information required to reanalyze the data reported in this paper is available from the lead contact upon request.

ACKNOWLEDGMENTS

We thank Profs. William C. Earnshaw, Lars Jansen, Jessica Down, Ulrike Gruneberg, Nicholas Lakin, and Jordan Raff for insightful discussions. We also thank Alan Wainman, Robert Hedley, and Michal Maj and members of Genome Engineering Oxford for their invaluable help with microscopy, flow cytometry, and CRISPR gene editing. We also thank Mrs. Chris Raff for assisting with the generation of mAID-Halo knockin constructs and HCT116 cell lines and Drs. Adele Alagia and Santosh Kumar for their assistance in establishing the RT-qPCR method to detect centromere transcripts. This work was supported by the Medical Research Council (MR/W017601 to F.E.) and the Biotechnology and Biosciences Research Council (BB/Y512928/1 to F.E.). F.E. was supported by the Wellcome Trust Senior Research Fellowships in Basic Biomedical Science (101009/Z/13/Z) and is thankful for support from the Edward Penley Abraham Research Fund. E.G. was a recipient of the Medical Sciences Graduate School studentship, funded by the Medical Research Council (18/19_MSD_2111222). L.R. was a recipient of the Oxford Interdisciplinary Bioscience Doctoral Training Partnership, sponsored by the Biotechnology and Biosciences Research Council (BB/M011224/1, Project 1757783). C.W.B.L. is a recipient of the Oxford-Dr HY Mok Graduate Scholarship (SFF2122-HYM-1417882). M.T.K. was supported by JSPS KAKENHI grants (JP21H04719 and JP23H04925) and a JST CREST grant (JPMJCR21E6). R.G. is supported by The Scientific and Technological Research Council of Türkiye (TUBITAK) (grant 1059B192202201) 2219 Post-Doctoral Research Fellowship Program.

AUTHOR CONTRIBUTIONS

F.E. conceived and supervised the project with help from H.M., V.L., and N.K. E.G. and C.W.B.L. performed experiments using HCT116 and RPE1 cells with help from J.W. and X.S. L.R. planned and conducted the HAC loss assay with help from M.L. and N.G. under the supervision of N.K. and V.L. E.Z.G. conducted imaging of Halo fusions in HCT116 cells and co-localization analyses. J.W. generated RPE1 MLH1 KO cells and assessed centromere transcripts with help from E.G. R.G. conducted the survival assay of RPE1 MLH1 KO cells. M.T.K. assisted with generating HCT116 mAID cell lines. E.G., L.R., C.W.B.L., J.W., E.Z.G., and F.E. evaluated and curated data. F.E., E.G., and L.R. wrote the original manuscript with help from all co-authors.

DECLARATION OF INTERESTS

The authors declare no competing interests.

STAR★METHODS

Detailed methods are provided in the online version of this paper and include the following:

- KEY RESOURCES TABLE
- EXPERIMENTAL MODEL AND STUDY PARTICIPANT DETAILS
  - Cell lines
- METHOD DETAILS
  - Materials and methods
- QUANTIFICATION AND STATISTICAL ANALYSIS

SUPPLEMENTAL INFORMATION

Supplemental information can be found online at https://doi.org/10.1016/j.celrep.2025.115259.

Received: November 25, 2023
Revised: September 6, 2024
Accepted: January 13, 2025
Published: January 31, 2025

REFERENCES

1. McKinley, K.L., and Cheeseman, I.M. (2016). The molecular basis for centromere identity and function. Nat. Rev. Mol. Cell Biol. *17*, 16–29. https://doi.org/10.1038/nrm.2015.5.
2. Erliandri, I., Fu, H., Nakano, M., Kim, J.H., Miga, K.H., Liskovykh, M., Earnshaw, W.C., Masumoto, H., Kouprina, N., Aladjem, M.I., and Larionov, V. (2014). Replication of alpha-satellite DNA arrays in endogenous human centromeric regions and in human artificial chromosome. Nucleic Acids Res. *42*, 11502–11516. https://doi.org/10.1093/nar/gku835.
3. Aze, A., Sannino, V., Soffientini, P., Bachi, A., and Costanzo, V. (2016). Centromeric DNA replication reconstitution reveals DNA loops and ATR checkpoint suppression. Nat. Cell Biol. *18*, 684–691. https://doi.org/10.1038/ncb3344.
4. Crosetto, N., Mitra, A., Silva, M.J., Bienko, M., Dojer, N., Wang, Q., Karaca, E., Chiarle, R., Skrzypczak, M., Ginalski, K., et al. (2013). Nucleotide-resolution DNA double-strand break mapping by next-generation sequencing. Nat. Methods *10*, 361–365. https://doi.org/10.1038/nmeth.2408.
5. Giunta, S., and Funabiki, H. (2017). Integrity of the human centromere DNA repeats is protected by CENP-A, CENP-C, and CENP-T. Proc. Natl. Acad. Sci. USA *114*, 1928–1933. https://doi.org/10.1073/pnas.1615133114.
6. Jaco, I., Canela, A., Vera, E., and Blasco, M.A. (2008). Centromere mitotic recombination in mammalian cells. J. Cell Biol. *181*, 885–892. https://doi.org/10.1083/jcb.200803042.
7. Giunta, S., Herve, S., White, R.R., Wilhelm, T., Dumont, M., Scelfo, A., Gamba, R., Wong, C.K., Rancati, G., Smogorzewska, A., et al. (2021). CENP-A chromatin prevents replication stress at centromeres to avoid structural aneuploidy. Proc. Natl. Acad. Sci. USA *118*, 2015634118. https://doi.org/10.1073/pnas.2015634118.
8. Barra, V., and Fachinetti, D. (2018). The dark side of centromeres: types, causes and consequences of structural abnormalities implicating centromeric DNA. Nat. Commun. *9*, 4340. https://doi.org/10.1038/s41467-018-06545-y.
9. Mavaddat, N., Peock, S., Frost, D., Ellis, S., Platte, R., Fineberg, E., Evans, D.G., Izatt, L., Eeles, R.A., Adlard, J., et al. (2013). Cancer risks for BRCA1 and BRCA2 mutation carriers: results from prospective analysis of EMBRACE. J. Natl. Cancer Inst. *105*, 812–822. https://doi.org/10.1093/jnci/djt095.
10. Liede, A., Karlan, B.Y., and Narod, S.A. (2004). Cancer risks for male carriers of germline mutations in BRCA1 or BRCA2: a review of the literature. J. Clin. Oncol. *22*, 735–742. https://doi.org/10.1200/JCO.2004.05.055.
11. Kuchenbaecker, K.B., Hopper, J.L., Barnes, D.R., Phillips, K.A., Mooij, T.M., Roos-Blom, M.J., Jervis, S., van Leeuwen, F.E., Milne, R.L., Andrieu, N., et al. (2017). Risks of Breast, Ovarian, and Contralateral Breast Cancer for BRCA1 and BRCA2 Mutation Carriers. JAMA *317*, 2402–2416. https://doi.org/10.1001/jama.2017.7112.
12. Cavanagh, H., and Rogers, K.M.A. (2015). The role of BRCA1 and BRCA2 mutations in prostate, pancreatic and stomach cancers. Hered. Cancer Clin. Pract. *13*, 16. https://doi.org/10.1186/s13053-015-0038-x.
13. Antoniou, A., Pharoah, P.D.P., Narod, S., Risch, H.A., Eyfjord, J.E., Hopper, J.L., Loman, N., Olsson, H., Johannsson, O., Borg, A., et al. (2003). Average risks of breast and ovarian cancer associated with BRCA1 or BRCA2 mutations detected in case Series unselected for family history: a combined analysis of 22 studies. Am. J. Hum. Genet. *72*, 1117–1130. https://doi.org/10.1086/375033.
14. Howlett, N.G., Taniguchi, T., Olson, S., Cox, B., Waisfisz, Q., De Die-Smulders, C., Persky, N., Grompe, M., Joenje, H., Pals, G., et al. (2002). Biallelic inactivation of BRCA2 in Fanconi anemia. Science *297*, 606–609. https://doi.org/10.1126/science.1073834.
15. Grigorova, M., Staines, J.M., Ozdag, H., Caldas, C., and Edwards, P.A.W. (2004). Possible causes of chromosome instability: comparison of chromosomal abnormalities in cancer cell lines with mutations in BRCA1, BRCA2, CHK2 and BUB1. Cytogenet. Genome Res. *104*, 333–340. https://doi.org/10.1159/000077512.
16. Patel, K.J., Yu, V.P., Lee, H., Corcoran, A., Thistlethwaite, F.C., Evans, M.J., Colledge, W.H., Friedman, L.S., Ponder, B.A., and Venkitaraman, A.R. (1998). Involvement of Brca2 in DNA repair. Mol. Cell *1*, 347–357. https://doi.org/10.1016/s1097-2765(00)80035-0.
17. Tutt, A., Gabriel, A., Bertwistle, D., Connor, F., Paterson, H., Peacock, J., Ross, G., and Ashworth, A. (1999). Absence of Brca2 causes genome instability by chromosome breakage and loss associated with centrosome amplification. Curr. Biol. *9*, 1107–1110. https://doi.org/10.1016/s0960-9822(99)80479-5.
18. Xia, B., Sheng, Q., Nakanishi, K., Ohashi, A., Wu, J., Christ, N., Liu, X., Jasin, M., Couch, F.J., and Livingston, D.M. (2006). Control of BRCA2 cellular and clinical functions by a nuclear partner, PALB2. Mol. Cell *22*, 719–729. https://doi.org/10.1016/j.molcel.2006.05.022.
19. Rasheed, S., Nelson-Rees, W.A., Toth, E.M., Arnstein, P., and Gardner, M.B. (1974). Characterization of a newly derived human sarcoma cell line (HT-1080). Cancer *33*, 1027–1033. https://doi.org/10.1002/1097-0142(197404)33:4<1027::aid-cncr2820330419>3.0.co;2-z.
20. Liskovykh, M., Goncharov, N.V., Petrov, N., Aksenova, V., Pegoraro, G., Ozbun, L.L., Reinhold, W.C., Varma, S., Dasso, M., Kumeiko, V., et al. (2019). A novel assay to screen siRNA libraries identifies protein kinases required for chromosome transmission. Genome Res. *29*, 1719–1732. https://doi.org/10.1101/gr.254276.119.
21. Masumoto, H., Masukata, H., Muro, Y., Nozaki, N., and Okazaki, T. (1989). A human centromere antigen (CENP-B) interacts with a short specific sequence in alphoid DNA, a human centromeric satellite. J. Cell Biol. *109*, 1963–1973. https://doi.org/10.1083/jcb.109.5.1963.
22. Nakano, M., Cardinale, S., Noskov, V.N., Gassmann, R., Vagnarelli, P., Kandels-Lewis, S., Larionov, V., Earnshaw, W.C., and Masumoto, H. (2008). Inactivation of a human kinetochore by specific targeting of chromatin modifiers. Dev. Cell *14*, 507–522. https://doi.org/10.1016/j.devcel.2008.02.001.
23. Kouprina, N., Petrov, N., Molina, O., Liskovykh, M., Pesenti, E., Ohzeki, J.I., Masumoto, H., Earnshaw, W.C., and Larionov, V. (2018). Human Artificial Chromosome with Regulated Centromere: A Tool for Genome and Cancer Studies. ACS Synth. Biol. *7*, 1974–1989. https://doi.org/10.1021/acssynbio.8b00230.
24. Molina, O., Kouprina, N., Masumoto, H., Larionov, V., and Earnshaw, W.C. (2017). Using human artificial chromosomes to study centromere assembly and function. Chromosoma *126*, 559–575. https://doi.org/10.1007/s00412-017-0633-x.
25. Lee, H.S., Lee, N.C.O., Grimes, B.R., Samoshkin, A., Kononenko, A.V., Bansal, R., Masumoto, H., Earnshaw, W.C., Kouprina, N., and Larionov, V. (2013). A new assay for measuring chromosome instability (CIN) and identification of drugs that elevate CIN in cancer cells. BMC Cancer *13*, 252. https://doi.org/10.1186/1471-2407-13-252.
26. Natsume, T., Kiyomitsu, T., Saga, Y., and Kanemaki, M.T. (2016). Rapid Protein Depletion in Human Cells by Auxin-Inducible Degron Tagging with Short Homology Donors. Cell Rep. *15*, 210–218. https://doi.org/10.1016/j.celrep.2016.03.001.
27. Oliver, A.W., Swift, S., Lord, C.J., Ashworth, A., and Pearl, L.H. (2009). Structural basis for recruitment of BRCA2 by PALB2. EMBO Rep. *10*, 990–996. https://doi.org/10.1038/embor.2009.126.
28. Grimm, J.B., English, B.P., Chen, J., Slaughter, J.P., Zhang, Z., Revyakin, A., Patel, R., Macklin, J.J., Normanno, D., Singer, R.H., et al. (2015). A general method to improve fluorophores for live-cell and single-molecule microscopy. Nat. Methods *12*, 244–250, 243 p following 250. https://doi.org/10.1038/nmeth.3256.
29. Los, G.V., Encell, L.P., McDougall, M.G., Hartzell, D.D., Karassina, N., Zimprich, C., Wood, M.G., Learish, R., Ohana, R.F., Urh, M., et al. (2008). HaloTag: a novel protein labeling technology for cell imaging and protein analysis. ACS Chem. Biol. *3*, 373–382. https://doi.org/10.1021/cb800025k.

CellPress
OPEN ACCESS

30. Encell, L.P., Friedman Ohana, R., Zimmerman, K., Otto, P., Vidugiris, G., Wood, M.G., Los, G.V., McDougall, M.G., Zimprich, C., Karassina, N., et al. (2012). Development of a dehalogenase-based protein fusion tag capable of rapid, selective and covalent attachment to customizable ligands. Curr. Chem. Genomics *6*, 55–71. https://doi.org/10.2174/1875397301206010055.

31. Waszak, S.M., Northcott, P.A., Buchhalter, I., Robinson, G.W., Sutter, C., Groebner, S., Grund, K.B., Brugières, L., Jones, D.T.W., Pajtler, K.W., et al. (2018). Spectrum and prevalence of genetic predisposition in medulloblastoma: a retrospective genetic study and prospective validation in a clinical trial cohort. Lancet Oncol. *19*, 785–798. https://doi.org/10.1016/S1470-2045(18)30242-0.

32. Sawai, T., Sasano, O., Tsuji, T., Nanashima, A., Yasutake, T., Kusano, H., Tagawa, Y., Nakagoe, T., and Ayabe, H. (1997). [Numerical aberration of chromosome 17 is correlated with multiple primary cancer in colorectal carcinoma]. Nihon Shokakibyo Gakkai Zasshi *94*, 464–468.

33. Birkness, J.E., Spada, N.G., Miller, C., Luketich, J.D., Nason, K.S., Sun, W., and Davison, J.M. (2018). Extreme chromosome 17 copy number instability is a prognostic factor in patients with gastroesophageal adenocarcinoma: A retrospective cohort study. Genes Chromosomes Cancer *57*, 28–34. https://doi.org/10.1002/gcc.22504.

34. Knutsen, T., Padilla-Nash, H.M., Wangsa, D., Barenboim-Stapleton, L., Camps, J., McNeil, N., Difilippantonio, M.J., and Ried, T. (2010). Definitive molecular cytogenetic characterization of 15 colorectal cancer cell lines. Genes Chromosomes Cancer *49*, 204–223. https://doi.org/10.1002/gcc.20730.

35. Zeitlin, S.G., Baker, N.M., Chapados, B.R., Soutoglou, E., Wang, J.Y.J., Berns, M.W., and Cleveland, D.W. (2009). Double-strand DNA breaks recruit the centromeric histone CENP-A. Proc. Natl. Acad. Sci. USA *106*, 15762–15767. https://doi.org/10.1073/pnas.0908233106.

36. Yilmaz, D., Furst, A., Meaburn, K., Lezaja, A., Wen, Y., Altmeyer, M., Reina-San-Martin, B., and Soutoglou, E. (2021). Activation of homologous recombination in G1 preserves centromeric integrity. Nature *600*, 748–753. https://doi.org/10.1038/s41586-021-04200-z.

37. Saayman, X., Graham, E., Nathan, W.J., Nussenzweig, A., and Esashi, F. (2023). Centromeres as universal hotspots of DNA breakage, driving RAD51-mediated recombination during quiescence. Mol. Cell *83*, 523–538.e7. https://doi.org/10.1016/j.molcel.2023.01.004.

38. Yesbolatova, A., Saito, Y., Kitamoto, N., Makino-Itou, H., Ajima, R., Nakano, R., Nakaoka, H., Fukui, K., Gamo, K., Tominari, Y., et al. (2020). The auxin-inducible degron 2 technology provides sharp degradation control in yeast, mammalian cells, and mice. Nat. Commun. *11*, 5701. https://doi.org/10.1038/s41467-020-19532-z.

39. Papadopoulos, N., Nicolaides, N.C., Wei, Y.F., Ruben, S.M., Carter, K.C., Rosen, C.A., Haseltine, W.A., Fleischmann, R.D., Fraser, C.M., Adams, M.D., et al. (1994). Mutation of a mutL homolog in hereditary colon cancer. Science *263*, 1625–1629. https://doi.org/10.1126/science.8128251.

40. Mitra, S., Bodor, D.L., David, A.F., Abdul-Zani, I., Mata, J.F., Neumann, B., Reither, S., Tischer, C., and Jansen, L.E.T. (2020). Genetic screening identifies a SUMO protease dynamically maintaining centromeric chromatin. Nat. Commun. *11*, 501. https://doi.org/10.1038/s41467-019-14276-x.

41. Sonoda, E., Sasaki, M.S., Buerstedde, J.M., Bezzubova, O., Shinohara, A., Ogawa, H., Takata, M., Yamaguchi-Iwai, Y., and Takeda, S. (1998). Rad51-deficient vertebrate cells accumulate chromosomal breaks prior to cell death. EMBO J. *17*, 598–608. https://doi.org/10.1093/emboj/17.2.598.

42. Saayman, X., Graham, E., Leung, C.W.B., and Esashi, F. (2023). exo-FISH: Protocol for detecting DNA breaks in repetitive regions of mammalian genomes. STAR Protoc. *4*, 102487. https://doi.org/10.1016/j.xpro.2023.102487.

43. Bhattacharyya, N.P., Skandalis, A., Ganesh, A., Groden, J., and Meuth, M. (1994). Mutator phenotypes in human colorectal carcinoma cell lines. Proc. Natl. Acad. Sci. USA *91*, 6319–6323. https://doi.org/10.1073/pnas.91.14.6319.

44. Nicolaides, N.C., Papadopoulos, N., Liu, B., Wei, Y.F., Carter, K.C., Ruben, S.M., Rosen, C.A., Haseltine, W.A., Fleischmann, R.D., Fraser, C.M., et al. (1994). Mutations of two PMS homologues in hereditary nonpolyposis colon cancer. Nature *371*, 75–80. https://doi.org/10.1038/371075a0.

45. Bronner, C.E., Baker, S.M., Morrison, P.T., Warren, G., Smith, L.G., Lescoe, M.K., Kane, M., Earabino, C., Lipford, J., Lindblom, A., et al. (1994). Mutation in the DNA mismatch repair gene homologue hMLH1 is associated with hereditary non-polyposis colon cancer. Nature *368*, 258–261. https://doi.org/10.1038/368258a0.

46. Henikoff, S., Ahmad, K., and Malik, H.S. (2001). The centromere paradox: stable inheritance with rapidly evolving DNA. Science *293*, 1098–1102. https://doi.org/10.1126/science.1062939.

47. Logsdon, G.A., Rozanski, A.N., Ryabov, F., Potapova, T., Shepelev, V.A., Catacchio, C.R., Porubsky, D., Mao, Y., Yoo, D., Rautiainen, M., et al. (2024). The variation and evolution of complete human centromeres. Nature *629*, 136–145. https://doi.org/10.1038/s41586-024-07278-3.

48. Spies, M., and Fishel, R. (2015). Mismatch repair during homologous and homeologous recombination. Cold Spring Harb. Perspect. Biol. *7*, a022657. https://doi.org/10.1101/cshperspect.a022657.

49. Sengodan, S.K., Hu, X., Peddibhotla, V., Balamurugan, K., Mitrophanov, A.Y., McKennett, L., Kharat, S.S., Sanawar, R., Singh, V.K., Albaugh, M.E., et al. (2024). Mismatch repair protein MLH1 suppresses replicative stress in BRCA2-deficient breast tumors. J. Clin. Invest. *134*, e173718. https://doi.org/10.1172/JCI173718.

50. Russo, M., Crisafulli, G., Sogari, A., Reilly, N.M., Arena, S., Lamba, S., Bartolini, A., Amodio, V., Magrì, A., Novara, L., et al. (2019). Adaptive mutability of colorectal cancers in response to targeted therapies. Science *366*, 1473–1480. https://doi.org/10.1126/science.aav4474.

51. Skoufias, D.A., DeBonis, S., Saoudi, Y., Lebeau, L., Crevel, I., Cross, R., Wade, R.H., Hackney, D., and Kozielski, F. (2006). S-trityl-L-cysteine is a reversible, tight binding inhibitor of the human kinesin Eg5 that specifically blocks mitotic progression. J. Biol. Chem. *281*, 17559–17569. https://doi.org/10.1074/jbc.M511735200.

52. Minocherhomji, S., Ying, S., Bjerregaard, V.A., Bursomanno, S., Aleliunaite, A., Wu, W., Mankouri, H.W., Shen, H., Liu, Y., and Hickson, I.D. (2015). Replication stress activates DNA repair synthesis in mitosis. Nature *528*, 286–290. https://doi.org/10.1038/nature16139.

53. Scelfo, A., Angrisani, A., Grillo, M., Barnes, B.M., Muyas, F., Sauer, C.M., Leung, C.W.B., Dumont, M., Grison, M., Mazaud, D., et al. (2024). Specialized replication mechanisms maintain genome stability at human centromeres. Mol. Cell *84*, 1003–1020.e10. https://doi.org/10.1016/j.molcel.2024.01.018.

54. Groelly, F.J., Dagg, R.A., Petropoulos, M., Rossetti, G.G., Prasad, B., Panagopoulos, A., Paulsen, T., Karamichali, A., Jones, S.E., Ochs, F., et al. (2022). Mitotic DNA synthesis is caused by transcription-replication conflicts in BRCA2-deficient cells. Mol. Cell *82*, 3382–3397.e7. https://doi.org/10.1016/j.molcel.2022.07.011.

55. McNulty, S.M., Sullivan, L.L., and Sullivan, B.A. (2017). Human Centromeres Produce Chromosome-Specific and Array-Specific Alpha Satellite Transcripts that Are Complexed with CENP-A and CENP-C. Dev. Cell *42*, 226–240.e6. https://doi.org/10.1016/j.devcel.2017.07.001.

56. Chan, F.L., Marshall, O.J., Saffery, R., Kim, B.W., Earle, E., Choo, K.H.A., and Wong, L.H. (2012). Active transcription and essential role of RNA polymerase II at the centromere during mitosis. Proc. Natl. Acad. Sci. USA *109*, 1979–1984. https://doi.org/10.1073/pnas.1108705109.

57. Quenet, D., and Dalal, Y. (2014). A long non-coding RNA is required for targeting centromeric protein A to the human centromere. Elife *3*, e03254. https://doi.org/10.7554/eLife.03254.

58. Bleuyard, J.Y., Fournier, M., Nakato, R., Couturier, A.M., Katou, Y., Ralf, C., Hester, S.S., Dominguez, D., Rhodes, D., Humphrey, T.C., et al. (2017). MRG15-mediated tethering of PALB2 to unperturbed chromatin protects active genes from genotoxic stress. Proc. Natl. Acad. Sci. USA *114*, 7671–7676. https://doi.org/10.1073/pnas.1620208114.

59. Choi, E., Park, P.G., Lee, H.O., Lee, Y.K., Kang, G.H., Lee, J.W., Han, W., Lee, H.C., Noh, D.Y., Lekomtsev, S., and Lee, H. (2012). BRCA2 fine-tunes the spindle assembly checkpoint through reinforcement of BubR1 acetylation. Dev. Cell *22*, 295–308. https://doi.org/10.1016/j.devcel.2012.01.009.

60. Ehlen, A., Martin, C., Miron, S., Julien, M., Theillet, F.X., Ropars, V., Sessa, G., Beaurepere, R., Boucherit, V., Duchambon, P., et al. (2020). Proper chromosome alignment depends on BRCA2 phosphorylation by PLK1. Nat. Commun. *11*, 1819. https://doi.org/10.1038/s41467-020-15689-9.

61. Daniels, M.J., Wang, Y., Lee, M., and Venkitaraman, A.R. (2004). Abnormal cytokinesis in cells deficient in the breast cancer susceptibility protein BRCA2. Science *306*, 876–879. https://doi.org/10.1126/science.1102574.

62. Mondal, G., Rowley, M., Guidugli, L., Wu, J., Pankratz, V.S., and Couch, F.J. (2012). BRCA2 localization to the midbody by filamin A regulates cep55 signaling and completion of cytokinesis. Dev. Cell *23*, 137–152. https://doi.org/10.1016/j.devcel.2012.05.008.

63. Lekomtsev, S., Guizetti, J., Pozniakovsky, A., Gerlich, D.W., and Petronczki, M. (2010). Evidence that the tumor-suppressor protein BRCA2 does not regulate cytokinesis in human cells. J. Cell Sci. *123*, 1395–1400. https://doi.org/10.1242/jcs.068015.

64. Mitelman, F., Johansson, B., and Mertens, F. (2008). Mitelman Database of Chromosome Aberrations in Cancer.

65. Canela, A., Sridharan, S., Sciascia, N., Tubbs, A., Meltzer, P., Sleckman, B.P., and Nussenzweig, A. (2016). DNA Breaks and End Resection Measured Genome-wide by End Sequencing. Mol. Cell *63*, 898–911. https://doi.org/10.1016/j.molcel.2016.06.034.

66. Logsdon, G.A., Vollger, M.R., Hsieh, P., Mao, Y., Liskovykh, M.A., Koren, S., Nurk, S., Mercuri, L., Dishuck, P.C., Rhie, A., et al. (2021). The structure, function and evolution of a complete human chromosome 8. Nature *593*, 101–107. https://doi.org/10.1038/s41586-021-03420-7.

67. Sriramachandran, A.M., Petrosino, G., Mendez-Lago, M., Schafer, A.J., Batista-Nascimento, L.S., Zilio, N., and Ulrich, H.D. (2020). Genome-wide Nucleotide-Resolution Mapping of DNA Replication Patterns, Single-Strand Breaks, and Lesions by GLOE-Seq. Mol. Cell *78*, 975–985.e977. https://doi.org/10.1016/j.molcel.2020.03.027.

68. Owen, B.A.L., Yang, Z., Lai, M., Gajec, M., Badger, J.D., 2nd, Hayes, J.J., Edelmann, W., Kucherlapati, R., Wilson, T.M., and McMurray, C.T. (2005). (CAG)(n)-hairpin DNA binds to Msh2-Msh3 and changes properties of mismatch recognition. Nat. Struct. Mol. Biol. *12*, 663–670. https://doi.org/10.1038/nsmb965.

69. Chan, F.L., and Wong, L.H. (2012). Transcription in the maintenance of centromere chromatin identity. Nucleic Acids Res. *40*, 11178–11188. https://doi.org/10.1093/nar/gks921.

70. Huang, Y., Gu, L., and Li, G.M. (2018). H3K36me3-mediated mismatch repair preferentially protects actively transcribed genes from mutation. J. Biol. Chem. *293*, 7811–7823. https://doi.org/10.1074/jbc.RA118.002839.

71. Li, F., Mao, G., Tong, D., Huang, J., Gu, L., Yang, W., and Li, G.M. (2013). The histone mark H3K36me3 regulates human DNA mismatch repair through its interaction with MutSalpha. Cell *153*, 590–600. https://doi.org/10.1016/j.cell.2013.03.025.

72. van Nuland, R., van Schaik, F.M., Simonis, M., van Heesch, S., Cuppen, E., Boelens, R., Timmers, H.M., and van Ingen, H. (2013). Nucleosomal DNA binding drives the recognition of H3K36-methylated nucleosomes by the PSIP1-PWWP domain. Epigenet. Chromatin *6*, 12. https://doi.org/10.1186/1756-8935-6-12.

73. Di Paolo, A., Racca, C., Calsou, P., and Larminat, F. (2014). Loss of BRCA1 impairs centromeric cohesion and triggers chromosomal instability. FASEB J. *28*, 5250–5261. https://doi.org/10.1096/fj.14-250266.

74. Kononenko, A.V., Bansal, R., Lee, N.C.O., Grimes, B.R., Masumoto, H., Earnshaw, W.C., Larionov, V., and Kouprina, N. (2014). A portable BRCA1-HAC (human artificial chromosome) module for analysis of BRCA1 tumor suppressor function. Nucleic Acids Res. *42*, e164. https://doi.org/10.1093/nar/gku870.

75. Yata, K., Bleuyard, J.Y., Nakato, R., Ralf, C., Katou, Y., Schwab, R.A., Niedzwiedz, W., Shirahige, K., and Esashi, F. (2014). BRCA2 coordinates the activities of cell-cycle kinases to promote genome stability. Cell Rep. *7*, 1547–1559. https://doi.org/10.1016/j.celrep.2014.04.023.

76. Yata, K., Lloyd, J., Maslen, S., Bleuyard, J.Y., Skehel, M., Smerdon, S.J., and Esashi, F. (2012). Plk1 and CK2 act in concert to regulate Rad51 during DNA double strand break repair. Mol. Cell *45*, 371–383. https://doi.org/10.1016/j.molcel.2011.12.028.

77. Zhang, F., Ma, J., Wu, J., Ye, L., Cai, H., Xia, B., and Yu, X. (2009). PALB2 links BRCA1 and BRCA2 in the DNA-damage response. Curr. Biol. *19*, 524–529. https://doi.org/10.1016/j.cub.2009.02.018.

78. Li, R., Zhang, R., Tan, P., Wang, M., Chen, Y., Zhang, J., Han, D., Han, Y., Li, J., and Zhang, R. (2021). Development of novel quality control material based on CRISPR/Cas9 editing and xenografts for MLH1 protein deficiency testing. J. Clin. Lab. Anal. *35*, e23746. https://doi.org/10.1002/jcla.23746.

79. Schneider, C.A., Rasband, W.S., and Eliceiri, K.W. (2012). NIH Image to ImageJ: 25 years of image analysis. Nat. Methods *9*, 671–675. https://doi.org/10.1038/nmeth.2089.

80. Schindelin, J., Arganda-Carreras, I., Frise, E., Kaynig, V., Longair, M., Pietzsch, T., Preibisch, S., Rueden, C., Saalfeld, S., Schmid, B., et al. (2012). Fiji: an open-source platform for biological-image analysis. Nat. Methods *9*, 676–682. https://doi.org/10.1038/nmeth.2019.

81. Gilles, J.F., Dos Santos, M., Boudier, T., Bolte, S., and Heck, N. (2017). DiAna, an ImageJ tool for object-based 3D co-localization and distance analysis. Methods *115*, 55–64. https://doi.org/10.1016/j.ymeth.2016.11.016.

82. Waskom, M. (2021). seaborn: statistical data visualization.

83. van Rossum, G. (1995). Python reference manual.

84. Hunter, J.D. (2007). Matplotlib: A 2D Graphics Environment. Comput. Sci. Eng. *9*, 90–95.

85. Harris, C.R., Millman, K.J., van der Walt, S.J., Gommers, R., Virtanen, P., Cournapeau, D., Wieser, E., Taylor, J., Berg, S., Smith, N.J., et al. (2020). Array programming with NumPy. Nature *585*, 357–362. https://doi.org/10.1038/s41586-020-2649-2.

86. McKinney, W. (2010). Data Structures for Statistical Computing in Python. In SciPy, pp. 56–61. https://doi.org/10.25080/Majora-92bf1922-00a.

87. Heck, N., Dos Santos, M., Amairi, B., Salery, M., Besnard, A., Herzog, E., Boudier, T., Vanhoutte, P., and Caboche, J. (2015). A new automated 3D detection of synaptic contacts reveals the formation of cortico-striatal synapses upon cocaine treatment in vivo. Brain Struct. Funct. *220*, 2953–2966. https://doi.org/10.1007/s00429-014-0837-2.

CellPress
OPEN ACCESS

STAR★METHODS

KEY RESOURCES TABLE

REAGENT or RESOURCE	SOURCE	IDENTIFIER
**Antibodies**		
BRCA2	Millipore	Cat#OP95; RRID:AB_213443
RAD51	Biogenes Custom (Originally reported at Yata et al., 2014[75]; PMID 24835992, also used in Saayman et al.[37])	Cat#7946
PALB2	Biorbyt	Cat#orb412704
Lamin A	Sigma Aldrich	Cat#L1293; RRID:AB_532254
Histone H3 (phospho S10)	Millipore	Cat#06-570; RRID:AB_310177
RAD51	Abcam	Cat#ab176458; RRID:AB_2665405
CENP-A	GeneTex	Cat#GTX13939; RRID:AB_369391
CENP-C	Millipore	Cat#ABE1957; RRID:AB_3075449
CENP-B	Bethyl Laboratories	Cat#IHC-00064; RRID:AB_669682
CENP-B	Santa Cruz Biotechnology	Cat#sc-376283; RRID:AB_10988421
CENP-T	Abcam	Cat#ab220280; RRID:AB_2938694
ACA (anti-centromere)	Antibodies Incorporated	Cat#15-234-0001; RRID:AB_2687472
γH2A.X	Millipore	Cat#clone JBW301, 05-636; RRID:AB_309864
Cyclin B1	Santa Cruz	Cat#sc-245; RRID:AB_627338
STK38	Abnova	Cat#H00011329-M01; RRID:AB_540741
MLH1	Abcam	Cat#Ab92312; RRID:AB_2049968
Alpha Tubulin	Cell Signaling Technology	Cat#3873S; RRID:AB_1904178
IgG	Invitrogen	Cat#02-6502; RRID:AB_2532951
Goat anti-Rabbit HRP	Dako	Cat#P0448; RRID:AB_2617138
Goat anti-Mouse HRP	Dako	Cat#P0447; RRID:AB_2617137
Goat anti-Mouse Alexa Fluor 647	Invitrogen	Cat#A21235; RRID:AB_2535804
Goat anti-Rabbit Alexa Fluor 647	Invitrogen	Cat#A21244; RRID:AB_2535812
Goat anti-Mouse Alexa Fluor 555	Invitrogen	Cat#A21422; RRID:AB_141822
Goat anti-Rabbit Alexa Fluor 555	Invitrogen	Cat#A21428; RRID:AB_141784
Goat anti-Rabbit Alexa Fluor 488	Invitrogen	Cat#A11070; RRID:AB_142134
Goat anti-Mouse Alexa Fluor 488	Invitrogen	Cat#A11017; RRID:AB_2534084
Anti-human Alexa Fluor 647	Jackson ImmunoResearch	Cat#109-605-088; RRID:AB_2337887
**Bacterial and virus strains**		
TOP10	Invitrogen	Cat#C404010
**Chemicals, peptides, and recombinant proteins**		
Penicillin-streptomycin solution	Life Technologies	Cat#15140122
Sodium bicarbonate solution	Sigma Aldrich	Cat#S8761
Blasticidin	Invivogen	Cat#ant-bl-1
G418	Sigma Aldrich	Cat#G8168
Exonuclease III	Promega	Cat#M1811
Mitomycin C	Abcam	Cat#Ab120797
Lipofectamine LTX	Thermo Fisher Scientific	Cat#A12621
Lipofectamine RNAiMAX	Thermo Fisher Scientific	Cat#13778075
FISH blocking solution	Roche	Cat#11096176001
Indole-3-acetic acid (IAA)	Sigma Aldrich	Cat#I5148

(*Continued on next page*)

Continued		
REAGENT or RESOURCE	SOURCE	IDENTIFIER
5-Ethynyl-2′-deoxyuridine (EdU)	Abcam	Cat#ab146186
$CuSO_4$	Sigma Aldrich	Cat#209198
Sodium L-ascorbate	Sigma Aldrich	Cat#A4034
Alexa Fluor Azide 555	Life Technologies	Cat#A20012
DAPI	BD Biosciences	Cat#564907
Propidium iodide	Sigma Aldrich	Cat#P4864
RNAse A	Sigma Aldrich	Cat#R6513
DRAQ7	Abcam	Cat#ab109202
Benzonase	EMD Millipore	Cat#70746
Protease inhibitor cocktail	Sigma Aldrich	Cat#P2714
Bradford reagent	Bio-Rad	Cat#5000006
ECL Western blotting detection solution	Amersham	Cat#RPN2106
Colcemid	Millipore Sigma	Cat#234109
S-trityl-L-cysteine (STLC)	Tocris	Cat#2191
Formamide	Sigma Aldrich	Cat#47671
Mowiol 4-88	Millipore	Cat#475904
PIPES pH 6.5	Sigma Aldrich	Cat#P1851
Pierce^{TM} 16% Formaldehyde (w/v), Methanol-free	Thermo Fisher Scientific	Cat#28908
ProLong^{TM} Gold Antifade Mountant with DAPI	Invitrogen	Cat#P36935
DMSO	Sigma Aldrich	Cat#D2650-100ML
Hoechst 33342	Molecular probes	Cat#H-3570
WST-1 reagent	Roche	Cat#5015944001
PfuUltra II Fusion HS DNA polymerase	Agilent Technologies	Cat#600670
BbsI	New England Biolabs	Cat#R3539
Quick CIP	New England Biolabs	Cat#M0525
TRIzol reagent	Invitrogen	Cat#15596026
1-Bromo-3-chloropropane	Sigma Aldrich	Cat#B9673
Ammonium acetate solution	Sigma Aldrich	Cat#A2706
GlycoBlue Coprecipitant	Invitrogen	Cat#AM9515
Janelia Fluor 549 Halo-ligand	Gift from Lavis lab; Promega	Cat#GA1110
Triton^{TM} X-100	Sigma-Aldrich	Cat#93443-100ML
Bovine Serum Albumin	Sigma-Aldrich	Cat#A7906-100G
Critical commercial assays		
cDNA reverse transcription kit	Applied Biosystems	Cat#4368814
Cell-Trace^{TM} Far Red Cell Proliferation Kit	Invitrogen	Cat#C34564
Takara DNA ligation kit, Mighty Mix	Takara Bio Europe	Cat#6023
TURBO DNA-free kit	Invitrogen	Cat#AM1907
SensiFAST SYBR No-ROX kit	Meridian Bioscience	Cat#BIO-98005
Deposited data		
Raw and analyzed data	This paper; Mendeley Data	https://doi.org/10.17632/v7ym4ff28n.1
Experimental models: Cell lines		
Human: hTERT-RPE1	ATCC	RRID:CVCL 4388
Human: hTERT-RPE1 MLH1 KO	This study	N/A
Human: HT1080 alphoid^{tetO} dGFP-HAC	Liskovykh et al.[20]	https://doi.org/10.1101/gr.254276.119
Human: HCT116	ATCC	RRID:CVCL0291
Human: HCT116 OsTIR1	Natsume et al.[26]	https://doi.org/10.1016/j.celrep.2016.03.001
Human: HCT116 OsTIR1 mAID-Halo-BRCA2	This study	N/A

(Continued on next page)

CellPress
OPEN ACCESS

Continued

REAGENT or RESOURCE	SOURCE	IDENTIFIER
Human: HCT116 OsTIR1 PALB2-mAID-Halo	This study	N/A
**Oligonucleotides**		
Mission Negative siRNA	Sigma Aldrich	Cat#SIC001
siRNA targeting BRCA2 (1): CAACAAUUACGAACCAAACdTdT	Yata et al.[76]	N/A
siRNA targeting BRCA2 (2): CUGAGCAAGCCUCAGUCAAdTdT	Yata et al.[76]	N/A
siRNA targeting RAD51 (1): GACUGCCAGGAUAAAGCUUdTdT	Yata et al.[76]	N/A
siRNA targeting RAD51 (2): GUGCUGCAGCCUAAUGAGAdTdT	Yata et al.[76]	N/A
siRNA targeting PALB2 (1): GGAAAGAGCCGGUUGUAAAdTdT	Zhang et al.[77]	N/A
siRNA targeting PALB2 (2): GGAGAAAUUAGCAUUCUUGdTdT	Zhang et al.[77]	N/A
siRNA targeting STK38: CCUUAUCGCUCAACAUGAAdTdT	Liskovykh et al.[20]	N/A
siRNA targeting CENP-A: GGACUCUCCAGAG CCAUGAUUdTdT	Giunta et al.[5]	N/A
PALB2_CHK_F primer: GAAGCTTGCACTGTTTGAGAG C	This study	N/A
PALB2_CHK_R primer: GTACATCCAAGATCAGTGGTG C	This study	N/A
BRCA2_CHK_F primer: TCCCTGGGTCTCCATTTCCC	This study	N/A
BRCA2_CHK_R primer: GCTAGTCAAGGGGCCAGTTT	This study	N/A
Alpha_fw primer: ATGTTTGCATTCAACTCACAGAG	Yilmaz et al.[36]	N/A
Alpha_rev primer: CAACACAGTCCAAATATCCAGTTG	Yilmaz et al.[36]	N/A
GAPDH_fw primer: ACTGCCACCCAGAAGACTGT	This study	N/A
GAPDH_rev primer: CAGGTCAGGTCCACCACTGA	This study	N/A
sgMLH1_guide 1: CACCGCGAATATTGTCCACGGTTG	Li et al.[78]	N/A
sgMLH1_guide 1: AAACCAACCGTGGACAATATTCGC	Li et al.[78]	N/A
FISH probe CENPB-RC-Cy3: TCCCGTTTCCAACGAAT	Panagene	Cat#F3009
FISH probe TelC-Cy5: CCCTAACCCTAACCCTAA	PNABio	Cat#F1003
PNA-FITC-tetO: [FITC-OO]-ACC ACT CCC TAT CAG	Lee et al.[25]	N/A
PNA-CENPB-Cy5: ATTCGTTGGAAACGGGA	PNABio	Cat#F3005
XL Iso(17q)	MetaSystems	Cat#D-5048-100-OG
PNA bio S.2-A488: CGAGTCCATTCGATGAT	Saayman et al.[37]	N/A
PNA bio S.3-A488: CGAGTCCATTCGATGAT	Saayman et al.[37]	N/A
**Recombinant DNA**		
pSpCas9(BB)-2A-GFP (PX458)	Addgene	Cat#48138
pSpCas9(BB)-2A-Puro (PX459)	Addgene	Cat#62988
pX458_pSpCas9(BB)-2A-GFP-BRCA2-gRNA	This study	N/A
pX459_pSpCas9(BB)-2A-MinusPuro-GFP-PALB2-gRNA	This study	N/A
pMK341_NeoR_mAID_Halo_BRCA2	This study	N/A
pBSKS_PALB2_mAID_Halo_NeoR	This study	N/A
**Software and algorithms**		
FlowJo v10.5.3 and 10.7.2	Becton, Dickinson and Company	https://www.flowjo.com; RRID:SCR_008520
ImageJ v2.0.0-rc-69/1.52p	Schneider et al.[79]	https://imagej.net/software/imagej/
Olympus FV1000 software FV10-ASW v4.2	EVIDENT	RRID:SCR_014215
CytExpert v2.3.0.84	Beckman Coulter	N/A
OLYMPUS cellSens Dimension 3.2	Olympus	www.olympus-sis.com
Fiji v2.0.0-rc-69/1.52p	Schindelin et al.[80]	https://imagej.net/software/fiji/; RRID:SCR_002285
Prism v7, v8 and v9	GraphPad	https://www.graphpad.com; RRID:SCR_002798
DiAna v1.52	Gilles et al.[81]	N/A
Seaborn v0.11.1	Waskom et al.[82]	N/A
Rotor-Gene Q Series Software v.2.3.1	Qiagen	RRID:SCR_015740

(*Continued on next page*)

Continued		
REAGENT or RESOURCE	SOURCE	IDENTIFIER
Python v3.8.8	van Rossum[83]	RRID:SCR_008394
Matplotlib v3.4.2	Hunter[84]	RRID:SCR_008624
NumPy v1.19.1	Harris et al.[85]	RRID:SCR_008633
Pandas v1.2.3	McKinney[86]	RRID:SCR_018214

EXPERIMENTAL MODEL AND STUDY PARTICIPANT DETAILS

Cell lines

All cell lines were cultured in a humidified environment at 37°C supplemented with 5% CO_2. hTERT-immortalised retinal pigmented epithelial cells (RPE1, RRID:CVCL 4388) were maintained in 1:1 Dulbecco Modified Eagle Medium (DMEM)/Nutrient Mixture F12 supplemented with 10% fetal bovine serum (FBS) (F9665, Merck), 1% penicillin-streptomycin (15140122, Life Technologies) and 0.123% sodium bicarbonate (S8761, Sigma-Aldrich). HT1080 alphoid^{tetO} HAC/dGFP cells with maintained in DMEM with 10% FBS, 1% penicillin-streptomycin, and 15 μg/mL blasticidin. HCT116 cells were maintained in McCoy's 5A (Modified) medium, 10% FBS, and 1% penicillin-streptomycin. 5 or 500 μM Indole-3-acetic acid (IAA) (I5148, Sigma-Aldrich) was added for the indicated time periods.

METHOD DETAILS

Materials and methods

RNAi depletion

For DNA transfection, cells were transfected using Lipofectamine LTX (Thermo Fisher Scientific) according to the manufacturer's instructions. 24 h post transfection, the transfection media was replaced with fresh complete media. For siRNA transfection in HT1080 alphoid^{tetO} dGFP-HAC and hTERT RPE1 cells, Lipofectamine RNAiMAX (13778150, Thermo Scientific) was used to transfect the cells according to manufacturer's instructions. A final concentration of 17 nM siRNA was used for siMisNeg, siBRCA2, siRAD51, siGFP, and siSTK38 and 42nM siRNA for siPALB2 in HT1080 alphoid^{tetO} dGFP-HAC cells, and a final concentration of 20 nM siRNA was used for all RNA depletion experiments including siMisNeg negative control (SIC001, Sigma-Aldrich) in RPE1 cells.

Flow cytometry

Cells were harvested by trypsinisation, washed with PBS, and fixed in 70% ice-cold ethanol −20°C for at least 1 h or overnight, 4°C. All subsequent steps were performed on ice and centrifugation steps performed at 1000 x *g* for 5 min at 4°C. Fixed cells were permeabilised in PBS-TriBSA (0.1% v/v Triton X-100, 1% w/v BSA, PBS) for 15 min and washed twice in PBST-BSA (0.1% v/v Tween 20, 1% w/v BSA, PBS). For EdU labeling to mark cell proliferation, cells were incubated 10 μM EdU for 30 min before harvest. After fixation and permeabilisation, cells were incubated in Click-IT reaction buffer (PBS pH 7.2, 2 mM $CuSO_4$ (209198, Sigma-Aldrich), 10 mM sodium L-ascorbate (A4034, Sigma-Aldrich), Alexa Fluor Azide 555 (A20012, Life Technologies)) for 1 h, room temperature. Coverslips were next washed thrice with PBS before proceeding with antibody staining, as required. Primary and secondary antibodies (key resources table), both diluted in PBST-BSA, were added for 1 h and 30 min, respectively. Finally, cells were counterstained with DAPI staining solution (0.1% BSA, 0.1 mg/mL RNase A, 1 μg/mL DAPI, PBS) or propidium iodide (PI) staining solution (PBS, 0.1% BSA, 0.1 mg/mL RNAse A, 2 μg/mL propidium iodide) for at least 30 min before analysing.

Cells were analyzed using a Cytek DxP8 flow cytometer and the CytoFLEX LX (Beckman Coulter) equipped with the CytExpert program (version 2.3.0.84). Obtained data was analyzed using FlowJo analysis software (versions 10.5.3 and 10.7.2). Cell debris was excluded through FSC vs. SSC gating and single cells identified through FSC-H vs. FSC-W gating. Percentage cells in G1, S, and G2/M were obtained through gating distinct cell populations.

HAC stability assay

HAC stability assay in HT1080 alphoid^{tetO} dGFP-HAC cells was carried out as previously described.[20] Then, cells were grown without blasticidin for an additional 24 h. At 72 h after transfection, cells were collected by trypsinisation, washed once in PBS and resuspended in PBS with 0.3 μM DRAQ7 to distinguish live and dead cells. Cells were analyzed using a Cytek DxP8 Flow cytometer and data were analyzed using FlowJo analysis software (version 10.5.3). For the analysis, single cells were selected FSC vs. FSCW, and the proportion of GFP+ live cells/live cells was used to calculate the rate of HAC loss compared to GFP negative parental HT1080 cells. The rate of HAC loss after siRNA treatment was calculated using the formula: $R_{si} = 2 - 2 \cdot \left(\frac{P_{si}}{P_0}\right)^{\frac{1}{n}}$ where R_{si} is the rate of HAC loss after siRNA treatment; P_{si} is the percentage of GFP+ cells after drug treatment; P_0 is the percentage of GFP+ cells at the beginning of the experiment in cells cultured with blasticidin; *n* is the number of cell divisions at the time of the analysis.[35] In this study, n was additionally determined using the dye dilution assay for each siRNA treatment and the values of DI were incorporated in the calculation of the rate of HAC loss. The ratio between the percentage of GFP+ cells after treatment and the blasticidin-positive growing cells is used to calculate the rate of HAC loss. Cells that stably maintain the HAC will display high GFP signal and a low rate of HAC loss, while cells that lose the HAC have a low GFP signal and high rate of HAC loss.

Dye dilution assay

To determine *n* number of cell divisions (division index, DI) accurately for rate of HAC loss calculations we performed dye dilution assays. HT1080 alphoid^{tetO} dGFP-HAC cells were stained following the manufacturer's protocol (Cell-Trace Far Red Cell Proliferation Kit, C34564, Invitrogen). Briefly, 1x10^6 cells were incubated with Cell Trace Far Red staining solution (PBS, 0.5 mM Cell Trace Far Red solution) for 20 min at 37°C. Pelleted cells were resuspended in 4 volumes of complete medium and incubated for 5 min at 37°C to absorb unbound dye. 3–5x10^5 cells were seeded into fresh complete medium. 24 h post seeding, cells were harvested to determine the level of Cell-Trace Far Red fluorescent signal of the undivided population. After siRNA treatment, cells were harvested by trypsinisation, washed once in PBS and resuspended to a final concentration of 1 x 10^6 cells/mL in PBS containing 50 ng/mL DAPI to distinguish live and dead cells. Cells were analyzed using a Cytek DxP8 Flow cytometer using RedFL1 and VioFL1 channels. The obtained proliferation curve was fitted using FlowJo analysis software (version 10.5.3). Parameters were fixed at Peak Ratio: 0.5; Number of peaks: 6–8; Peak CV was based on the undivided population analyzed at 24 h. At 72 h, parental cells showed a DI = 3.62, which is close to the estimated number of cell divisions based on the doubling time of HT1080 cells (i.e., DI = 4 at 72 h).

Western Blotting

Whole cell extracted were prepared using NETN150 lysis buffer (50 mM Tris-HCl pH 8.0, 150 mM NaCl, 2 mM EDTA, 0.5% NP-40, 10 mM Benzamidine HCl, 10 mM NaF, 1 mM sodium glycerophosphate, 1 mM dithiothreitol, with 125 U/mL Benzonase and protease inhibitor cocktail (P2714, Sigma-Aldrich), incubating for at least 30 min on ice. Protein concentration was measured by the Bradford protein assay (Bio-Rad) according to the manufacturer's instructions. Samples were prepared for SDS-PAGE with 1X NuPAGE LDS sample buffer and 25 mM DTT and heated at 80°C–85°C for 5 min. SDS-PAGE was performed in 1X MOPS-SDS running buffer (0.2 mM MOPS, 0.2 mM Tris, 0.1% SDS, 4 mM EDTA) at 100-150V using NuPAGE 4–12% Bis-Tris gels. Protein transfer onto nitrocellulose membranes was performed using a Mini Trans-Blot system at 10V overnight or 100V for 1 h in Transfer Buffer (100 mM Tris, 767 mM Glycine, 15% v/v methanol). Protein transfer was confirmed with Ponceau staining solution (0.1% Ponceau in 5% acetic acid). The membrane was blocked for at least 30 min in Blocking buffer (PBS with 5% milk and 0.05% Tween 20). Primary and secondary antibodies (key resources table) were diluted in Blocking buffer and applied for 1 h at room temperature or overnight at 4°C. Membranes were washed in PBS with 0.05% Tween 20 buffer three times for 5 min. Proteins were detected using 1:1 ECL Western Blotting detection solution (RPN2106, Amersham) for 30 s and developed using chemiluminescence film (28906837, Cytiva) in a Xograph developer, as required.

Immunoprecipitation (IP)

IgG (02–6502, Invitrogen) or BRCA2 antibodies (OP95, Calbiochem) were conjugated to Affi-Prep Protein A agarose beads (1560006, Bio-Rad) using the following protocol. The beads were washed twice with PBS before incubating with the relevant antibody (2 μL antibody per 5 μL beads) on a rotor wheel at room temperature for 3 h. Antibody-crosslinked beads were pelleted (800 x g, 4 min) and washed twice in coupling buffer (27 mM sodium tetraborate (221732, Sigma-Aldrich), 73 mM boric acid (B7660, Sigma-Aldrich)), once in DIM-PIM buffer (56 mM dimethyl pimelimidate dihydrochloride (D8388, Sigma-Aldrich), 0.1 M sodium tetraborate), and then incubated in DIM-PIM buffer overnight, 4°C. Beads were subsequently washed thrice in coupling buffer and once in 1 M Tris pH 9. A further 1 M Tris pH 9 was added for 10 min, room temperature. Finally, the beads were washed thrice in storage buffer (6.5 mM sodium tetraborate, 93.5 mM boric acid) and kept at 4°C until required. Before incubation with protein lysate, antibody-crosslinked beads were prewashed three times with wash buffer (50 mM Tris-HCl pH 8, 150 mM NaCl, 2 mM EDTA, 5 mM $MgCl_2$).

For IP, whole cell lysates were prepared as according to the western blotting protocol above. After protein input sample was taken and stored at −20°C, WCEs were diluted to 1 mg/mL and incubated with 10 μL antibody-crosslinked beads on a rotor wheel, overnight, 4C. Protein-bound beads were pelleted and washed 4 times with wash buffer. Beads and input samples were resuspended in 1X NuPAGE LDS sample buffer (NP0007, Invitrogen) with 200 mM DTT (D0632, Sigma-Aldrich). Input samples were denatured at 85°C for 5 min and the beads for 10 min before western blotting as described previously.

Fluorescence In situ *hybridisation (FISH)*

To prepare metaphase spreads, cells were treated with 0.1 μg/mL colcemid for 3-6h. Cells were collected by mitotic shake-off, washed with PBS and incubated with 0.56% KCl solution for 20 min at room temperature. Cells were centrifuged at 300 x g for 3 min and all but 100 μL supernatant was removed. The cell pellet was gently resuspended in the remaining liquid before fixation in 3:1 Methanol:Acetic acid for 15 min at room temperature, adding the first 1 mL dropwise with constant gentle cell agitation to minimise cell aggregation. Cells were centrifuged as before, and all but 60 μL (~10 μL/slide) supernatant was removed. Fixed cells were spread onto glass slides by dropping 10 μL cell suspension onto a 20 μL water droplet.

For FISH staining against HAC in HT1080 alphoid^{tetO} dGFP-HAC cells, dry slides were rehydrated in PBS for 15 min, and fixed in 4% formaldehyde for 2 min, followed by washing thrice with PBS and dehydrated in a series of ethanol series (70%, 90% and 100%). After air drying for 10–15 min, hybridisation buffer (10 mM Tris-HCl, pH 7.2–7.5, 70% Formamide, 5% Dextran sulfate, 0.25 mM PNA-FITC-tetO (Panagene),[25] 0.25 mM PNA-CENPB-Cy5 (PNA bio)) was applied to the slides and covered with a glass coverslip. Samples were denaturation at 80°C for 3 min followed by hybridisation for 2 h at room temperature, protected from light. Slides were washed twice in formamide washing solution (70% formamide, 10 mM Tris pH 7.2, 0.1% BSA) and thrice in TBS-T (PBS with 0.08% Tween 20), for 5 min each. Slides were washed twice with PBS and incubated with 1 mg/mL DAPI in PBS for 30 min. Slides were washed once more in PBS and dehydrated in a series of 70%, 90% and 100% ethanol washes and mounted. HAC containing metaphases

were analyzed using Olympus Microscope FV1000. Approximately 50 metaphases per sample were analyzed per experiment. For the representative picture, a Z-stack of 10 images was acquired and analyzed using ImageJ analysis software (version 2.0.0-rc-69/1.52p).

For FISH staining against Chromosome 17 in HCT116 cells, metaphase spreads were rehydrated in PBS for 5 min before dehydrating again in increasing ethanol concentrations (70%, 95%, 100%) for 5 min each. Once air-dry, Iso(17q) FISH probe (D-5048-100-OG, Metasystems) was applied to the spreads and covered with a glass slide. The slide was then denatured at 75°C for 3 min and incubated in a humidified dark box at 37°C overnight. Samples were washed in preheated 0.4X SSC pH 7 (60 mM NaCl, 6 mM sodium citrate) at 72°C for 3 min before washing in 2X SSC (300 mM NaCl, 30 mM sodium citrate) with 0.05% v/v Tween 20 pH 7 at room temperature for 1 min. The slides were rinsed with dH2O, incubated with 0.2 μg/mL DAPI for 10 min, washed three more times in dH2O, and dehydrated in sequential ethanol concentrations as before. Slides were left to air dry for at least 30 min before mounting with Mowiol mounting medium (10% w/v Mowiol 4–88 (475904, Millipore), 25% w/v glycerol, 0.1 M Tris pH 8.5). Samples were imaged using Olympus FluoView FV1200 using FV10-ASW software (version 4.2). Frequency of aberrations on labeled Chromosome 17 were scored as a percentage of total chromosomes/spread.

Immunofluorescence (IF)

After the indicated siRNA or IAA treatment, cells were seeded onto coverslips pre-treated with poly-L-lysine for 15 min at 4°C, before fixation using PTEMF buffer (20 mM PIPES pH 6.5 (P1851, Sigma-Aldrich), 10 mM EGTA, 0.2% v/v Triton X-100 (93443, Sigma-Aldrich), 1 mM MgCl2, 4% v/v paraformaldehyde (28908, Thermo Scientific)) for 20 min and then washed three times with PBS. Cells were incubated with blocking buffer (3% w/v BSA (A7906, Sigma-Aldrich), 0.5% v/v Triton X-100) for at least 30 min at room temperature before incubation with the relevant primary and secondary antibodies (key resources table), diluted in blocking buffer, at RT, for 1 h each. Coverslips were washed 3 times with PBS-TX (0.5% v/v Triton X-100, PBS) for 5 min. Cells were stained with 0.1 μg/mL DAPI (564907, BD Biosciences) in PBS for 10 min, washed twice with PBS, and once with dH_2O. Coverslips were left to air-dry completely, before mounting using Mowiol mounting media (10% w/v Mowiol 4–88 (475904, Millipore), 25% w/v glycerol, 0.1 M Tris pH 8.5). Samples were imaged using Olympus FluoView FV1200 using FV10-ASW software (version 4.2). Cells to be imaged and analyzed were selected in an unbiased manner based on DAPI staining.

To assess mitotic index, HT1080 alphoid^{tetO} dGFP-HAC cells were grown on coverslips and fixed in 4% v/v paraformaldehyde. Coverslips were mounted with ProLong Gold Antifade Mountant with DAPI on glass slides. Cells were separated into three categories based on the DAPI stain: cells in interphase, early mitosis or late mitosis.

Colocalisation assay of BRCA2 and PALB2 with centromeres and RAD51

HCT116 cells were seeded in 8-well ibidi dishes (80806, Ibidi) 2 days prior drug treatments. Asynchronous cell populations were first treated with 500 μM Indole-3-acetic acid (IAA) (I5148-2G, Sigma Aldrich) or nuclease free water (BP2484-100, Fisher bioreagents) for 2 h, then with Dimethyl sulfoxide (DMSO) (D2650-100ML, Sigma-Aldrich) together with IAA or nuclease free water for another 2 h. BRCA2 and PALB2 were visualised using 125 nM Janelia Fluor 549 Halo-ligand (gift from the Lavis lab) incubated for 25 min at 37°C with 5% CO2 and then washed 3 times with culture media 30 min before fixing. Cells were washed once with PBS and then fixed in 4% v/v formaldehyde prepared in PBS (28908, Thermo Scientific) for 10 min. Cells were permeabilized in 0.1% v/v Triton X-100 (93443-100ML, Sigma-Aldrich) for 10 min, washed once with PBS, and then incubated with the blocking buffer (2% w/v BSA solution (A7906-100G, Sigma-Aldrich)) for at least 1 h at room temperature. Samples were incubated with the relevant primary antibodies and secondary antibodies (key resources table) prepared in blocking buffer for 1 h respectively, with three 5 min PBS washes in between. DNA was stained with 1μg/mL Hoechst 33342 (H-3570, Molecular probes) for 10 min. Samples were washed in PBS twice, and then stored in PBS at 4°C until imaging. Samples were imaged with Olympus SoRa Spinning disc confocal microscope using the CellSense Software. Fields of view were selected in an unbiased manner based on DNA staining. For each sample 5–8 fields of view were collected with 15 Z-stacks spanning 10.65 μm using the UPlanXApo 60x objective.

Volume overlap analysis

Image analysis was done in the software Fiji.[80] Raw files were first converted to TIF format, then nuclei were segmented in 3D using Otsu thresholding, and voxel intensity values outside the nuclei were set to be 0.

Using the maximum Z-projection of the image, a secondary nuclear segmentation was performed, which was then used to individualise and duplicate the Z-stacks of nuclei. These were saved as individual TIF files. Nuclei were selected for analysis based on the following criteria for exclusion: 1) nuclei of mitotic cells, 2) nuclei on the edge of the image, 3) nuclei with an area larger than 300 μm,[2] 4) overlapping nuclei, 5) nuclei out of focus, 6) nuclei with incomplete or wrong segmentation, 6) nuclei with many apoptotic phenotypes.

The Z-stacks of nuclei were randomised from all the images in each condition, and the first 100 nuclei per condition were further analyzed.

Foci segmentation and volume overlap measurements were done using the Fiji plugin DiAna v1.52.[81] For the segmentation of foci, the spot segmentation feature of DiAna was used[87] with parameters defined based on references images of real data. For the colocalisation analysis, foci volume overlap was used, with the minimum threshold of 10% overlap applied to exclude false positive events. Data was plotted and analyzed in GraphPad Prism 8.1.1. For statistical analysis, paired t test was used between the means of replicates with and without treatment with IAA.

exo-FISH

Interphase cells treated with siRNA or 5 μM IAA where indication and collected by trypsinisation at the required time points. Cells were washed with PBS, counted, and 1.2 x 10^5 cells (2 x 10^4/slide) were collected per 15 mL falcon tube. Cell slides were prepared as described previously for metaphase spreads. Cell density can influence FISH signal intensity so cell number per slide was kept consistent across samples and experiments. Cell spreads were allowed to dry overnight at room temperature in the dark. Homogeneous cell spreading was confirmed via light microscopy.

The next day, slides were rehydrated in PBS for 5 min at room temperature in Coplin jars and treated with 0.5 mg/mL RNase A (R6513, Sigma-Aldrich) for 10 min at 37°C in a humidified chamber, before rinsing briefly with PBS. Next, slides were incubated with 0–200 mU/μL exonuclease III (M1811, Promega) in 1X Exonuclease III buffer (50 mM Tris-Cl, 5 mM $MgCl_2$, 5 mM DTT pH 8) for 30 min at 37°C in a humidified chamber. Slides were washed with PBS and serially dehydrated in 70%, 95%, and 100% ethanol, for 5 min each before air-drying. Slides were hybridised with 200 nM CENPB-RC-Cy3 (F3009, PNABio) and 200 nM TelC-Cy5 (F1003, PNABio) for 1.5 h at RT, in a dark, humidified chamber. Before treatment, the FISH probes were diluted in hybridisation buffer (10 mM Tris-Cl pH 7.5, 70% formamide (47671, Sigma-Aldrich), 0.5% blocking solution (10% w/v blocking reagent (11096176001, Roche) dissolved in maleic acid buffer (100 mM maleic acid, 150 mM NaCl, pH 7.5))) and heated at 60°C for 5 min before use. FISH probe sequences are listed in key resources table. Slides were washed for 15 min in hybridisation wash buffer #1 (10 mM Tris-Cl pH 7.5, 70% formamide, 0.1% BSA) and three times with hybridisation wash buffer #2 (0.1 M Tris- Cl pH 7.5, 0.15 M NaCl, 0.08% Tween 20 (P1379, Sigma-Aldrich)) for 5 min. To the second wash, 0.2 μg/mL DAPI was added, and the wash time increased to 10 min. Finally, the slides were dehydrated and dried as before and mounted with glass coverslips using Mowiol mounting medium. Samples were imaged using an Olympus FluoView Spectral FV1200 Laser Scanning microscope using FV10-ASW software (version 4.2). Cells to be imaged and analyzed were selected in an unbiased manner based on DAPI staining. Image processing and quantification are described below.

exo-FISH & IF quantification

exo-FISH and IF quantification was conducted as previously described in.[37] For exo-FISH: cenFISH or telFISH foci were automatically detected using Fiji (RRID:SCR_002285, v. 2.0.0-rc-69/1.52p)[80] based on an arbitrarily determined threshold (~300–600 arbitrary units). A 10x10-20x20 pixel box was generated around each focus and saved, after which the median signal across all foci of a given cell was calculated to create a ‘median focus’ per cell. The median value of the perimeter readings was then used to estimate the background signal and this was subtracted from the median focus. Representative median foci were plotted using seaborn (v.0.11.1).[82] For beeswarm plotting in Prism (RRID:SCR_002798, v.8.4.3) (www.graphpad.com), the sum of fluorescence signal within the background-subtracted median focus was calculated to encapsulate both focus intensity as well as size. For IF, the same approach as described above was used except CENP-B foci were automatically detected and used to define the 10x10-20x20 pixel box. The background-subtracted signal intensity of CENP-B as well as the secondary signal (e.g., CENP-A) was then calculated within the pixel box defined by CENP-B previously.

Cell survival assay

To validate the HCT116 *Os*TIR1 mAID cell lines, 3000 cells were seeded per well in a 96-well plate in technical triplicate. 24 h post seeding, 500 μM Indole-3-acetic acid (IAA) (I5148, Sigma-Aldrich) was added to the relevant wells and incubated for 2 h to promote protein depletion. The relevant drugs were prepared by serial dilution and added to the required wells along with additional IAA to maintain a final concentration of 500 μM. After 5 days, cells were incubated with complete media containing 10% WST-1 reagent (5015944001, Roche) for 1 h at 37°C. Plate absorbance was measured at 450 nm and 650 nm and cell survival was calculated as 450 nm readings subtracted from the 650 nm background readings and presented as a percentage of the negative control.

To assess the long-term impact of IAA on HCT116 *Os*TIR1 mAID cells, 600 cells were seeded per well into a 6-well plate. Cells were seeded into conditioned media (70% fresh complete media, 30% used media), in triplicate. 24 h post seeding, 500 μM IAA was added to the relevant wells and incubated for 10 days until colony formation. Fresh drug added after 5 days. To visualise colonies, media was removed from the wells and cells washed with PBS. Cells were stained and fixed using Crystal Violet staining solution (0.005% w/v Crystal Violet, 25% v/v methanol, water) for 30 min, protected from light, before washing thoroughly in distilled water. Samples were left to air dry and imaged using a Nikon D3200 camera. Colonies were counted automatically using ImageJ.[79] Colonies were defined through thresholding of 8-bit images and the ‘Analyze Particles’ function quantified the number of colonies displaying 0.01–1.00 circularity. Percentage survival was calculated relative to the untreated control.

The proliferation of hTERT-RPE1 cells was evaluated using the WST-1 cell proliferation assay. Initially, hTERT-RPE1 cells were seeded in a 6-well plate at a density of 6x10^4 cells per well. After 24 h, the cells were transfected with either BRCA2 siRNA, PALB2 siRNA, or non-targeting siRNA using Lipofectamine RNAiMAX (ThermoFisher Scientific). The following day, the cells were transferred to a 96-well plate at a density of 3x10^3 cells per well, following the manufacturer’s protocol. 96 h post-transfection, 10 μL of tetrazolium salt (4-[3-(4-Iodophenyl)-2-(4-nitrophenyl)-2H-5-tetrazolio]-1,3-benzene sulfonate) was added to each well containing 90 μL of culture medium. The cells were then incubated at 37°C with 5% CO2 for 4 h. Absorbance values at 450 nm and 650 nm (background reading) were measured using a microplate reader (Molecular Devices SpectraMax M5). The average absorbance values were calculated, and the background and blank control values were subtracted.

Generation of CRISPR-Cas9 modified HCT116 cell lines

For endogenous mAID-Halo tagging to BRCA2 or PALB2, HCT116 *Os*TIR1 cells were transfected using Lipofectamine LTX (A12621, Thermo Scientific) according to manufacturer’s instructions. 10 μL Lipofectamine LTX and 1677 ng repair template and 3333 ng

Cas9-gRNA and 2.5 μL Plus reagent were mixed and incubated for 15 min at RT. The lipofectamine-DNA mixture was added to cells cultured in antibiotic-free media. 24 h post-transfection, the transfection media was replaced with fresh complete media. Cells were then maintained in culture under selection with 700 μg/mL G418 (G8168, Sigma-Aldrich) for approximately 1 week. To generate monoclonal cell lines, single cells were plated into 96-well plates using fluorescence-activated cell sorting (FACS) (BD FACS Aria III) and were allowed to grow for 2–4 weeks after which growing clones were expanded and screened for successful tagging by western blot. Integration and zygosity was confirmed by PCR using PfuUltra II Fusion HS DNA Polymerase (600670, Agilent Technologies) using the primers listed in key resources table according to manufacturer's recommendations. All DNA concentrations were measured using a NanoPhotometer N60 (Implen) and sequences confirmed by DNA sequencing (Source BioScience UK Ltd).

Generation of hTERT RPE-1 MLH KO cell lines

MLH1 knockout cells were generated using a plasmid-based system followed by single-cell sorting. Briefly, 5′-phosphorylated oligos containing guide-sequences targeting MLH1 were designed and ordered (Integrated DNA technologies), all carrying 4-bp 5′-overhangs complementary to the intended cloning site. Oligos were hybridised *in vitro* using a nuclease-free duplex buffer (11-01-03-01, Integrated DNA technologies) according to manufacturer's instructions. The plasmid backbone (pSpCas9(BB)-2A-GFP (pX458) (plasmid #48138, Addgene)) was then digested using BbsI (R3539, New England Biolabs) followed by heat-inactivation of enzyme and dephosphorylation of DNA ends using Quick CIP (M0525, New England Biolabs). Hybridised oligos were then ligated into the digested backbone using Takara DNA Ligation Kit, Mighty Mix (6023, Takara Bio Europe) before transformation of TOP10 bacteria and amplification of plasmid products. Resulting products were confirmed by agarose gel electrophoresis and Sanger sequencing (Source BioScience). Plasmids with confirmed integration of the current guide sequence were used for transfection of low-passage hTERT RPE-1 cells using Lipofectamine LTX according to manufacturer's instructions. Single-cell sorting based on EGFP-expression was done 24 h after transfections using the BD FACSAria Fusion Flow Cytometer. Resultant clones were then expanded and screened using western blot for MLH1-levels.

RT-qPCR

RNA was extracted from cell pellets flash-frozen in liquid nitrogen using TRI reagent solution (AM9738, ThermoFisher Scientific) as per manufacturer's protocol with slight modifications. Briefly, cell pellets (up to 5,000,000 cells per sample) were resuspended in 200 μL PBS followed by addition of 500 μL TRIzol reagent and 200 μL 1-Bromo-3-chloropropane (BCP) (B9673, Sigma-Aldrich). Samples were mixed by vortexing for 1 min followed by a 5-min incubation at 55°C in a thermal mixing block, then vortexed again for 1 min before centrifugation at 12,000g for 15 min at 4°C. The aqueous phase was collected and added to a fresh tube, followed by addition of 500 μL BCP, another 30 s of vortexing, and centrifugation at 16,100g for 5 min at 4°C. The aqueous phase was again transferred to a fresh tube. RNA was the precipitated by addition of 0.8 volumes ice-cold 2-propanol, ammonium acetate 7.5 M (to a final concentration of 750 mM) (A2706, Sigma-Aldrich), and 1 μL GlycoBlue CoPrecipitant 15 mg/mL (AM9515, Invitrogen) followed by incubation overnight at −20°C. On the following day, samples where centrifuged at 16,100g for 30 min at 4°C. Pellets were vigorously washed twice, first in ice-cold 100% EtOH followed by centrifugation at 16,100g for 5 min at 4°C, then in ice-cold 70% EtOH followed by centrifugation again at 16,100g for 5 min at 4°C. After removing supernatant and air-drying for 10 min, samples were suspended in 50 μL nuclease-free dH2O and diluted to a consistent concentration between samples.

Contaminating genomic DNA was then removed by DNase treatment using the TURBO DNA-free kit (AM1907, Invitrogen) using the rigorous treatment protocol described by the manufacturers. Up to 2 μg RNA was then reverse-transcribed to cDNA using a high-capacity cDNA reverse transcription kit (4368814, Applied Biosystems), including a non-reverse transcribed (RT-) sample to detect any signal contributions from contaminating gDNA. The equivalent of 20 ng cDNA input was then assessed by qPCR using SensiFAST SYBR No-ROX kit (BIO-98005, Meridian Bioscience) on the Rotorgene Q Real-Time PCR System (Qiagen) using Q-Rex software. Amplification conditions included an initial melting step at 95°C for 2 min, followed by 40 cycles of amplification (95°C 5 s, 62°C 10 s, 72°C 20 s) and a final melting step (50°C–98°C). Technical triplicates were performed for each reading. Centromeric alpha-satellite RNA expression levels were normalised to the GAPDH mRNA expression level using the $2-\Delta\Delta Ct$ method. Primers used are listed in key resources table.

QUANTIFICATION AND STATISTICAL ANALYSIS

Excel and GraphPad PRISM software (version 7 and 9) were used for data analysis and statistical analysis, following the GraphPad Statistics Guide. Data were expressed as mean ± SEM. n represents the total number of cells (or events) analyzed. p-values <0.05 denote statistical significance. Levels of statistical significance were represented as follows: $p < 0.05$ (*), $p < 0.01$ (**), $p < 0.001$ (***), $p < 0.0001$ (****). Automated imaging analysis scripts available upon request. Python (RRID:SCR_008394, v.3.8.8),[83] Matplotlib (RRID:SCR_008624, v.3.4.2),[84] NumPy (RRID:SCR_008633, v.1.19.1),[85] pandas (RRID:SCR_018214, v1.2.3)[86] and seaborn (v.0.11.1)[82] were all used for data processing and visualisation.

Cell Reports, Volume *44*

Supplemental information

The homologous recombination factors BRCA2 and PALB2 interplay with mismatch repair pathways to maintain centromere stability and cell viability

Emily Graham, Lucia Rampazzo, Chin Wei Brian Leung, Jacob Wall, Emőke Zsanett Gerőcz, Mikhail Liskovykh, Nikolay Goncharov, Xanita Saayman, Ramazan Gundogdu, Masato T. Kanemaki, Hiroshi Masumoto, Vladimir Larionov, Natalay Kouprina, and Fumiko Esashi

Supplemental Figures

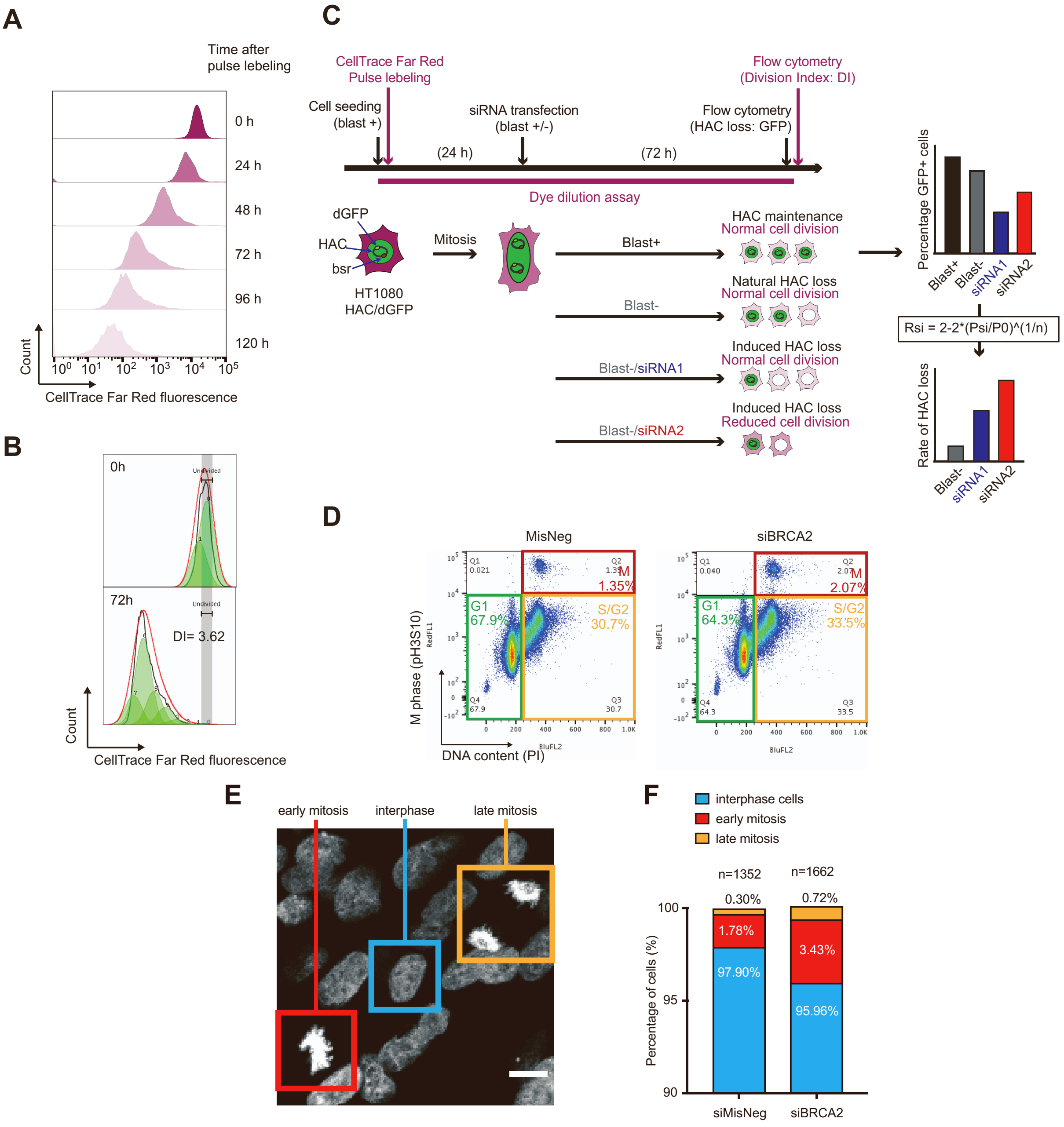

Figure S1. Cell cycle effect upon BRCA2 depletion in HT1080 cells, Related to Figure 1

A. Flow cytometry dye dilution assay proof of principle. Fluorescent intensity of the cell dye is diluted with every cell division. **B.** Representative image of the DI determined from the dye dilution assay. **C.** Schematic of HAC loss assay combined with the dye dilution assay. Blasticidin-containing medium selects for HAC-containing cells. 72 hours post-siRNA treatment, GFP signal was measured by flow cytometry. **D.** Cell cycle profile upon 72 hours siMisNeg and siBRCA2 treatment after staining with Propidium iodide (PI) and pH3 S10. **E.** Representative image of DAPI staining in HT1080 alphoid^{tetO} dGFP-HAC cells, 72 hours post BRCA2 depletion. Interphase (blue), early mitosis (red), late mitosis (yellow). Scale bar equals 10 µm. **F.** Graph displaying the percentage of cells in different stages of cell cycle in control and BRCA2-depleted cells. n is the number of cells analysed for each condition in a single experiment.

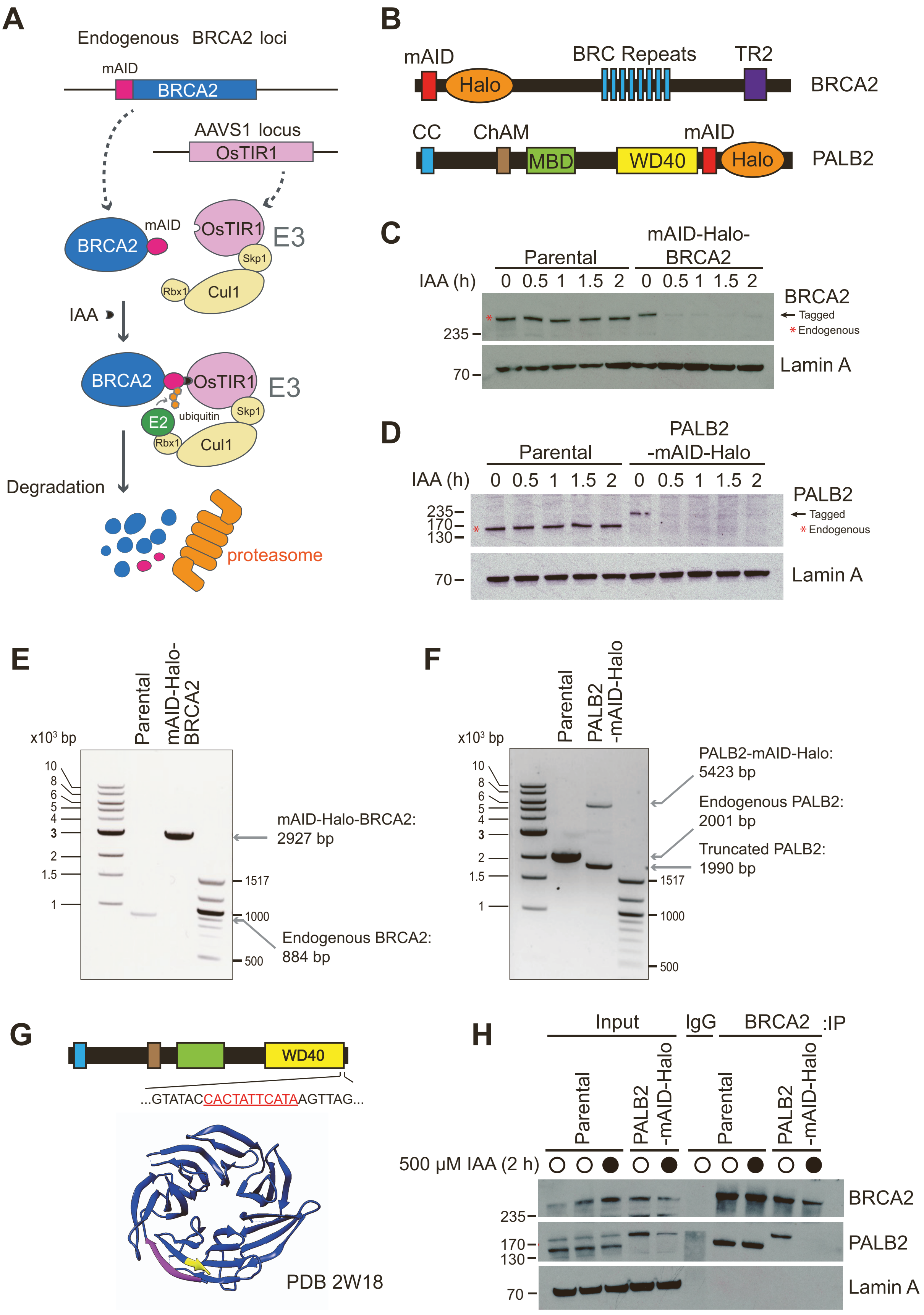

Figure S2. Generation and validation of auxin-inducible degron (AID) system for depleting BRCA2 or PALB2 in HCT116 cells, Related to Figures 2 and 3

A. Schematic of auxin-inducible degron (AID) system to degrade proteins of interest. **B.** Schematic of BRCA2 and PALB2 mAID-Halo tagging. mAID, mini Auxin-inducible degron. CC, Coiled-coil. ChAM, Chromatin-association motif. MBD, MRG15-binding domain. **C.** Western blot showing degradation of BRCA2 within 30 minutes of 500 μM IAA treatment. Lamin A used as a loading control. **D.** Western blot showing degradation of PALB2 within 30 minutes of 500 μM IAA treatment. Red asterisk refers to endogenous PALB2 protein. Lamin A used as a loading control. **E-F.** Agarose gel image of PCR validating N-terminal tagging of BRCA2 (E) or C-terminal tagging of PALB2 (F) with mAID and Halo. The expected size of each amplified DNA fragments are displayed. Asterisk marks band sent for sequencing. **G.** Schematic of the deleted allele of PALB2 gene, where the deleted nucleotides are indicated in red. Crystal structure of PALB2 WD40 domain shows that C-terminal three amino acids (yellow highlight) interact with the N-terminal beta-sheet (pink highlight) for stability, hence the truncation destabilises the WD40 domain and full-length PALB2. **H.** Immunoprecipitation showing loss of PALB2-BRCA2 interaction upon 2 hours 500 μM IAA treatment. Lamin A used as a loading control for the inputs.

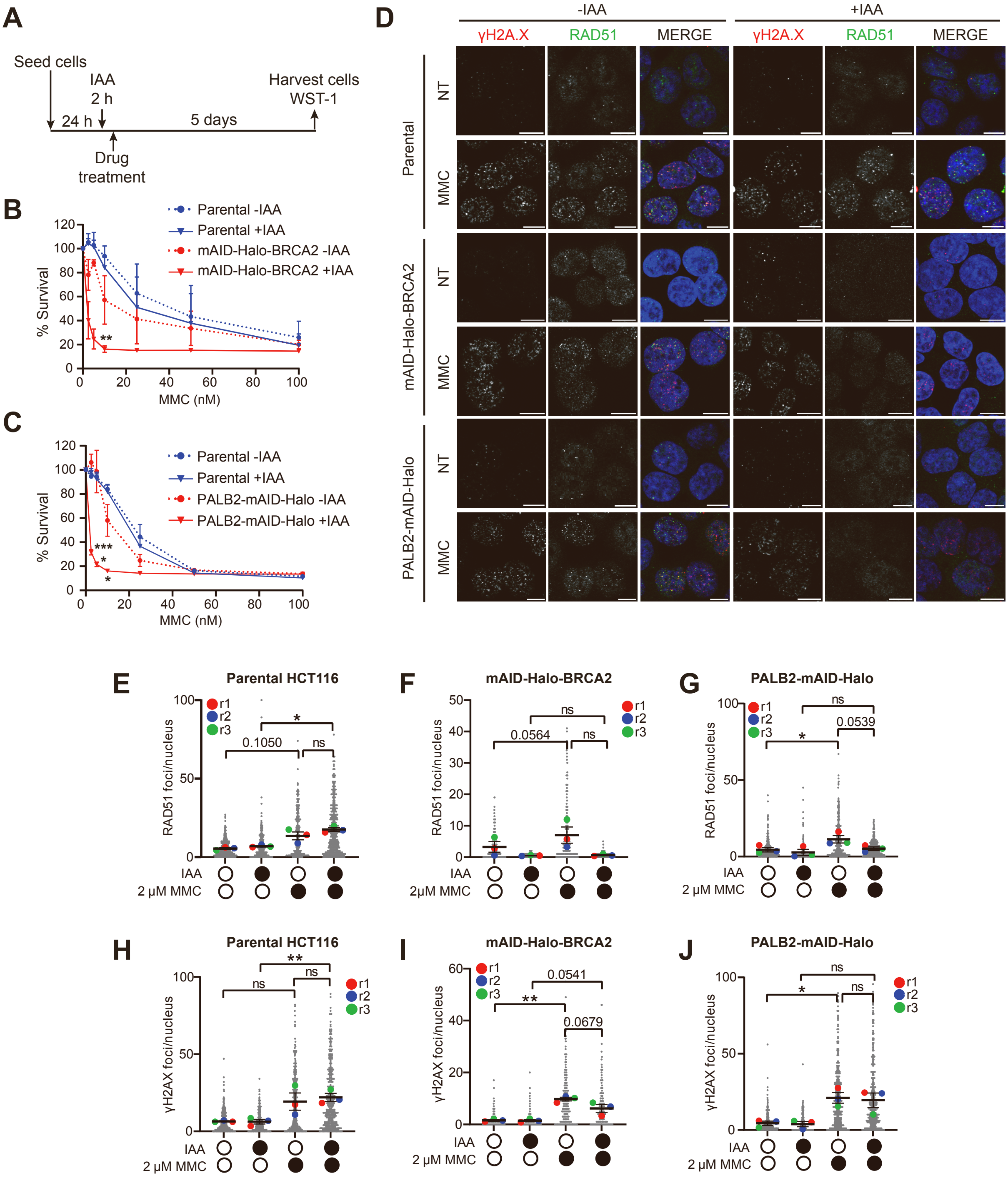

Figure S3. AID-mediated BRCA2 and PALB2 depletion sensitises HCT116 cells to DNA-damaging agents, Related to Figures 2 and 3

A. Schematic of WST-1 protocol. **B.** Graph showing percentage cell survival after MMC and 500 μM IAA treatment in HCT116 *Os*TIR1 parental and mAID-BRCA2 cell lines. Points represent average of three independent experiments. Error bars represent S.E.M. Two-tailed T-test comparing -IAA vs +IAA for each cell line. **C.** Graph showing percentage cell survival after MMC and 500 μM IAA treatment in HCT116 *Os*TIR1 parental and PALB2-mAID cell lines. Points represent average of three independent experiments. Error bars represent S.E.M. Two-tailed t-test comparing -IAA vs +IAA for each cell line. **D.** Representative images RAD51 and γ-H2A.X signalling after BRCA2 and PALB2 depletion in HCT116 *Os*TIR1 degron cells. Scale bar equals 10 μm. **E-G.** Quantification of RAD51 foci count per nucleus after MMC treatment in HCT116 *Os*TIR1 parental (panel E), mAID-BRCA2 (panel F), and PALB2-mAID cells (panel G). Cells were first treated with IAA for 2 hours, followed by MMC treatment

for 2 hours. At least 100 cells were quantified per condition, per repeat. Error bars represent S.E.M. Paired t-test. **H-J.** Quantification of γ-H2A.X foci count per nucleus after MMC treatment in HCT116 *Os*TIR1 parental (panel H), mAID-BRCA2 (panel I), and PALB2-mAID cells (panel J). Cells were first treated with IAA for 2 hours, followed by MMC treatment for 2 hours. At least 100 cells were quantified per condition, per repeat. Error bars represent S.E.M. Paired t-test. ns represents no statistical significance; $p<0.05$ (*), $p<0.01$ (**), $p<0.001$ (***).

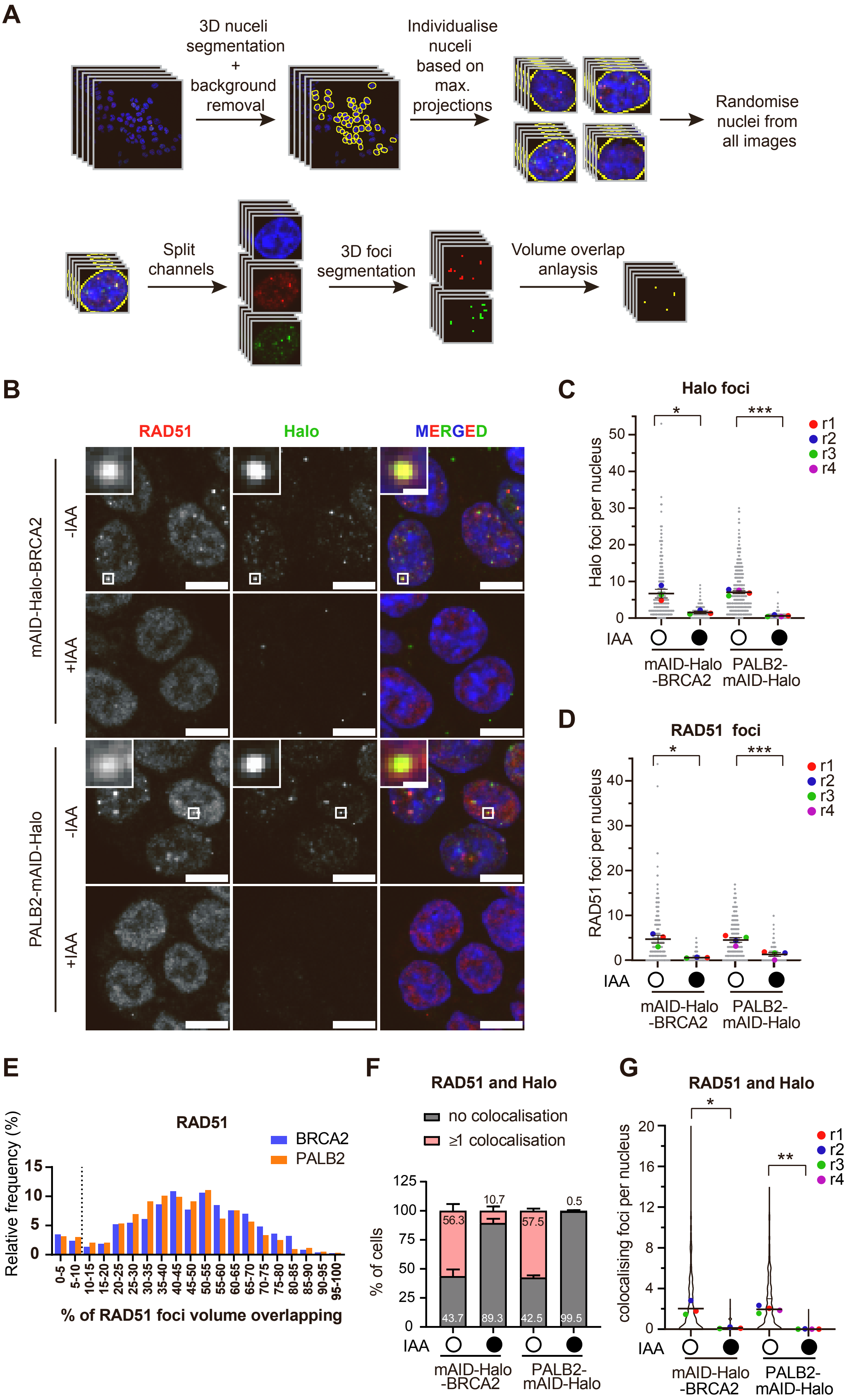

A
3D nuceli segmentation + background removal
Individualise nuceli based on max. projections
Randomise nuclei from all images
Split channels
3D foci segmentation
Volume overlap anlaysis
B
RAD51
Halo
MERGED
mAID-Halo-BRCA2
PALB2-mAID-Halo
-IAA
+IAA
C
Halo foci
Halo foci per nucleus
*
***
r1
r2
r3
r4
IAA
mAID-Halo -BRCA2
PALB2- mAID-Halo
D
RAD51 foci
RAD51 foci per nucleus
E
RAD51
BRCA2
PALB2
Relative frequency (%)
% of RAD51 foci volume overlapping
F
RAD51 and Halo
no colocalisation
≥1 colocalisation
% of cells
56.3
10.7
57.5
0.5
43.7
89.3
42.5
99.5
G
colocalising foci per nucleus
**

Figure S4. Visualisation of endogenous BRCA2 and PALB2 using the HaloTag, Related to Figures 2 and 3

A. Schematic of the volume overlap analysis pipeline. **B.** Representative images of Halo ligand JF549 (green) and RAD51 (red) in HCT116 mAID-Halo-BRCA2 and PALB2-mAID-Halo cell lines. DNA staining by Hoechst shown in blue. Scale bar equals to 5 µm, inset scale bar equals to 1 µm). **C.** Number of Halo foci after IAA-mediated depletion using 3D segmentation pipeline. Each datapoint represents one nucleus, coloured dots are means of the replicates. Error bars represent S.E.M. Paired t-test between the means of 3 replicates. **D.** Number of RAD51 foci detected using 3D segmentation pipeline. Each datapoint represents one nucleus, coloured dots are means of the replicates. Error bars represent S.E.M. Paired t-test between the means of 3 replicates. **E.** Percentage of RAD51 focus volume overlapping with Halo-BRCA2 (blue) and PALB2-Halo (orange) foci. Values represent the relative frequency of the amount of overlap from all colocalisation events from the non-treated condition. Data also displayed in Figure 2E. **F.** Percentage of nuclei with none or at least one colocalisation event between CENP-B and Halo. At least 3 biological replicates with 100 cells analysed in each condition per replicate. Error bars represent S.E.M. **G.** Number of colocalisation events per nuclei. Coloured dots represent the means of the replicates. Error bars represent S.E.M. Paired t-test. ns represents no statistical significance; p<0.05 (*), p<0.01 (**), p<0.001 (***).

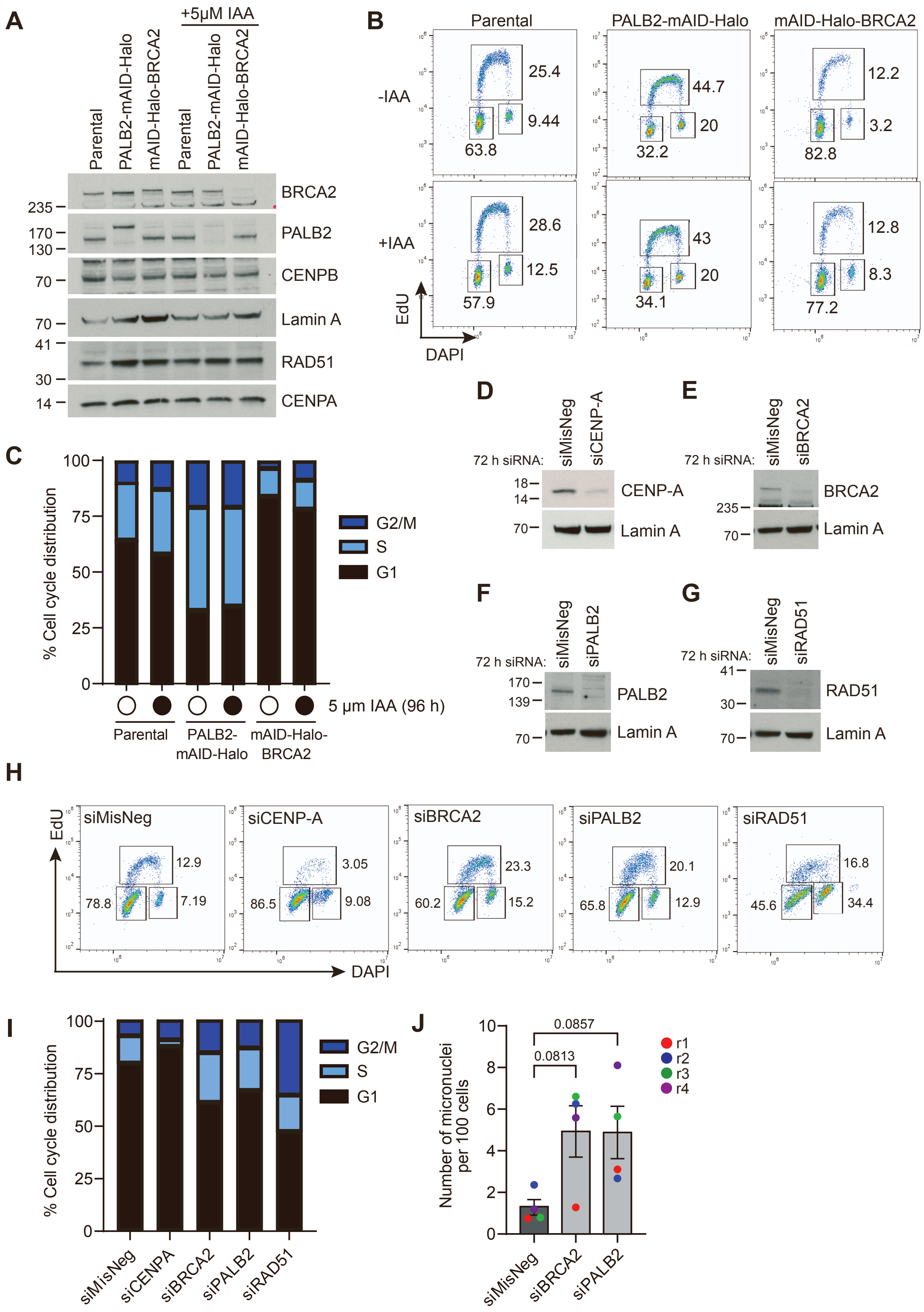

Figure S5. The depletion of BRCA2 or PALB2 confer little impact on cell cycle progression, Related to Figures 4 and 5

A. Western blot showing PALB2 and BRCA2 degradation after 5 μM IAA treatment for 96 hours in HCT116 *Os*TIR1 cells. Lamin A used as a loading control. **B.** Cell cycle analysis by flow cytometry after 96 hours 5 μM IAA treatment and EdU and DAPI staining in HCT116 *Os*TIR1 cells. Cells in G1, S, and G2/M phase are gated and percentage of cells in each gate displayed. **C.** Graph displaying quantification of the percentage of cells in each cell cycle stage after IAA depletion, as shown in panel B. **D-G.** Depletion of CENP-A (panel D), BRCA2 (panel E), PALB2 (panel F), and RAD51 (panel G) in RPE1 cells using siRNA for 72 hours. siMisNeg was used as a negative control. Lamin A was used as a loading control. **H.** Cell cycle analysis by flow cytometry of RPE1 cells after 96 hours siRNA depletion and EdU and DAPI staining. Cells in G1, S, and G2/M phase are gated and percentage of cells in each gate displayed. **I.** Graph displaying quantification of the percentage of cells in each cell cycle stage after siRNA depletion, as shown in panel H **J.** Number of micronuclei per 100 cells of RPE1 cells after siRNA-mediated depletion of BRCA2 and PALB2. At least 100 cells analysed per condition, per repeat. Error bars represent S.E.M. RM one-way ANOVA with Tukey's multiple comparisons test.

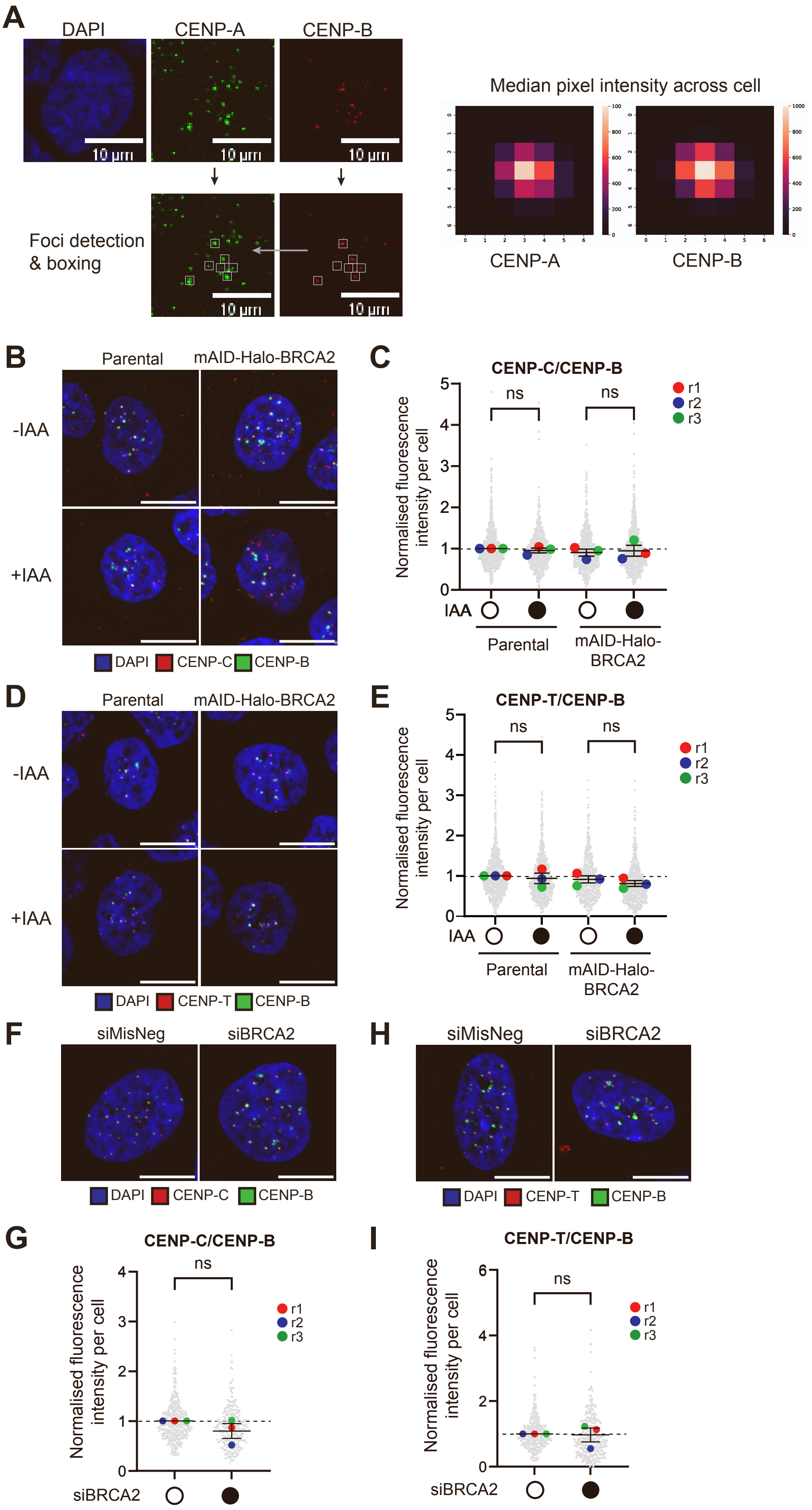
A
DAPI
CENP-A
CENP-B
10 µm
Foci detection & boxing
Median pixel intensity across cell
CENP-A
CENP-B
B
Parental
mAID-Halo-BRCA2
-IAA
+IAA
DAPI CENP-C CENP-B
C
CENP-C/CENP-B
ns
ns
r1
r2
r3
Normalised fluorescence intensity per cell
IAA
Parental
mAID-Halo-BRCA2
D
Parental
mAID-Halo-BRCA2
-IAA
+IAA
DAPI CENP-T CENP-B
E
CENP-T/CENP-B
ns
ns
r1
r2
r3
Normalised fluorescence intensity per cell
IAA
Parental
mAID-Halo-BRCA2
F
siMisNeg
siBRCA2
DAPI CENP-C CENP-B
H
siMisNeg
siBRCA2
DAPI CENP-T CENP-B
G
CENP-C/CENP-B
ns
r1
r2
r3
Normalised fluorescence intensity per cell
siBRCA2
I
CENP-T/CENP-B
ns
r1
r2
r3
Normalised fluorescence intensity per cell
siBRCA2

Figure S6. BRCA2 depletion confers no detectable impact on the levels of CENP-C and CENP-T at centromeres, Related to Figure 4

A. Schematic of centromere component quantification. **B.** Representative images of CENP-C and CENP-B immunofluorescence staining in HCT116 *Os*TIR1 parental or mAID-Halo-BRCA2 cells, with or without treatment of IAA. Scale bar equals 10 µm. **C.** Normalised CENP-C to CENP-B signal in HCT116 *Os*TIR1 parental or mAID-Halo-BRCA2 cells. RM one-way ANOVA with Tukey's multiple comparisons test. **D.** Representative images of CENP-T and CENP-B immunofluorescence staining in HCT116 *Os*TIR1 parental or mAID-Halo-BRCA2 cells, with or without treatment of IAA. Scale bar equals 10 µm. **E.** Normalised CENP-T to CENP-B signal in parental or mAID-Halo-BRCA2 HCT116 cells. RM one-way ANOVA with Tukey's multiple comparisons test. **F.** Representative images of CENP-C and CENP-B immunofluorescence staining in RPE1 cells with 96 hours siRNA treatment. Scale bar equals 10 µm. **G.** Normalised CENP-C signal to CENP-B in RPE1 cells after siRNA treatment, 96 hours. Paired t-test. **H.** Representative images of CENP-T and CENP-B immunofluorescence staining in RPE1 cells with 96 hours siRNA treatment. Scale bar equals 10 µm. **I.** Normalised CENP-T signal to CENP-B in RPE1 cells after 96 hours siRNA treatment. For each measurement, signal was normalised to the siMisNeg control. n=3. At least 100 cells analysed per condition, per repeat. Large coloured dots represent the mean of each repeat. Error bars represent S.E.M. Paired t-test. ns represents no statistical significance.

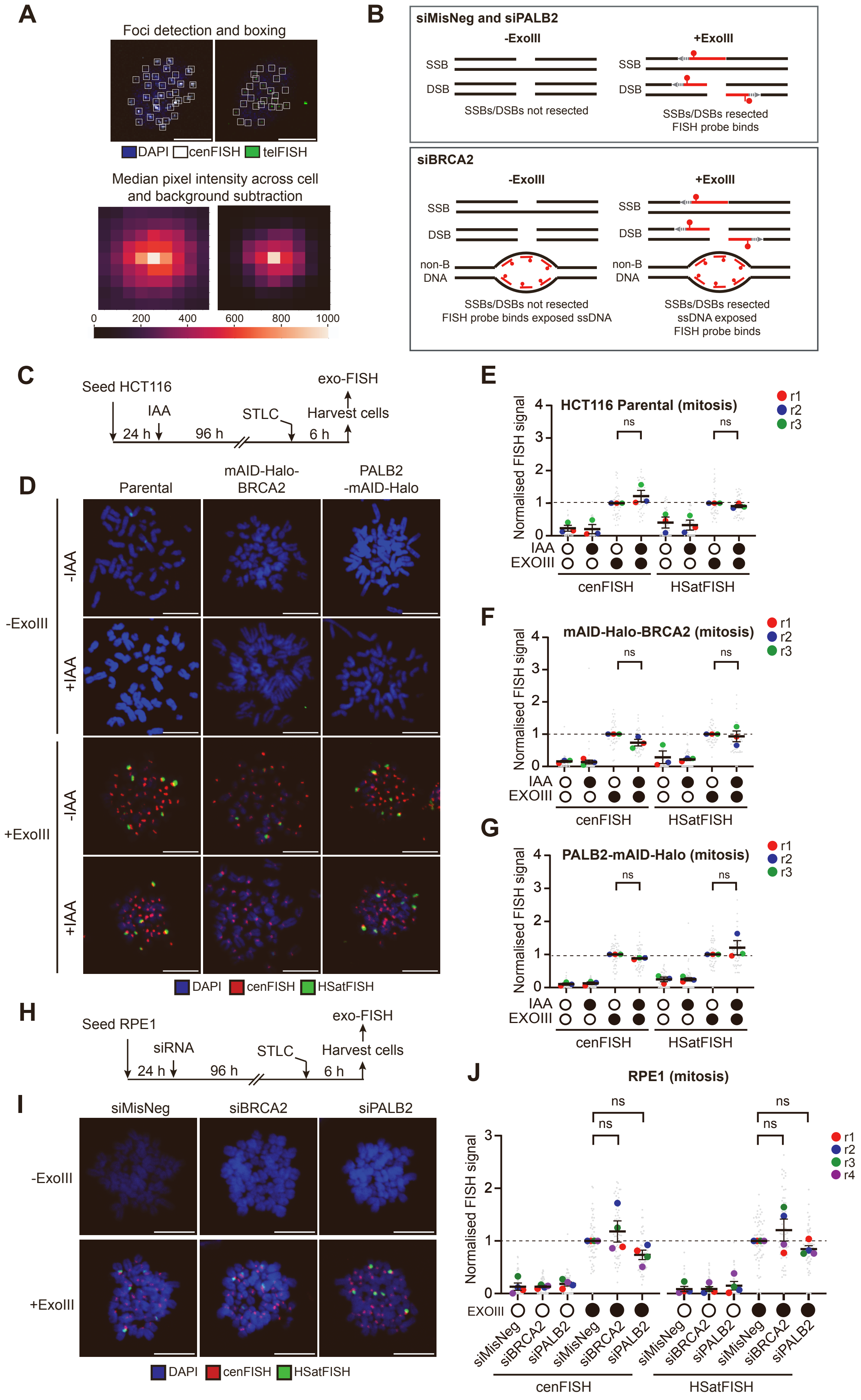

A
Foci detection and boxing
DAPI cenFISH telFISH
Median pixel intensity across cell
and background subtraction
0 200 400 600 800 1000
B
siMisNeg and siPALB2
-ExoIII
+ExoIII
SSB
DSB
SSBs/DSBs not resected
SSBs/DSBs resected
FISH probe binds
siBRCA2
non-B DNA
SSBs/DSBs not resected
FISH probe binds exposed ssDNA
SSBs/DSBs resected
ssDNA exposed
FISH probe binds
C
Seed HCT116
IAA
24 h
96 h
STLC
exo-FISH
Harvest cells
6 h
D
Parental
mAID-Halo-BRCA2
PALB2-mAID-Halo
-ExoIII
+ExoIII
-IAA
+IAA
DAPI cenFISH HSatFISH
E
HCT116 Parental (mitosis)
Normalised FISH signal
ns
r1
r2
r3
IAA
EXOIII
cenFISH
HSatFISH
F
mAID-Halo-BRCA2 (mitosis)
G
PALB2-mAID-Halo (mitosis)
H
Seed RPE1
siRNA
24 h
96 h
STLC
exo-FISH
Harvest cells
6 h
I
siMisNeg
siBRCA2
siPALB2
-ExoIII
+ExoIII
DAPI cenFISH HSatFISH
J
RPE1 (mitosis)
Normalised FISH signal
ns
r1
r2
r3
r4
EXOIII
siMisNeg siBRCA2 siPALB2 siMisNeg siBRCA2 siPALB2
cenFISH
HSatFISH

Figure S7. The depletion of BRCA2 or PALB2 confer no detectable impact on centromere DNA breaks in mitosis, Related to Figure 5

A. Workflow of exo-FISH analysis. Scale bar equals 10 µm. **B.** Model showing source of FISH signal under different conditions in siRNA-treated RPE1 cells. In siMisNeg or siPALB2 treated samples, FISH signal is low in -ExoIII conditions, reflecting no resection of ssDNA/dsDNA. Upon ExoIII treatment, breaks are resected and resultant ssDNA is bound by cenFISH probes. In siBRCA2 treated samples, FISH signal is high even in the absence of ExoIII treatment, potentially reflecting the accumulation of secondary DNA structures, such as DNA bubbles and non-B form DNA, exposing ssDNA for FISH probe binding. Upon ExoIII treatment, DNA breaks are resected, and FISH probes bind. Secondary DNA structures are break-independent and unaffected by ExoIII treatment. **C.** Schematic of experimental protocol for exo-FISH in mitotic HCT116 *Os*TIR1 degron cells. **D.** Representative images of exo-FISH. Scale bar equals 10 µm. **E.** exo-FISH in HCT116 *Os*TIR1 parental cells with or without 5 µM IAA for 96 hours. RM one-way ANOVA with Tukey's multiple comparisons test. **F.** exo-FISH in HCT116 mAID-BRCA2 cells with or without 5 µM IAA for 96 hours. RM one-way ANOVA with Tukey's multiple comparisons test. **G.** exo-FISH in HCT116 mAID-PALB2 cells with or without 5 µM IAA for 96 hours. cenFISH and HSatFISH signal normalised to +IAA, +ExoIII treated samples. Large coloured dots represent average of each independent experiment. n=3. At least 15 cells analysed per condition, per repeat. Error bars represent S.E.M. RM one-way ANOVA with Tukey's multiple comparisons test. **H.** Schematic of siRNA treatment protocol for exo-FISH in mitotic RPE1 cells. **I.** Representative images of exo-FISH after 96 hours siRNA treatment in RPE1 cells. Scale bar equals 10 µm. **J.** exo-FISH of mitotic RPE1 cells with or without BRCA2 or PALB2 siRNA mediated depletion. Samples were normalised to siMisNeg cenFISH or HSatFISH signal without ExoIII treatment. Large coloured datapoints represent the averages of each independent repeat. n=3. At least 15 cells analysed per condition, per repeat. Error bars represent S.E.M. RM one-way ANOVA with Tukey's multiple comparisons test. ns represents no statistical significance.
